# Supplementary material for: The Phosphoglycerate Kinase (PGK) Gene Family of Maize (Zea mays var. B73)
Source: Plants (Basel). 2020 Nov 24;9(12):1639. doi: 10.3390/plants9121639 (PMC7761438; doi:10.3390/plants9121639)

Figure S1 Global alignment of all PGK proteins from publicly available plant and animal databases**.** Alignment parameters were gap cost = 40, gap extension = 10, and gap end as any other. The different colors, according to RasMol color coding, highlight the chemical properties of residues: red = negatively charged, dark blue = positively charged, light blue = neutral, green = hydrophobic (green), yellow = sulphur-containing, etc). The sequence conservation = a percentage bar-score (bottom). The sequence logo summarises the occurrence of given amino acids at specific positions (very bottom)

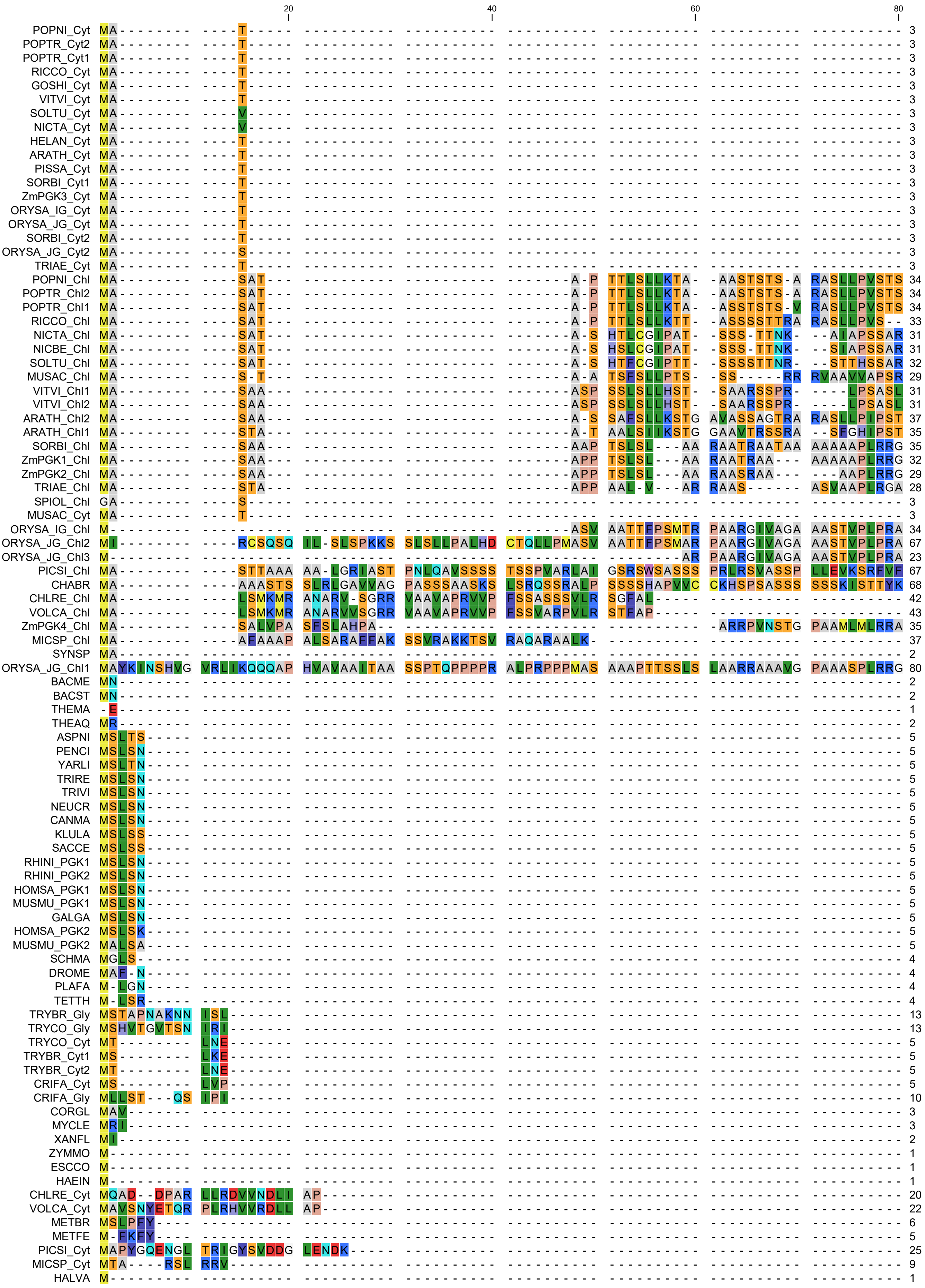
20
40
60
80
POPNI_Cyt
POPTR_Cyt2
POPTR_Cyt1
RICCO_Cyt
GOSHI_Cyt
VITVI_Cyt
SOLTU_Cyt
NICTA_Cyt
HELAN_Cyt
ARATH_Cyt
PISSA_Cyt
SORBI_Cyt1
ZmPGK3_Cyt
ORYSA_IG_Cyt
ORYSA_JG_Cyt
SORBI_Cyt2
ORYSA_JG_Cyt2
TRIAE_Cyt
POPNI_Chl
POPTR_Chl2
POPTR_Chl1
RICCO_Chl
NICTA_Chl
NICBE_Chl
SOLTU_Chl
MUSAC_Chl
VITVI_Chl1
VITVI_Chl2
ARATH_Chl2
ARATH_Chl1
SORBI_Chl
ZmPGK1_Chl
ZmPGK2_Chl
TRIAE_Chl
SPIOL_Chl
MUSAC_Cyt
ORYSA_IG_Chl
ORYSA_JG_Chl2
ORYSA_JG_Chl3
PICSI_Chl
CHABR
CHLRE_Chl
VOLCA_Chl
ZmPGK4_Chl
MICSP_Chl
SYNSP
ORYSA_JG_Chl1
BACME
BACST
THEMA
THEAQ
ASPNI
PENCI
YARLI
TRIRE
TRIVI
NEUCR
CANMA
KLULA
SACCE
RHINI_PGK1
RHINI_PGK2
HOMSA_PGK1
MUSMU_PGK1
GALGA
HOMSA_PGK2
MUSMU_PGK2
SCHMA
DROME
PLAFA
TETTH
TRYBR_Gly
TRYCO_Gly
TRYCO_Cyt
TRYBR_Cyt1
TRYBR_Cyt2
CRIFA_Cyt
CRIFA_Gly
CORGL
MYCLE
XANFL
ZYMMO
ESCCO
HAEIN
CHLRE_Cyt
VOLCA_Cyt
METBR
METFE
PICSI_Cyt
MICSP_Cyt
HALVA

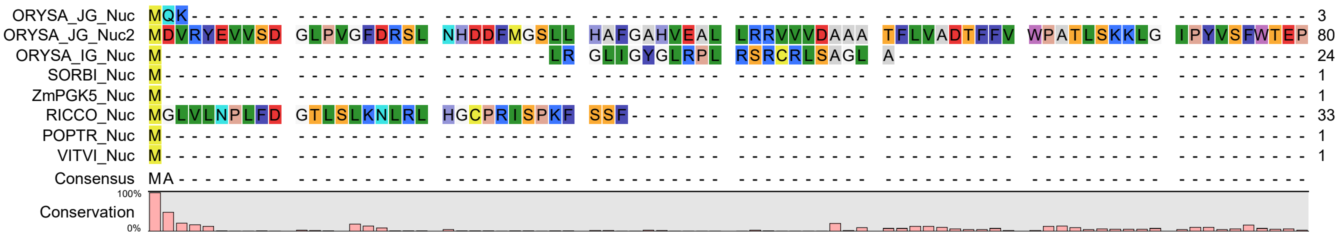
ORYSA_JG_Nuc MQK------- ---------- ---------- ---------- ---------- ---------- ---------- ---------- 3
ORYSA_JG_Nuc2 MDVRYEVVSD GLPVGFDRSL NHDDFMGSLL HAFGAHVEAL LRRVVVDAAA TFLVADTFFV WPATLSKKLG IPYVSFWTEP 80
ORYSA_IG_Nuc M--------- ---------- --------LR GLIGYGLRPL RSRCRLSAGL A--------- ---------- ---------- 24
SORBI_Nuc M--------- ---------- ---------- ---------- ---------- ---------- ---------- ---------- 1
ZmPGK5_Nuc M--------- ---------- ---------- ---------- ---------- ---------- ---------- ---------- 1
RICCO_Nuc MGLVLNPLFD GTLSLKNLRL HGCPRISPKF SSF------- ---------- ---------- ---------- ---------- 33
POPTR_Nuc M--------- ---------- ---------- ---------- ---------- ---------- ---------- ---------- 1
VITVI_Nuc M--------- ---------- ---------- ---------- ---------- ---------- ---------- ---------- 1
Consensus MA-------- ---------- ---------- ---------- ---------- ---------- ---------- ----------
100%
Conservation
0%

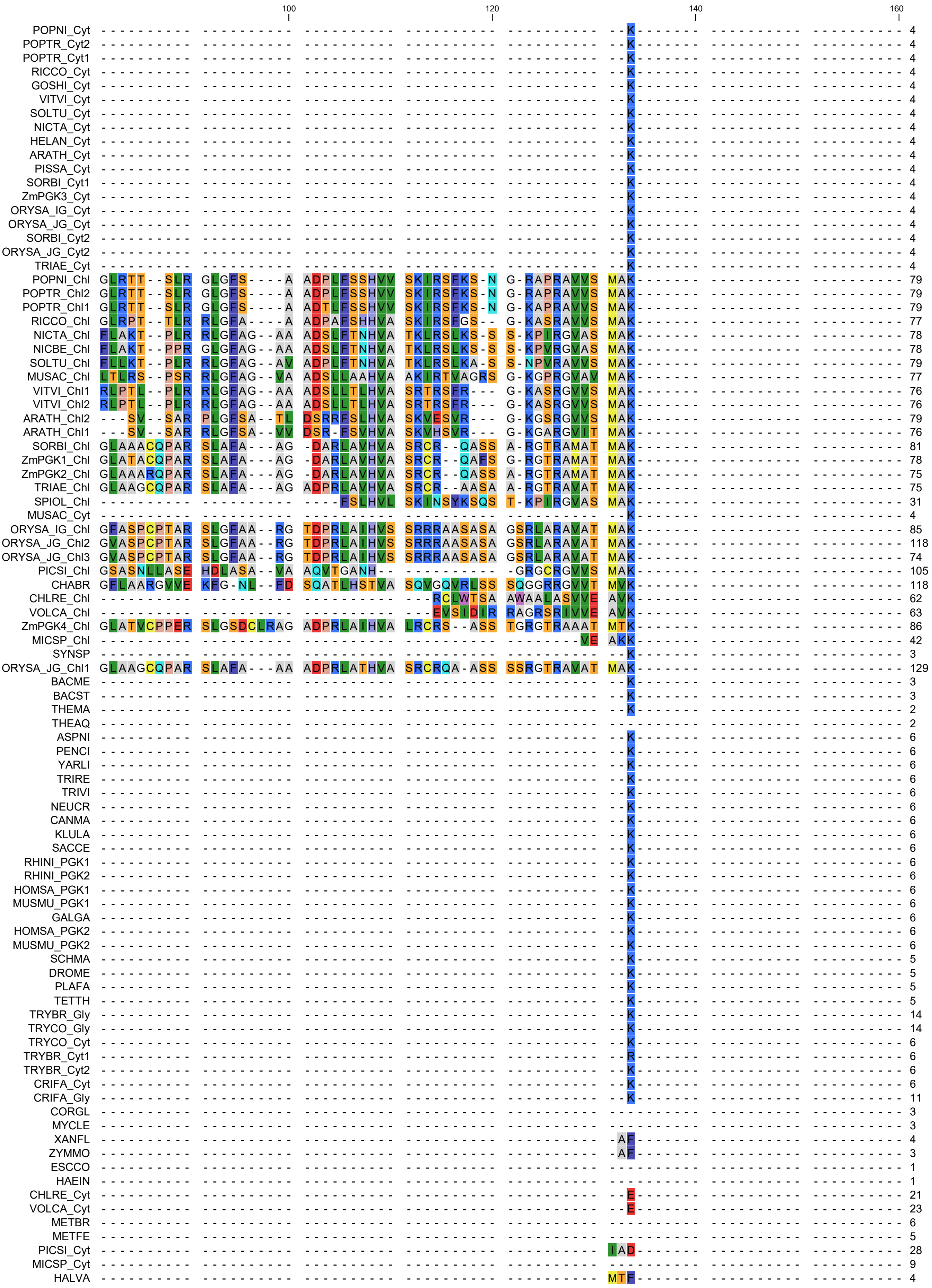
100 120 140 160
POPNI_Cyt K 4
POPTR_Cyt2 K 4
POPTR_Cyt1 K 4
RICCO_Cyt K 4
GOSHI_Cyt K 4
VITVI_Cyt K 4
SOLTU_Cyt K 4
NICTA_Cyt K 4
HELAN_Cyt K 4
ARATH_Cyt K 4
PISSA_Cyt K 4
SORBI_Cyt1 K 4
ZmPGK3_Cyt K 4
ORYSA_IG_Cyt K 4
ORYSA_JG_Cyt K 4
SORBI_Cyt2 K 4
ORYSA_JG_Cyt2 K 4
TRIAE_Cyt K 4
POPNI_Chl GLRTT--SLR GLGFS----A ADPLFSSHVV SKIRSFKS-N G-RAPRAVVS MAK 79
POPTR_Chl2 GLRTT--SLR GLGFS----A ADPLFSSHVV SKIRSFKS-N G-RAPRAVVS MAK 79
POPTR_Chl1 GLRTT--SLR GLGFS----A ADTLFSSHVV SKIRSFKS-N G-KAPRAVVS MAK 79
RICCO_Chl GLRPT--TLR RLGFA----A ADPAFSHHVA SKIRSFGS-- G-KASRAVVS MAK 77
NICTA_Chl FLAKT--PLR RLGFAG--AA ADSLFTNHVA TKLRSLKS-S S-KPIRGVAS MAK 78
NICBE_Chl FLAKT--PPR GLGFAG--AA ADSLFTNHVA TKLRSLKS-S S-KPVRGVAS MAK 78
SOLTU_Chl FLLKT--PLR RLGFAG--AV ADPLFTNHVA TKLRSLKA-S S-NPVRAVVS MAK 79
MUSAC_Chl LTLRS--PSR RLGFAG--VA ADSLLAAHVA AKIRTVAGRS G-KGPRGVAV MAK 77
VITVI_Chl1 RLPTL--PLR RLGFAG--AA ADSLLTLHVA SRTRSFR--- G-KASRGVVS MAK 76
VITVI_Chl2 RLPTL--PLR RLGFAG--AA ADSLLTLHVA SRTRSFR--- G-KASRGVVS MAK 76
ARATH_Chl2 ---SV--SAR PLGFSA--TL DSRRFSLHVA SKVESVR--- G-KGSRGVVS MAK 79
ARATH_Chl1 ---SV--SAR RLGFSA--VV DSR-FSVHVA SKVHSVR--- G-KGARGVIT MAK 76
SORBI_Chl GLAAACQPAR SLAFA---AG -DARLAVHVA SRCR--QASS A-RGTRAMAT MAK 81
ZmPGK1_Chl GLATACQPAR SLAFA---AG -DARLAVHVA SRCR--QAFS G-RGTRAMAT MAK 78
ZmPGK2_Chl GLAAARQPAR SLAFA---AG -DARLAVHVA SRCR--QASS A-RGTRAMAT MAK 75
TRIAE_Chl GLAAGCQPAR SLAFA---AG ADPRLAVHVA SRCR--AASA A-RGTRAVAT MAK 75
SPIOL_Chl ----FSLHVL SKINSYKSQS T-KPIRGVAS MAK 31
MUSAC_Cyt K 4
ORYSA_IG_Chl GFASPCPTAR SLGFAA--RG TDPRLAIHVS SRRRAASASA GSRLARAVAT MAK 85
ORYSA_JG_Chl2 GVASPCPTAR SLGFAA--RG TDPRLAIHVS SRRRAASASA GSRLARAVAT MAK 118
ORYSA_JG_Chl3 GVASPCPTAR SLGFAA--RG TDPRLAIHVS SRRRAASASA GSRLARAVAT MAK 74
PICSI_Chl GSASNLLASE HDLASA--VA AQVTGANH-- -GRGCRGVVS MAK 105
CHABR GFLAARGVVE KFG-NL--FD SQATLHSTVA SQVGQVRLSS SQGGRRGVVT MVK 118
CHLRE_Chl ---RCLWTSA AWAALASVVE AVK 62
VOLCA_Chl ---EVSIDIR RAGRSRIVVE AVK 63
ZmPGK4_Chl GLATVCPPER SLGSDCLRAG ADPRLAIHVA LRCRS--ASS TGRGTRAAAT MTK 86
MICSP_Chl VE AKK 42
SYNSP K 3
ORYSA_JG_Chl1 GLAAGCQPAR SLAFA---AA ADPRLATHVA SRCRQA-ASS SSRGTRAVAT MAK 129
BACME K 3
BACST K 3
THEMA K 2
THEAQ 2
ASPNI K 6
PENCI K 6
YARLI K 6
TRIRE K 6
TRIVI K 6
NEUCR K 6
CANMA K 6
KLULA K 6
SACCE K 6
RHINI_PGK1 K 6
RHINI_PGK2 K 6
HOMSA_PGK1 K 6
MUSMU_PGK1 K 6
GALGA K 6
HOMSA_PGK2 K 6
MUSMU_PGK2 K 6
SCHMA K 5
DROME K 5
PLAFA K 5
TETTH K 5
TRYBR_Gly K 14
TRYCO_Gly K 14
TRYCO_Cyt K 6
TRYBR_Cyt1 R 6
TRYBR_Cyt2 K 6
CRIFA_Cyt K 6
CRIFA_Gly K 11
CORGL 3
MYCLE 3
XANFL AF 4
ZYMMO AF 3
ESCCO 1
HAEIN 1
CHLRE_Cyt E 21
VOLCA_Cyt E 23
METBR 6
METFE 5
PICSI_Cyt IAD 28
MICSP_Cyt 9
HALVA MTF 4

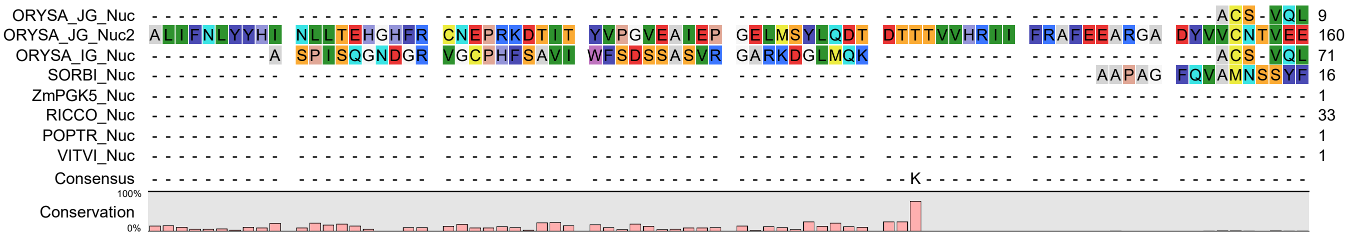
ORYSA_JG_Nuc ---------- ---------- ---------- ---------- ---------- ---------- ---------- ---ACS-VQL 9
ORYSA_JG_Nuc2 ALIFNLYYHI NLLTEHGHFR CNEPRKDTIT YVPGVEAIEP GELMSYLQDT DTTTVVHRII FRAFEEARGA DYVVCNTVEE 160
ORYSA_IG_Nuc ---------A SPISQGNDGR VGCPHFSAVI WFSDSSASVR GARKDGLMQK ---------- ---------- ---ACS-VQL 71
SORBI_Nuc ---------- ---------- ---------- ---------- ---------- ---------- -----AAPAG FQVAMNSSYF 16
ZmPGK5_Nuc ---------- ---------- ---------- ---------- ---------- ---------- ---------- ---------- 1
RICCO_Nuc ---------- ---------- ---------- ---------- ---------- ---------- ---------- ---------- 33
POPTR_Nuc ---------- ---------- ---------- ---------- ---------- ---------- ---------- ---------- 1
VITVI_Nuc ---------- ---------- ---------- ---------- ---------- ---------- ---------- ---------- 1
Consensus ---------- ---------- ---------- ---------- ---------- --K------- ---------- ----------
Conservation
100%
0%

```
                                                     180                           200                           220                           240
                                                       |                             |                             |                             |
POPNI_Cyt      ---------- ---------- ---------- ---------- ---------- ---------- ---------- ---------- 4
POPTR_Cyt2     ---------- ---------- ---------- ---------- ---------- ---------- ---------- ---------- 4
POPTR_Cyt1     ---------- ---------- ---------- ---------- ---------- ---------- ---------- ---------- 4
RICCO_Cyt      ---------- ---------- ---------- ---------- ---------- ---------- ---------- ---------- 4
GOSHI_Cyt      ---------- ---------- ---------- ---------- ---------- ---------- ---------- ---------- 4
VITVI_Cyt      ---------- ---------- ---------- ---------- ---------- ---------- ---------- ---------- 4
SOLTU_Cyt      ---------- ---------- ---------- ---------- ---------- ---------- ---------- ---------- 4
NICTA_Cyt      ---------- ---------- ---------- ---------- ---------- ---------- ---------- ---------- 4
HELAN_Cyt      ---------- ---------- ---------- ---------- ---------- ---------- ---------- ---------- 4
ARATH_Cyt      ---------- ---------- ---------- ---------- ---------- ---------- ---------- ---------- 4
PISSA_Cyt      ---------- ---------- ---------- ---------- ---------- ---------- ---------- ---------- 4
SORBI_Cyt1     ---------- ---------- ---------- ---------- ---------- ---------- ---------- ---------- 4
ZmPGK3_Cyt     ---------- ---------- ---------- ---------- ---------- ---------- ---------- ---------- 4
ORYSA_IG_Cyt   ---------- ---------- ---------- ---------- ---------- ---------- ---------- ---------- 4
ORYSA_JG_Cyt   ---------- ---------- ---------- ---------- ---------- ---------- ---------- ---------- 4
SORBI_Cyt2     ---------- ---------- ---------- ---------- ---------- ---------- ---------- ---------- 4
ORYSA_JG_Cyt2  ---------- ---------- ---------- ---------- ---------- ---------- ---------- ---------- 4
TRIAE_Cyt      ---------- ---------- ---------- ---------- ---------- ---------- ---------- ---------- 4
POPNI_Chl      ---------- ---------- ---------- ---------- ---------- ---------- ---------- ---------- 79
POPTR_Chl2     ---------- ---------- ---------- ---------- ---------- ---------- ---------- ---------- 79
POPTR_Chl1     ---------- ---------- ---------- ---------- ---------- ---------- ---------- ---------- 79
RICCO_Chl      ---------- ---------- ---------- ---------- ---------- ---------- ---------- ---------- 77
NICTA_Chl      ---------- ---------- ---------- ---------- ---------- ---------- ---------- ---------- 78
NICBE_Chl      ---------- ---------- ---------- ---------- ---------- ---------- ---------- ---------- 78
SOLTU_Chl      ---------- ---------- ---------- ---------- ---------- ---------- ---------- ---------- 79
MUSAC_Chl      ---------- ---------- ---------- ---------- ---------- ---------- ---------- ---------- 77
VITVI_Chl1     ---------- ---------- ---------- ---------- ---------- ---------- ---------- ---------- 76
VITVI_Chl2     ---------- ---------- ---------- ---------- ---------- ---------- ---------- ---------- 76
ARATH_Chl2     ---------- ---------- ---------- ---------- ---------- ---------- ---------- ---------- 79
ARATH_Chl1     ---------- ---------- ---------- ---------- ---------- ---------- ---------- ---------- 76
SORBI_Chl      ---------- ---------- ---------- ---------- ---------- ---------- ---------- ---------- 81
ZmPGK1_Chl     ---------- ---------- ---------- ---------- ---------- ---------- ---------- ---------- 78
ZmPGK2_Chl     ---------- ---------- ---------- ---------- ---------- ---------- ---------- ---------- 75
TRIAE_Chl      ---------- ---------- ---------- ---------- ---------- ---------- ---------- ---------- 75
SPIOL_Chl      ---------- ---------- ---------- ---------- ---------- ---------- ---------- ---------- 31
MUSAC_Cyt      ---------- ---------- ---------- ---------- ---------- ---------- ---------- ---------- 4
ORYSA_IG_Chl   ---------- ---------- ---------- ---------- ---------- ---------- ---------- ---------- 85
ORYSA_JG_Chl2  ---------- ---------- ---------- ---------- ---------- ---------- ---------- ---------- 118
ORYSA_JG_Chl3  ---------- ---------- ---------- ---------- ---------- ---------- ---------- ---------- 74
PICSI_Chl      ---------- ---------- ---------- ---------- ---------- ---------- ---------- ---------- 105
CHABR          ---------- ---------- ---------- ---------- ---------- ---------- ---------- ---------- 118
CHLRE_Chl      ---------- ---------- ---------- ---------- ---------- ---------- ---------- ---------- 62
VOLCA_Chl      ---------- ---------- ---------- ---------- ---------- ---------- ---------- ---------- 63
ZmPGK4_Chl     ---------- ---------- ---------- ---------- ---------- ---------- ---------- ---------- 86
MICSP_Chl      ---------- ---------- ---------- ---------- ---------- ---------- ---------- ---------- 42
SYNSP          ---------- ---------- ---------- ---------- ---------- ---------- ---------- ---------- 3
ORYSA_JG_Chl1  ---------- ---------- ---------- ---------- ---------- ---------- ---------- ---------- 129
BACME          ---------- ---------- ---------- ---------- ---------- ---------- ---------- ---------- 3
BACST          ---------- ---------- ---------- ---------- ---------- ---------- ---------- ---------- 3
THEMA          ---------- ---------- ---------- ---------- ---------- ---------- ---------- ---------- 2
THEAQ          ---------- ---------- ---------- ---------- ---------- ---------- ---------- ---------- 2
ASPNI          ---------- ---------- ---------- ---------- ---------- ---------- ---------- ---------- 6
PENCI          ---------- ---------- ---------- ---------- ---------- ---------- ---------- ---------- 6
YARLI          ---------- ---------- ---------- ---------- ---------- ---------- ---------- ---------- 6
TRIRE          ---------- ---------- ---------- ---------- ---------- ---------- ---------- ---------- 6
TRIVI          ---------- ---------- ---------- ---------- ---------- ---------- ---------- ---------- 6
NEUCR          ---------- ---------- ---------- ---------- ---------- ---------- ---------- ---------- 6
CANMA          ---------- ---------- ---------- ---------- ---------- ---------- ---------- ---------- 6
KLULA          ---------- ---------- ---------- ---------- ---------- ---------- ---------- ---------- 6
SACCE          ---------- ---------- ---------- ---------- ---------- ---------- ---------- ---------- 6
RHINI_PGK1     ---------- ---------- ---------- ---------- ---------- ---------- ---------- ---------- 6
RHINI_PGK2     ---------- ---------- ---------- ---------- ---------- ---------- ---------- ---------- 6
HOMSA_PGK1     ---------- ---------- ---------- ---------- ---------- ---------- ---------- ---------- 6
MUSMU_PGK1     ---------- ---------- ---------- ---------- ---------- ---------- ---------- ---------- 6
GALGA          ---------- ---------- ---------- ---------- ---------- ---------- ---------- ---------- 6
HOMSA_PGK2     ---------- ---------- ---------- ---------- ---------- ---------- ---------- ---------- 6
MUSMU_PGK2     ---------- ---------- ---------- ---------- ---------- ---------- ---------- ---------- 6
SCHMA          ---------- ---------- ---------- ---------- ---------- ---------- ---------- ---------- 5
DROME          ---------- ---------- ---------- ---------- ---------- ---------- ---------- ---------- 5
PLAFA          ---------- ---------- ---------- ---------- ---------- ---------- ---------- ---------- 5
TETTH          ---------- ---------- ---------- ---------- ---------- ---------- ---------- ---------- 5
TRYBR_Gly      ---------- ---------- ---------- ---------- ---------- ---------- ---------- ---------- 14
TRYCO_Gly      ---------- ---------- ---------- ---------- ---------- ---------- ---------- ---------- 14
TRYCO_Cyt      ---------- ---------- ---------- ---------- ---------- ---------- ---------- ---------- 6
TRYBR_Cyt1     ---------- ---------- ---------- ---------- ---------- ---------- ---------- ---------- 6
TRYBR_Cyt2     ---------- ---------- ---------- ---------- ---------- ---------- ---------- ---------- 6
CRIFA_Cyt      ---------- ---------- ---------- ---------- ---------- ---------- ---------- ---------- 6
CRIFA_Gly      ---------- ---------- ---------- ---------- ---------- ---------- ---------- ---------- 11
CORGL          ---------- ---------- ---------- ---------- ---------- ---------- ---------- ---------- 3
MYCLE          ---------- ---------- ---------- ---------- ---------- ---------- ---------- ---------- 3
XANFL          ---------- ---------- ---------- ---------- ---------- ---------- ---------- ---------- 4
ZYMMO          ---------- ---------- ---------- ---------- ---------- ---------- ---------- ---------- 3
ESCCO          ---------- ---------- ---------- ---------- ---------- ---------- ---------- ---------- 1
HAEIN          ---------- ---------- ---------- ---------- ---------- ---------- ---------- ---------- 1
CHLRE_Cyt      ---------- ---------- ---------- ---------- ---------- ---------- ---------- ---------- 21
VOLCA_Cyt      ---------- ---------- ---------- ---------- ---------- ---------- ---------- ---------- 23
METBR          ---------- ---------- ---------- ---------- ---------- ---------- ---------- ---------- 6
METFE          ---------- ---------- ---------- ---------- ---------- ---------- ---------- ---------- 5
PICSI_Cyt      ---------- ---------- ---------- ---------- ---------- ---------- ---------- ---------- 28
MICSP_Cyt      ---------- ---------- ---------- ---------- ---------- ---------- ---------- ---------- 9
HALVA          ---------- ---------- ---------- ---------- ---------- ---------- ---------- ---------- 4
```

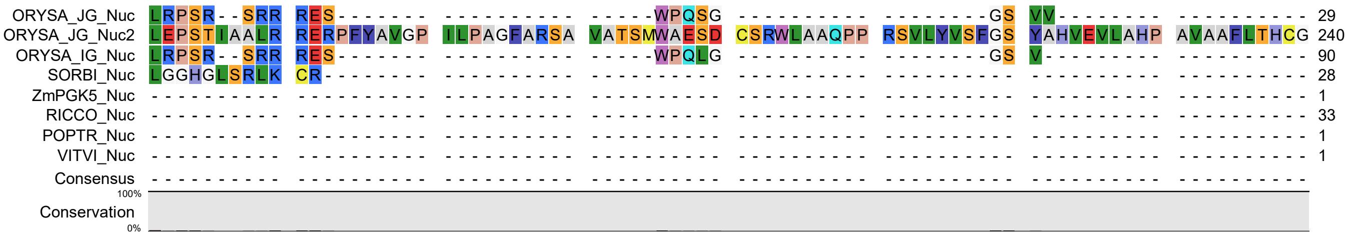
ORYSA_JG_Nuc LRPSR--SRR RES------- ---------- -----WPQSG ---------- --------GS VV-------- ---------- 29
ORYSA_JG_Nuc2 LEPSTIAALR RERPFYAVGP ILPAGFARSA VATSMWAESD CSRWLAAQPP RSVLYVSFGS YAHVEVLAHP AVAAFLTHCG 240
ORYSA_IG_Nuc LRPSR--SRR RES------- ---------- -----WPQLG ---------- --------GS V--------- ---------- 90
SORBI_Nuc LGGHGLSRLK CR-------- ---------- ---------- ---------- ---------- ---------- ---------- 28
ZmPGK5_Nuc ---------- ---------- ---------- ---------- ---------- ---------- ---------- ---------- 1
RICCO_Nuc ---------- ---------- ---------- ---------- ---------- ---------- ---------- ---------- 33
POPTR_Nuc ---------- ---------- ---------- ---------- ---------- ---------- ---------- ---------- 1
VITVI_Nuc ---------- ---------- ---------- ---------- ---------- ---------- ---------- ---------- 1
Consensus ---------- ---------- ---------- ---------- ---------- ---------- ---------- ----------
100%
Conservation
0%

	260	280	300	320	
POPNI_Cyt	---------- ----------	---------- ----------	---------- ----------	---------- ----------	4
POPTR_Cyt2	---------- ----------	---------- ----------	---------- ----------	---------- ----------	4
POPTR_Cyt1	---------- ----------	---------- ----------	---------- ----------	---------- ----------	4
RICCO_Cyt	---------- ----------	---------- ----------	---------- ----------	---------- ----------	4
GOSHI_Cyt	---------- ----------	---------- ----------	---------- ----------	---------- ----------	4
VITVI_Cyt	---------- ----------	---------- ----------	---------- ----------	---------- ----------	4
SOLTU_Cyt	---------- ----------	---------- ----------	---------- ----------	---------- ----------	4
NICTA_Cyt	---------- ----------	---------- ----------	---------- ----------	---------- ----------	4
HELAN_Cyt	---------- ----------	---------- ----------	---------- ----------	---------- ----------	4
ARATH_Cyt	---------- ----------	---------- ----------	---------- ----------	---------- ----------	4
PISSA_Cyt	---------- ----------	---------- ----------	---------- ----------	---------- ----------	4
SORBI_Cyt1	---------- ----------	---------- ----------	---------- ----------	---------- ----------	4
ZmPGK3_Cyt	---------- ----------	---------- ----------	---------- ----------	---------- ----------	4
ORYSA_IG_Cyt	---------- ----------	---------- ----------	---------- ----------	---------- ----------	4
ORYSA_JG_Cyt	---------- ----------	---------- ----------	---------- ----------	---------- ----------	4
SORBI_Cyt2	---------- ----------	---------- ----------	---------- ----------	---------- ----------	4
ORYSA_JG_Cyt2	---------- ----------	---------- ----------	---------- ----------	---------- ----------	4
TRIAE_Cyt	---------- ----------	---------- ----------	---------- ----------	---------- ----------	4
POPNI_Chl	---------- ----------	---------- ----------	---------- ----------	---------- ----------	79
POPTR_Chl2	---------- ----------	---------- ----------	---------- ----------	---------- ----------	79
POPTR_Chl1	---------- ----------	---------- ----------	---------- ----------	---------- ----------	79
RICCO_Chl	---------- ----------	---------- ----------	---------- ----------	---------- ----------	77
NICTA_Chl	---------- ----------	---------- ----------	---------- ----------	---------- ----------	78
NICBE_Chl	---------- ----------	---------- ----------	---------- ----------	---------- ----------	78
SOLTU_Chl	---------- ----------	---------- ----------	---------- ----------	---------- ----------	79
MUSAC_Chl	---------- ----------	---------- ----------	---------- ----------	---------- ----------	77
VITVI_Chl1	---------- ----------	---------- ----------	---------- ----------	---------- ----------	76
VITVI_Chl2	---------- ----------	---------- ----------	---------- ----------	---------- ----------	76
ARATH_Chl2	---------- ----------	---------- ----------	---------- ----------	---------- ----------	79
ARATH_Chl1	---------- ----------	---------- ----------	---------- ----------	---------- ----------	76
SORBI_Chl	---------- ----------	---------- ----------	---------- ----------	---------- ----------	81
ZmPGK1_Chl	---------- ----------	---------- ----------	---------- ----------	---------- ----------	78
ZmPGK2_Chl	---------- ----------	---------- ----------	---------- ----------	---------- ----------	75
TRIAE_Chl	---------- ----------	---------- ----------	---------- ----------	---------- ----------	75
SPIOL_Chl	---------- ----------	---------- ----------	---------- ----------	---------- ----------	31
MUSAC_Cyt	---------- ----------	---------- ----------	---------- ----------	---------- ----------	4
ORYSA_IG_Chl	---------- ----------	---------- ----------	---------- ----------	---------- ----------	85
ORYSA_JG_Chl2	---------- ----------	---------- ----------	---------- ----------	---------- ----------	118
ORYSA_JG_Chl3	---------- ----------	---------- ----------	---------- ----------	---------- ----------	74
PICSI_Chl	---------- ----------	---------- ----------	---------- ----------	---------- ----------	105
CHABR	---------- ----------	---------- ----------	---------- ----------	---------- ----------	118
CHLRE_Chl	---------- ----------	---------- ----------	---------- ----------	---------- ----------	62
VOLCA_Chl	---------- ----------	---------- ----------	---------- ----------	---------- ----------	63
ZmPGK4_Chl	---------- ----------	---------- ----------	---------- ----------	---------- ----------	86
MICSP_Chl	---------- ----------	---------- ----------	---------- ----------	---------- ----------	42
SYNSP	---------- ----------	---------- ----------	---------- ----------	---------- ----------	3
ORYSA_JG_Chl1	---------- ----------	---------- ----------	---------- ----------	---------- ----------	129
BACME	---------- ----------	---------- ----------	---------- ----------	---------- ----------	3
BACST	---------- ----------	---------- ----------	---------- ----------	---------- ----------	3
THEMA	---------- ----------	---------- ----------	---------- ----------	---------- ----------	2
THEAQ	---------- ----------	---------- ----------	---------- ----------	---------- ----------	2
ASPNI	---------- ----------	---------- ----------	---------- ----------	---------- ----------	6
PENCI	---------- ----------	---------- ----------	---------- ----------	---------- ----------	6
YARLI	---------- ----------	---------- ----------	---------- ----------	---------- ----------	6
TRIRE	---------- ----------	---------- ----------	---------- ----------	---------- ----------	6
TRIVI	---------- ----------	---------- ----------	---------- ----------	---------- ----------	6
NEUCR	---------- ----------	---------- ----------	---------- ----------	---------- ----------	6
CANMA	---------- ----------	---------- ----------	---------- ----------	---------- ----------	6
KLULA	---------- ----------	---------- ----------	---------- ----------	---------- ----------	6
SACCE	---------- ----------	---------- ----------	---------- ----------	---------- ----------	6
RHINI_PGK1	---------- ----------	---------- ----------	---------- ----------	---------- ----------	6
RHINI_PGK2	---------- ----------	---------- ----------	---------- ----------	---------- ----------	6
HOMSA_PGK1	---------- ----------	---------- ----------	---------- ----------	---------- ----------	6
MUSMU_PGK1	---------- ----------	---------- ----------	---------- ----------	---------- ----------	6
GALGA	---------- ----------	---------- ----------	---------- ----------	---------- ----------	6
HOMSA_PGK2	---------- ----------	---------- ----------	---------- ----------	---------- ----------	6
MUSMU_PGK2	---------- ----------	---------- ----------	---------- ----------	---------- ----------	6
SCHMA	---------- ----------	---------- ----------	---------- ----------	---------- ----------	5
DROME	---------- ----------	---------- ----------	---------- ----------	---------- ----------	5
PLAFA	---------- ----------	---------- ----------	---------- ----------	---------- ----------	5
TETTH	---------- ----------	---------- ----------	---------- ----------	---------- ----------	5
TRYBR_Gly	---------- ----------	---------- ----------	---------- ----------	---------- ----------	14
TRYCO_Gly	---------- ----------	---------- ----------	---------- ----------	---------- ----------	14
TRYCO_Cyt	---------- ----------	---------- ----------	---------- ----------	---------- ----------	6
TRYBR_Cyt1	---------- ----------	---------- ----------	---------- ----------	---------- ----------	6
TRYBR_Cyt2	---------- ----------	---------- ----------	---------- ----------	---------- ----------	6
CRIFA_Cyt	---------- ----------	---------- ----------	---------- ----------	---------- ----------	6
CRIFA_Gly	---------- ----------	---------- ----------	---------- ----------	---------- ----------	11
CORGL	---------- ----------	---------- ----------	---------- ----------	---------- ----------	3
MYCLE	---------- ----------	---------- ----------	---------- ----------	---------- ----------	3
XANFL	---------- ----------	---------- ----------	---------- ----------	---------- ----------	4
ZYMMO	---------- ----------	---------- ----------	---------- ----------	---------- ----------	3
ESCCO	---------- ----------	---------- ----------	---------- ----------	---------- ----------	1
HAEIN	---------- ----------	---------- ----------	---------- ----------	---------- ----------	1
CHLRE_Cyt	---------- ----------	---------- ----------	---------- ----------	---------- ----------	21
VOLCA_Cyt	---------- ----------	---------- ----------	---------- ----------	---------- ----------	23
METBR	---------- ----------	---------- ----------	---------- ----------	---------- ----------	6
METFE	---------- ----------	---------- ----------	---------- ----------	---------- ----------	5
PICSI_Cyt	---------- ----------	---------- ----------	---------- ----------	---------- ----------	28
MICSP_Cyt	---------- ----------	---------- ----------	---------- ----------	---------- ----------	9
HALVA	---------- ----------	---------- ----------	---------- ----------	---------- ----------	4

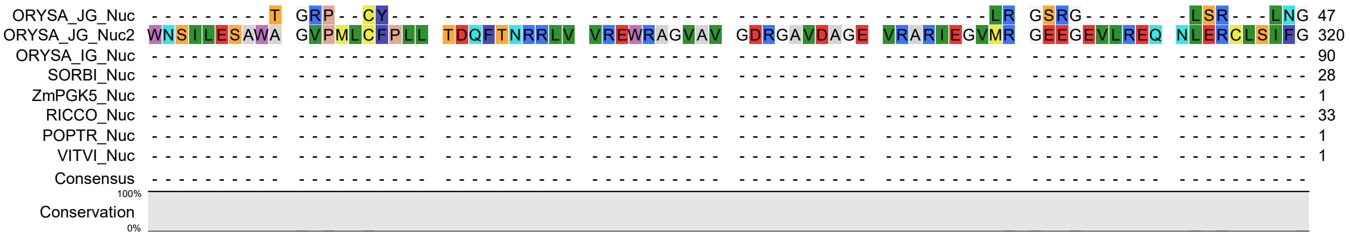
ORYSA_JG_Nuc ---------T GRP--CY--- ---------- ---------- ---------- --------LR GSRG------ -LSR---LNG 47
ORYSA_JG_Nuc2 WNSILESAWA GVPMLCFPLL TDQFTNRRLV VREWRAGVAV GDRGAVDAGE VRARIEGVMR GEEGEVLREQ NLERCLSIFG 320
ORYSA_IG_Nuc ---------- ---------- ---------- ---------- ---------- ---------- ---------- ---------- 90
SORBI_Nuc ---------- ---------- ---------- ---------- ---------- ---------- ---------- ---------- 28
ZmPGK5_Nuc ---------- ---------- ---------- ---------- ---------- ---------- ---------- ---------- 1
RICCO_Nuc ---------- ---------- ---------- ---------- ---------- ---------- ---------- ---------- 33
POPTR_Nuc ---------- ---------- ---------- ---------- ---------- ---------- ---------- ---------- 1
VITVI_Nuc ---------- ---------- ---------- ---------- ---------- ---------- ---------- ---------- 1
Consensus ---------- ---------- ---------- ---------- ---------- ---------- ---------- ----------
100%
Conservation
0%

```
                 340                   360                   380                   400
                   |                     |                     |                     |
POPNI_Cyt      ---------- ---------- ---------- ---------- ---------- ---------- ---------- ---------- 4
POPTR_Cyt2     ---------- ---------- ---------- ---------- ---------- ---------- ---------- ---------- 4
POPTR_Cyt1     ---------- ---------- ---------- ---------- ---------- ---------- ---------- ---------- 4
RICCO_Cyt      ---------- ---------- ---------- ---------- ---------- ---------- ---------- ---------- 4
GOSHI_Cyt      ---------- ---------- ---------- ---------- ---------- ---------- ---------- ---------- 4
VITVI_Cyt      ---------- ---------- ---------- ---------- ---------- ---------- ---------- ---------- 4
SOLTU_Cyt      ---------- ---------- ---------- ---------- ---------- ---------- ---------- ---------- 4
NICTA_Cyt      ---------- ---------- ---------- ---------- ---------- ---------- ---------- ---------- 4
HELAN_Cyt      ---------- ---------- ---------- ---------- ---------- ---------- ---------- ---------- 4
ARATH_Cyt      ---------- ---------- ---------- ---------- ---------- ---------- ---------- ---------- 4
PISSA_Cyt      ---------- ---------- ---------- ---------- ---------- ---------- ---------- ---------- 4
SORBI_Cyt1     ---------- ---------- ---------- ---------- ---------- ---------- ---------- ---------- 4
ZmPGK3_Cyt     ---------- ---------- ---------- ---------- ---------- ---------- ---------- ---------- 4
ORYSA_IG_Cyt   ---------- ---------- ---------- ---------- ---------- ---------- ---------- ---------- 4
ORYSA_JG_Cyt   ---------- ---------- ---------- ---------- ---------- ---------- ---------- ---------- 4
SORBI_Cyt2     ---------- ---------- ---------- ---------- ---------- ---------- ---------- ---------- 4
ORYSA_JG_Cyt2  ---------- ---------- ---------- ---------- ---------- ---------- ---------- ---------- 4
TRIAE_Cyt      ---------- ---------- ---------- ---------- ---------- ---------- ---------- ---------- 4
POPNI_Chl      ---------- ---------- ---------- ---------- ---------- ---------- ---------- ---------- 79
POPTR_Chl2     ---------- ---------- ---------- ---------- ---------- ---------- ---------- ---------- 79
POPTR_Chl1     ---------- ---------- ---------- ---------- ---------- ---------- ---------- ---------- 79
RICCO_Chl      ---------- ---------- ---------- ---------- ---------- ---------- ---------- ---------- 77
NICTA_Chl      ---------- ---------- ---------- ---------- ---------- ---------- ---------- ---------- 78
NICBE_Chl      ---------- ---------- ---------- ---------- ---------- ---------- ---------- ---------- 78
SOLTU_Chl      ---------- ---------- ---------- ---------- ---------- ---------- ---------- ---------- 79
MUSAC_Chl      ---------- ---------- ---------- ---------- ---------- ---------- ---------- ---------- 77
VITVI_Chl1     ---------- ---------- ---------- ---------- ---------- ---------- ---------- ---------- 76
VITVI_Chl2     ---------- ---------- ---------- ---------- ---------- ---------- ---------- ---------- 76
ARATH_Chl2     ---------- ---------- ---------- ---------- ---------- ---------- ---------- ---------- 79
ARATH_Chl1     ---------- ---------- ---------- ---------- ---------- ---------- ---------- ---------- 76
SORBI_Chl      ---------- ---------- ---------- ---------- ---------- ---------- ---------- ---------- 81
ZmPGK1_Chl     ---------- ---------- ---------- ---------- ---------- ---------- ---------- ---------- 78
ZmPGK2_Chl     ---------- ---------- ---------- ---------- ---------- ---------- ---------- ---------- 75
TRIAE_Chl      ---------- ---------- ---------- ---------- ---------- ---------- ---------- ---------- 75
SPIOL_Chl      ---------- ---------- ---------- ---------- ---------- ---------- ---------- ---------- 31
MUSAC_Cyt      ---------- ---------- ---------- ---------- ---------- ---------- ---------- ---------- 4
ORYSA_IG_Chl   ---------- ---------- ---------- ---------- ---------- ---------- ---------- ---------- 85
ORYSA_JG_Chl2  ---------- ---------- ---------- ---------- ---------- ---------- ---------- ---------- 118
ORYSA_JG_Chl3  ---------- ---------- ---------- ---------- ---------- ---------- ---------- ---------- 74
PICSI_Chl      ---------- ---------- ---------- ---------- ---------- ---------- ---------- ---------- 105
CHABR          ---------- ---------- ---------- ---------- ---------- ---------- ---------- ---------- 118
CHLRE_Chl      ---------- ---------- ---------- ---------- ---------- ---------- ---------- ---------- 62
VOLCA_Chl      ---------- ---------- ---------- ---------- ---------- ---------- ---------- ---------- 63
ZmPGK4_Chl     ---------- ---------- ---------- ---------- ---------- ---------- ---------- ---------- 86
MICSP_Chl      ---------- ---------- ---------- ---------- ---------- ---------- ---------- ---------- 42
SYNSP          ---------- ---------- ---------- ---------- ---------- ---------- ---------- ---------- 3
ORYSA_JG_Chl1  ---------- ---------- ---------- ---------- ---------- ---------- ---------- ---------- 129
BACME          ---------- ---------- ---------- ---------- ---------- ---------- ---------- ---------- 3
BACST          ---------- ---------- ---------- ---------- ---------- ---------- ---------- ---------- 3
THEMA          ---------- ---------- ---------- ---------- ---------- ---------- ---------- ---------- 2
THEAQ          ---------- ---------- ---------- ---------- ---------- ---------- ---------- ---------- 2
ASPNI          ---------- ---------- ---------- ---------- ---------- ---------- ---------- ---------- 6
PENCI          ---------- ---------- ---------- ---------- ---------- ---------- ---------- ---------- 6
YARLI          ---------- ---------- ---------- ---------- ---------- ---------- ---------- ---------- 6
TRIRE          ---------- ---------- ---------- ---------- ---------- ---------- ---------- ---------- 6
TRIVI          ---------- ---------- ---------- ---------- ---------- ---------- ---------- ---------- 6
NEUCR          ---------- ---------- ---------- ---------- ---------- ---------- ---------- ---------- 6
CANMA          ---------- ---------- ---------- ---------- ---------- ---------- ---------- ---------- 6
KLULA          ---------- ---------- ---------- ---------- ---------- ---------- ---------- ---------- 6
SACCE          ---------- ---------- ---------- ---------- ---------- ---------- ---------- ---------- 6
RHINI_PGK1     ---------- ---------- ---------- ---------- ---------- ---------- ---------- ---------- 6
RHINI_PGK2     ---------- ---------- ---------- ---------- ---------- ---------- ---------- ---------- 6
HOMSA_PGK1     ---------- ---------- ---------- ---------- ---------- ---------- ---------- ---------- 6
MUSMU_PGK1     ---------- ---------- ---------- ---------- ---------- ---------- ---------- ---------- 6
GALGA          ---------- ---------- ---------- ---------- ---------- ---------- ---------- ---------- 6
HOMSA_PGK2     ---------- ---------- ---------- ---------- ---------- ---------- ---------- ---------- 6
MUSMU_PGK2     ---------- ---------- ---------- ---------- ---------- ---------- ---------- ---------- 6
SCHMA          ---------- ---------- ---------- ---------- ---------- ---------- ---------- ---------- 5
DROME          ---------- ---------- ---------- ---------- ---------- ---------- ---------- ---------- 5
PLAFA          ---------- ---------- ---------- ---------- ---------- ---------- ---------- ---------- 5
TETTH          ---------- ---------- ---------- ---------- ---------- ---------- ---------- ---------- 5
TRYBR_Gly      ---------- ---------- ---------- ---------- ---------- ---------- ---------- ---------- 14
TRYCO_Gly      ---------- ---------- ---------- ---------- ---------- ---------- ---------- ---------- 14
TRYCO_Cyt      ---------- ---------- ---------- ---------- ---------- ---------- ---------- ---------- 6
TRYBR_Cyt1     ---------- ---------- ---------- ---------- ---------- ---------- ---------- ---------- 6
TRYBR_Cyt2     ---------- ---------- ---------- ---------- ---------- ---------- ---------- ---------- 6
CRIFA_Cyt      ---------- ---------- ---------- ---------- ---------- ---------- ---------- ---------- 6
CRIFA_Gly      ---------- ---------- ---------- ---------- ---------- ---------- ---------- ---------- 11
CORGL          ---------- ---------- ---------- ---------- ---------- ---------- ---------- ---------- 3
MYCLE          ---------- ---------- ---------- ---------- ---------- ---------- ---------- ---------- 3
XANFL          ---------- ---------- ---------- ---------- ---------- ---------- ---------- ---------- 4
ZYMMO          ---------- ---------- ---------- ---------- ---------- ---------- ---------- ---------- 3
ESCCO          ---------- ---------- ---------- ---------- ---------- ---------- ---------- ---------- 1
HAEIN          ---------- ---------- ---------- ---------- ---------- ---------- ---------- ---------- 1
CHLRE_Cyt      ---------- ---------- ---------- ---------- ---------- ---------- ---------- ---------- 21
VOLCA_Cyt      ---------- ---------- ---------- ---------- ---------- ---------- ---------- ---------- 23
METBR          ---------- ---------- ---------- ---------- ---------- ---------- ---------- ---------- 6
METFE          ---------- ---------- ---------- ---------- ---------- ---------- ---------- ---------- 5
PICSI_Cyt      ---------- ---------- ---------- ---------- ---------- ---------- ---------- ---------- 28
MICSP_Cyt      ---------- ---------- ---------- ---------- ---------- ---------- ---------- ---------- 9
HALVA          ---------- ---------- ---------- ---------- ---------- ---------- ---------- ---------- 4
```

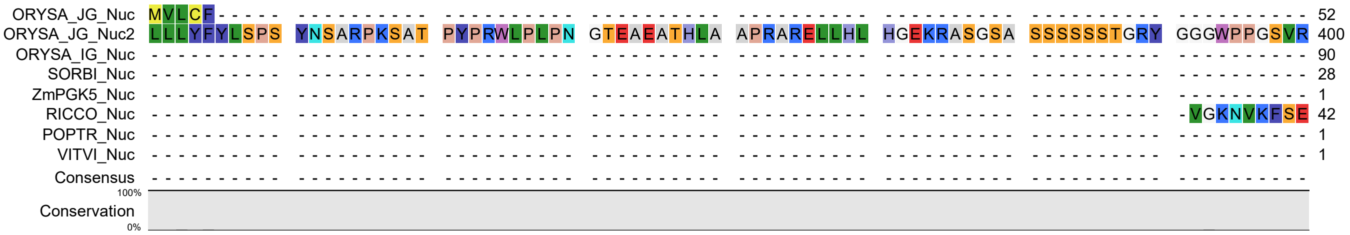

ORYSA_JG_Nuc MVLCF----- ---------- ---------- ---------- ---------- ---------- ---------- ---------- 52
ORYSA_JG_Nuc2 LLLYFYLSPS YNSARPKSAT PYPRWLPLPN GTEAEATHLA APRARELLHL HGEKRASGSA SSSSSSTGRY GGGWPPGSVR 400
ORYSA_IG_Nuc ---------- ---------- ---------- ---------- ---------- ---------- ---------- ---------- 90
SORBI_Nuc ---------- ---------- ---------- ---------- ---------- ---------- ---------- ---------- 28
ZmPGK5_Nuc ---------- ---------- ---------- ---------- ---------- ---------- ---------- ---------- 1
RICCO_Nuc ---------- ---------- ---------- ---------- ---------- ---------- ---------- -VGKNVKFSE 42
POPTR_Nuc ---------- ---------- ---------- ---------- ---------- ---------- ---------- ---------- 1
VITVI_Nuc ---------- ---------- ---------- ---------- ---------- ---------- ---------- ---------- 1
Consensus ---------- ---------- ---------- ---------- ---------- ---------- ---------- ----------
100%
Conservation
0%

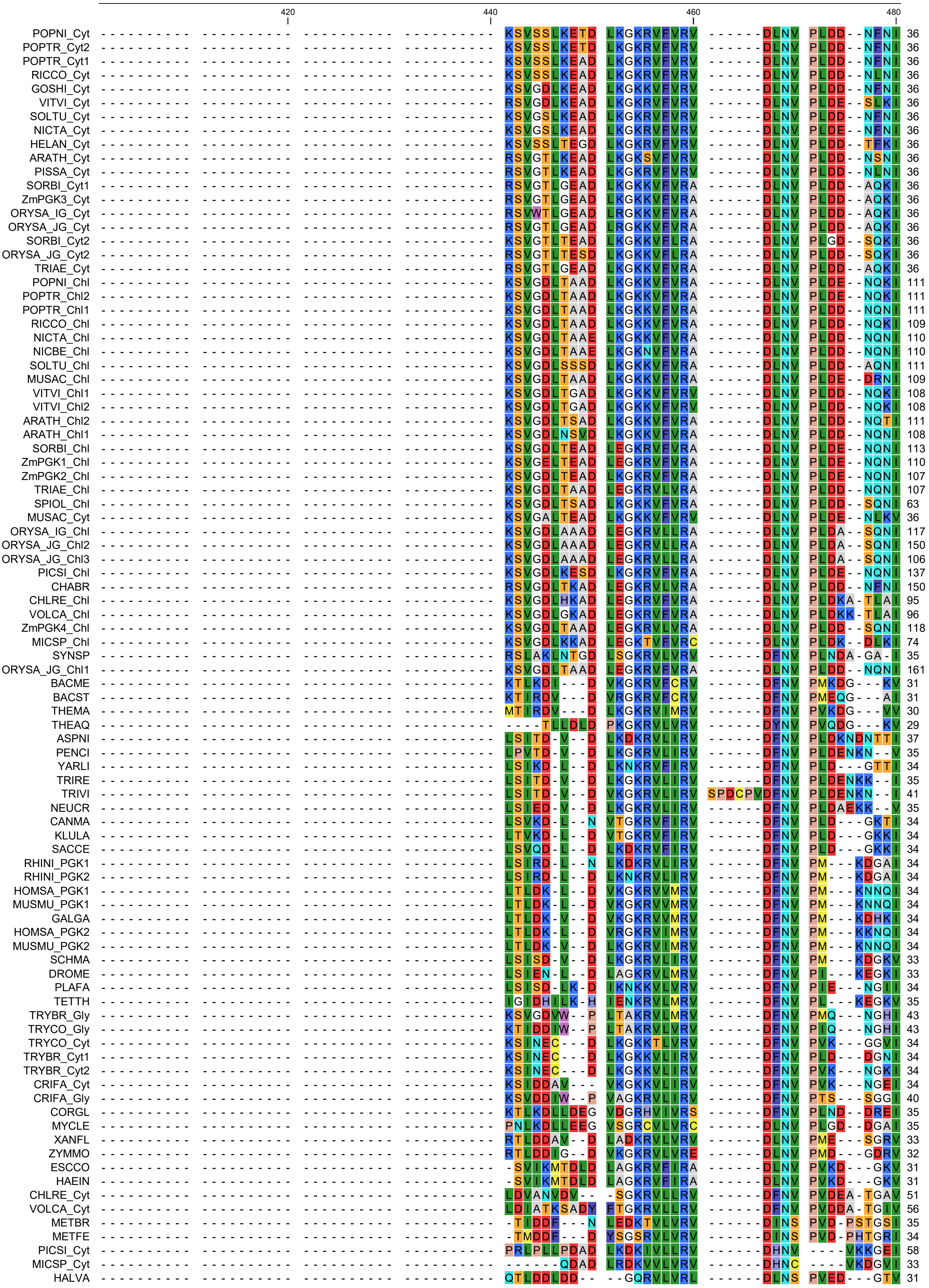
420 440 460 480
POPNI_Cyt KSVSSLKETD LKGKRVFVRV ------DLNV PLDD--NFNI 36
POPTR_Cyt2 KSVSSLKETD LKGKRVFVRV ------DLNV PLDD--NFNI 36
POPTR_Cyt1 KSVSSLKEAD LKGKRVFVRV ------DLNV PLDD--NFNI 36
RICCO_Cyt KSVSSLKEAD LKGKRVFVRV ------DLNV PLDD--NLNI 36
GOSHI_Cyt KSVGDLKEAD LKGKKVFVRV ------DLNV PLDD--NFNI 36
VITVI_Cyt RSVGDLKEAD LKGKRVFVRV ------DLNV PLDE--SLKI 36
SOLTU_Cyt KSVGSLKEAD LKGKRVFVRV ------DLNV PLDD--NFNI 36
NICTA_Cyt KSVGSLKEAD LKGKRVFVRV ------DLNV PLDE--NFNI 36
HELAN_Cyt KSVSSLTEGD LKGKRVFVRV ------DLNV PLDD--TFKI 36
ARATH_Cyt RSVGTLKEAD LKGKSVFVRV ------DLNV PLDD--NSNI 36
PISSA_Cyt RSVGTLKEAD LKGKRVFVRV ------DLNV PLDD--NLNI 36
SORBI_Cyt1 RSVGTLGEAD LKGKKVFVRA ------DLNV PLDD--AQKI 36
ZmPGK3_Cyt RSVGTLGEAD LKGKKVFVRA ------DLNV PLDD--AQKI 36
ORYSA_IG_Cyt RSVWTLGEAD LRGKKVFVRA ------DLNV PLDD--AQKI 36
ORYSA_JG_Cyt RSVGTLGEAD LRGKKVFVRA ------DLNV PLDD--AQKI 36
SORBI_Cyt2 KSVGTLTEAD LKGKKVFLRA ------DLNV PLGD--SQKI 36
ORYSA_JG_Cyt2 RSVGTLTESD LKGKKVFLRA ------DLNV PLDD--SQKI 36
TRIAE_Cyt RSVGTLGEAD LKGKKVFVRA ------DLNV PLDD--AQKI 36
POPNI_Chl KSVGDLTAAD LKGKKVFVRA ------DLNV PLDE--NQKI 111
POPTR_Chl2 KSVGDLTAAD LKGKKVFVRA ------DLNV PLDE--NQKI 111
POPTR_Chl1 KSVGDLTAAD LKGKKVFVRA ------DLNV PLDD--NQNI 111
RICCO_Chl KSVGDLTAAD LKGKKVFVRA ------DLNV PLDD--NQKI 109
NICTA_Chl KSVGDLTAAE LKGKKVFVRA ------DLNV PLDD--NQNI 110
NICBE_Chl KSVGDLTAAE LKGKNVFVRA ------DLNV PLDD--NQNI 110
SOLTU_Chl KSVGDLSSSD LKGKKVFVRA ------DLNV PLDD--AQNI 111
MUSAC_Chl KSVGDLTAAD LKGKKVFVRA ------DLNV PLDE--DRNI 109
VITVI_Chl1 KSVGDLTGAD LKGKKVFVRV ------DLNV PLDD--NQKI 108
VITVI_Chl2 KSVGDLTGAD LKGKKVFVRV ------DLNV PLDD--NQKI 108
ARATH_Chl2 KSVGDLTSAD LKGKKVFVRA ------DLNV PLDD--NQTI 111
ARATH_Chl1 KSVGDLNSVD LKGKKVFVRA ------DLNV PLDD--NQNI 108
SORBI_Chl KSVGDLTEAD LEGKRVFVRA ------DLNV PLDE--NQNI 113
ZmPGK1_Chl KSVGELTEAD LEGKRVFVRA ------DLNV PLDE--NQNI 110
ZmPGK2_Chl KSVGDLTEAD LKGKRVFVRA ------DLNV PLDE--NQNI 107
TRIAE_Chl KSVGDLTAAD LEGKRVLVRA ------DLNV PLDD--NQNI 107
SPIOL_Chl KSVGDLTSAD LKGKKVFVRA ------DLNV PLDD--SQNI 63
MUSAC_Cyt KSVGALTEAD LKGKKVFVRV ------DLNV PLDE--NLKV 36
ORYSA_IG_Chl KSVGDLAAAD LEGKRVLLRA ------DLNV PLDA--SQNI 117
ORYSA_JG_Chl2 KSVGDLAAAD LEGKRVLLRA ------DLNV PLDA--SQNI 150
ORYSA_JG_Chl3 KSVGDLAAAD LEGKRVLLRA ------DLNV PLDA--SQNI 106
PICSI_Chl KSVGDLKESD LKGKRVFVRA ------DLNV PLDE--NQNI 137
CHABR RSVGDLTKAD LEGKRVLVRA ------DLNV PLDD--NFNI 150
CHLRE_Chl KSVGDLHKAD LEGKRVFVRA ------DLNV PLDKA-TLAI 95
VOLCA_Chl KSVGDLGKAD LEGKRVFVRA ------DLNV PLDKK-TLAI 96
ZmPGK4_Chl KSVGDLTAAD LEGKRVLVRA ------DLNV PLDD--SQNI 118
MICSP_Chl KSVGDLKKAD LEGKTVFVRC ------DLNV PLDK--DLKI 74
SYNSP RSLAKLNTGD LSGKRVLVRV ------DFNV PLNDA-GA-I 35
ORYSA_JG_Chl1 KSVGDLTAAD LEGKRVFVRA ------DLNV PLDD--NQNI 161
BACME KTLKDI---D VKGKRVFCRV ------DFNV PMKDG---KV 31
BACST KTIRDV---D VRGKRVFCRV ------DFNV PMEQG---AI 31
THEMA MTIRDV---D LKGKRVIMRV ------DFNV PVKDG---VV 30
THEAQ ----TLLDLD PKGKRVLVRV ------DYNV PVQDG---KV 29
ASPNI LSITD-V--D LKDKRVLIRV ------DFNV PLDKNDNTTI 37
PENCI LPVTD-V--D LKGKRVLIRV ------DFNV PLDENKN--V 35
YARLI LSIKD-L--D LKNKRVFIRV ------DFNV PLD---GTTI 34
TRIRE LSITD-V--D LKGKRVLIRV ------DFNV PLDENKK--I 35
TRIVI LSITD-V--D VKGKRVLIRV SPDCPVDFNV PLDENKN--I 41
NEUCR LSIED-V--D LKGKRVLIRV ------DFNV PLDAEKK--V 35
CANMA LSVKD-L--N VTGKRVFIRV ------DFNV PLD---GKTI 34
KLULA LTVKD-L--D VTGKRVFIRV ------DFNV PLD---GKKI 34
SACCE LSVQD-L--D LKDKRVFIRV ------DFNV PLD---GKKI 34
RHINI_PGK1 LSIRD-L--N LKDKRVLIRV ------DFNV PM---KDGAI 34
RHINI_PGK2 LSIRD-L--D LKNKRVLIRV ------DFNV PM---KDGAI 34
HOMSA_PGK1 LTLDK-L--D VKGKRVVMRV ------DFNV PM---KNNQI 34
MUSMU_PGK1 LTLDK-L--D VKGKRVVMRV ------DFNV PM---KNNQI 34
GALGA LTLDK-V--D VKGKRVVMRV ------DFNV PM---KDHKI 34
HOMSA_PGK2 LTLDK-L--D VRGKRVIMRV ------DFNV PM---KKNQI 34
MUSMU_PGK2 LTLDK-L--D LKGKRVIMRV ------DFNV PM---KNNQI 34
SCHMA LSISD-V--D LKGKRVLIRV ------DFNV PM---KDGKV 33
DROME LSIEN-L--D LAGKRVLMRV ------DFNV PI---KEGKI 33
PLAFA LSISD-LK-D IKNKKVLVRV ------DFNV PIE---NGII 34
TETTH IGIDHILK-H IENKRVLMRV ------DFNV PL---KEGKV 35
TRYBR_Gly KSVGDVW--P LTAKRVLMRV ------DFNV PMQ---NGHI 43
TRYCO_Gly KTIDDIW--P LTAKRVLVRV ------DFNV PIQ---NGHI 43
TRYCO_Cyt KSINEC---D LKGKKTLVRV ------DFNV PVK---GGVI 34
TRYBR_Cyt1 KSINEC---D LKGKKVLIRV ------DFNV PLD---DGNI 34
TRYBR_Cyt2 KSINEC---D LKGKKVLIRV ------DFNV PVK---NGKI 34
CRIFA_Cyt KSIDDAV--- VKGKKVLIRV ------DFNV PVK---NGEI 34
CRIFA_Gly KSVDDIW--P VAGKRVLIRV ------DFNV PTS---SGGI 40
CORGL KTLKDLLDEG VDGRHVIVRS ------DFNV PLND--DREI 35
MYCLE PNLKDLLEEG VSGRCVLVRC ------DLNV PLGD--DGAI 35
XANFL RTLDDAV--D LADKRVLVRV ------DLNV PME---SGRV 33
ZYMMO RTLDDIG--D VKGKRVLVRE ------DLNV PMD---GDRV 32
ESCCO -SVIKMTDLD LAGKRVFIRA ------DLNV PVKD---GKV 31
HAEIN -SVIKMTDLD LAGKRVFIRA ------DLNV PVKD---GKV 31
CHLRE_Cyt LDVANVDV-- -SGKRVLLRV ------DFNV PVDEA-TGAV 51
VOLCA_Cyt LDIATKSADY FTGKRVLLRV ------DFNV PVDDA-TGIV 56
METBR -TIDDF---N LEDKTVLVRV ------DINS PVD-PSTGSI 35
METFE -TMDDF---D YSGSRVLVRV ------DINS PVD-PHTGRI 34
PICSI_Cyt PRLPLLPDAD LKDKIVLLRV ------DHNV ----VKKGEI 58
MICSP_Cyt ------QDAD LRDKVVLVRV ------DHNC ----VKDGVI 33
HALVA QTLDDLDD-- --GQRVLVRL ------DLNS PVED---GTV 31

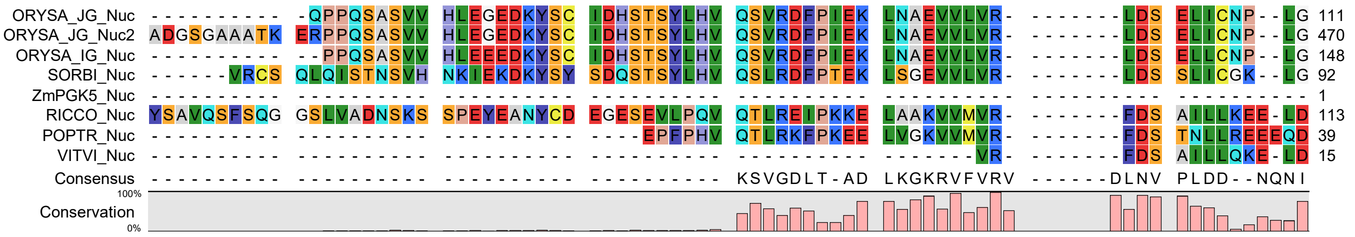
ORYSA_JG_Nuc ---------- -QPPQSASVV HLEGEDKYSC IDHSTSYLHV QSVRDFPIEK LNAEVVLVR- -------LDS ELICNP--LG 111
ORYSA_JG_Nuc2 ADGSGAAATK ERPPQSASVV HLEGEDKYSC IDHSTSYLHV QSVRDFPIEK LNAEVVLVR- -------LDS ELICNP--LG 470
ORYSA_IG_Nuc ---------- --PPQSASVV HLEEEDKYSC IDHSTSYLHV QSVRDFPIEK LNAEVVLVR- -------LDS ELLCNP--LG 148
SORBI_Nuc ------VRCS QLQISTNSVH NKIEKDKYSY SDQSTSYLHV QSLRDFPTEK LSGEVVLVR- -------LDS SLICGK--LG 92
ZmPGK5_Nuc ---------- ---------- ---------- ---------- ---------- ---------- ---------- ---------- 1
RICCO_Nuc YSAVQSFSQG GSLVADNSKS SPEYEANYCD EGESEVLPQV QTLREIPKKE LAAKVVMVR- -------FDS AILLKEE-LD 113
POPTR_Nuc ---------- ---------- ---------- ----EPFPHV QTLRKFPKEE LVGKVVMVR- -------FDS TNLLREEEQD 39
VITVI_Nuc ---------- ---------- ---------- ---------- ---------- --------VR- -------FDS AILLQKE-LD 15
Consensus ---------- ---------- ---------- ---------- KSVGDLT-AD LKGKRVFVRV ------DLNV PLDD--NQNI
100%
Conservation
0%

```
                                   500                   520                   540                   560
POPNI_Cyt      TDDTRIRAAV PTVKYLMDHG AR-VILCSHL GRPKG-VT-- ---------- ---------- ---------- ----------  72
POPTR_Cyt2     TDDTRIRAAV PTIKYLMDHG AR-VILCSHL GRPKG-VT-- ---------- ---------- ---------- ----------  72
POPTR_Cyt1     TDDTRIRAAV PTIKYLMSHG AR-VILSSHL GRPKG-VT-- ---------- ---------- ---------- ----------  72
RICCO_Cyt      TDDTRIRAAV PTIKYLMDHG AR-VILCTHL GRPKG-VT-- ---------- ---------- ---------- ----------  72
GOSHI_Cyt      TDDTRIRAAV PTIKYLMGHG SK-VILSSHL GRPKG-VT-- ---------- ---------- ---------- ----------  72
VITVI_Cyt      TDDTRVRAAV PTIKYLMGHG AK-VILASHL GRPKG-VT-- ---------- ---------- ---------- ----------  72
SOLTU_Cyt      TDDTRIRAAV PTIKYLMQNG AR-VILASHL GRPKGLVH-- ---------- ---------- ---------- ----------  73
NICTA_Cyt      TDDTRIRAAV PTIKYLMQHG SH-VILASHL GRPKG-VT-- ---------- ---------- ---------- ----------  72
HELAN_Cyt      TDDTRIRAAV PTIKYLMSHG AR-VILSSHL GRPKG-VT-- ---------- ---------- ---------- ----------  72
ARATH_Cyt      TDDTRIRAAV PTIKYLMGNG SR-VVLCSHL GRPKG-VT-- ---------- ---------- ---------- ----------  72
PISSA_Cyt      TDDTRIRAAI PTIKYLTGYG AK-VILSSHL GRPKG-VT-- ---------- ---------- ---------- ----------  72
SORBI_Cyt1     TDDTRIRASV PTIKFLLEKG AK-VILASHL GRPKG-VT-- ---------- ---------- ---------- ----------  72
ZmPGK3_Cyt     TDDTRIRASV PTIKFLLEKG AK-VILASHL GRPKG-VT-- ---------- ---------- ---------- ----------  72
ORYSA_IG_Cyt   TDDTRIRASV PTVKFLLEKG AK-VILASHL GRPKG-VT-- ---------- ---------- ---------- ----------  72
ORYSA_JG_Cyt   TDDTRIRASV PTVKFLLEKG AK-VILASHL GRPKG-VT-- ---------- ---------- ---------- ----------  72
SORBI_Cyt2     TDDTRIRASV PTIKFLMEKG AK-VILASHL GRPKG-VT-- ---------- ---------- ---------- ----------  72
ORYSA_JG_Cyt2  TDDTRIRASV PTIKFLMGKG AK-VILASHL GRPKG-VT-- ---------- ---------- ---------- ----------  72
TRIAE_Cyt      TDDTRIRASI PTIKYLLEKG AK-VILASHL GRPKG-VT-- ---------- ---------- ---------- ----------  72
POPNI_Chl      TDDTRIRAAV PTIKYLISKG AK-VILSSHL GRPKG-VT-- ---------- ---------- ---------- ---------- 147
POPTR_Chl2     TDDTRIRAAV PTIKYLISKG AK-VILSSHL GRPKG-VT-- ---------- ---------- ---------- ---------- 147
POPTR_Chl1     TDDTRIRAAI PTIKYLISNG AK-VILSSHL GRPKG-VT-- ---------- ---------- ---------- ---------- 147
RICCO_Chl      TDDTRIRAAI PTVKHLIQNG AK-VILSSHL GRPKG-VT-- ---------- ---------- ---------- ---------- 145
NICTA_Chl      TDDTRIRAAV PTIKHLMANG AK-VILSSHL GRPKG-VT-- ---------- ---------- ---------- ---------- 146
NICBE_Chl      TDDTRIRAAV PTIKHLMANG AK-VILSTHL GRPKG-VT-- ---------- ---------- ---------- ---------- 146
SOLTU_Chl      TDDTRIRAAI PTIKHLIANG AK-VILSTHL GRPKG-VT-- ---------- ---------- ---------- ---------- 147
MUSAC_Chl      TDDTRIRAAV PTIKHLISNG AK-VILSSHL GRPKG-VT-- ---------- ---------- ---------- ---------- 145
VITVI_Chl1     TDDTRVRAAI PTIKYLIDNG AK-VILSSHL GRPKG-VT-- ---------- ---------- ---------- ---------- 144
VITVI_Chl2     TDDTRVRAAI PTIKYLIDNG AK-VILSSHL GRPKG-VT-- ---------- ---------- ---------- ---------- 144
ARATH_Chl2     TDDTRIRAAI PTIKYLIENG AK-VILSTHL GRPKG-VT-- ---------- ---------- ---------- ---------- 147
ARATH_Chl1     TDDTRIRAAI PTIKFLIENG AK-VILSTHL GRPKG-VT-- ---------- ---------- ---------- ---------- 144
SORBI_Chl      TDDTRIRAAV PTIQYLISKG AK-VILSSHL GRPKG-FT-- ---------- ---------- ---------- ---------- 149
ZmPGK1_Chl     TDDTRIRAAI PTIQYILSKG AK-VILSSHL GRPKG-FT-- ---------- ---------- ---------- ---------- 146
ZmPGK2_Chl     TDDTRIRAAI PTIQYLLGKG AK-VILSSHL GRPKG-FT-- ---------- ---------- ---------- ---------- 143
TRIAE_Chl      TDDTRIRAAI PTIKYLLSNG AK-VILTSHL GRPKG-VT-- ---------- ---------- ---------- ---------- 143
SPIOL_Chl      TDDTRIRAAI PTIKHLINNG AK-VILSSHL GRPKG-VT-- ---------- ---------- ---------- ----------  99
MUSAC_Cyt      TDDTRIRASV PTIKYLMERG AR-IILASHL GRPKG-VT-- ---------- ---------- ---------- ----------  72
ORYSA_IG_Chl   TDDTRVRAAI PTIKHLIGNG AK-VILCSHL GRPKG-IT-- ---------- ---------- ---------- ---------- 153
ORYSA_JG_Chl2  TDDTRVIAAI PTIKHLIGNG AK-VILCSHL GRPKG-IT-- ---------- ---------- ---------- ---------- 186
ORYSA_JG_Chl3  TDDTRVIAAI PTIKHLIGNG AK-VILCSHL GRPKG-IT-- ---------- ---------- ---------- ---------- 142
PICSI_Chl      TDDTRIRAAV PTIKYLIGNG AK-VILASHL GRPKG-VT-- ---------- ---------- ---------- ---------- 173
CHABR          TDDTRIRAAV PTVKYLMERG AK-VLLSTHL GRPKG-VT-- ---------- ---------- ---------- ---------- 186
CHLRE_Chl      TDDTRIRAAV PTLKYLLDNG AK-VLLTSHL GRPKGGPE-- ---------- ---------- ---------- ---------- 132
VOLCA_Chl      TDDTRIRAAV PTLKYLLDNG AK-VLLTSHL GRPKGGPE-- ---------- ---------- ---------- ---------- 133
ZmPGK4_Chl     TDDTRVRAAI PTIKHLISNG AR-VILTSHL ---------- ---------- ---------- ---------- ---------- 147
MICSP_Chl      TDDTRIRAAI PTLEYLVENG AK-VLVTSHL GRPKDGPE-- ---------- ---------- ---------- ---------- 111
SYNSP          TDDTRIRAAL PTINDLISKG AK-VILAAHF GRPKGQVN-- ---------- ---------- ---------- ----------  72
ORYSA_JG_Chl1  TDDTRVRAAI PTIQYLIKNG AK-VILSSHL ---------- ---------- ---------- ---------- ---------- 190
BACME          TDETRIRAAI PTIQYLVEQG AK-VILASHL GRPKGEVV-- ---------- ---------- ---------- ----------  68
BACST          TDDTRIRAAI PTIRYLIEHG AK-VILASHL GRPKGKVV-- ---------- ---------- ---------- ----------  68
THEMA          QDDTRIRAAL PTIKYALEQG AK-VILLSHL GRPKGEPS-- ---------- ---------- ---------- ----------  67
THEAQ          QDETRILESL PTLRHLLAGG AS-LVLLSHL GRPKGP-D-- ---------- ---------- ---------- ----------  65
ASPNI          TNPQRIVGAL PTIKYAIDNG AKAVILMSHL GRPDGKKN-- ---------- ---------- ---------- ----------  75
PENCI          TNPQRIVGAL PTIKYAIDNG AKAVVLMSHL GRPDGKVN-- ---------- ---------- ---------- ----------  73
YARLI          TNNQRIVAAL PSIKYAIDQG AKAVILASHL GRPNGQRV-- ---------- ---------- ---------- ----------  72
TRIRE          TNNQRIVGAI PTIKHALDNG AKAVVLMSHL GRPNGAVN-- ---------- ---------- ---------- ----------  73
TRIVI          TNPQRIAGAI PTIKHALDNG AKAVILMSHL GRPNGAVN-- ---------- ---------- ---------- ----------  79
NEUCR          TNPQRIAGAI PTIKYALDHG AKAVVLMSHL GRPDRKPN-- ---------- ---------- ---------- ----------  73
CANMA          TNNQRIVAAL PTIKYVEEQK PKCIILASHL GRPNGERN-- ---------- ---------- ---------- ----------  72
KLULA          TSNQRIVAAL PTIQYVLEKK PKAIVLASHL GRPNGEVN-- ---------- ---------- ---------- ----------  72
SACCE          TSNQRIVAAL PTIKYVLEHH PRYVVLASHL GRPNGERN-- ---------- ---------- ---------- ----------  72
RHINI_PGK1     TNNNRIVQAL PTVKYALDNG ASAVILMSHL GRPNGEAV-- ---------- ---------- ---------- ----------  72
RHINI_PGK2     TNNNRIVQAL PTVKYALDNG ASAVILMSHL GRPNGEAV-- ---------- ---------- ---------- ----------  72
HOMSA_PGK1     TNNQRIKAAV PSIKFCLDNG AKSVVLMSHL GRPDGVPM-- ---------- ---------- ---------- ----------  72
MUSMU_PGK1     TNNQRIKAAV PSIKFCLDNG AKSVVLMSHL GRPDGVPM-- ---------- ---------- ---------- ----------  72
GALGA          TNNQRIKAAV PTIKHCLDHG AKSVVLMSHL GRPDGVPM-- ---------- ---------- ---------- ----------  72
HOMSA_PGK2     TNNQRIKASI PSIKYCLDNG AKAVVLMSHL GRPDGVPM-- ---------- ---------- ---------- ----------  72
MUSMU_PGK2     TNNQRIKAAI PSIKHCLDNG AKSVVLMSHL GRPDGIPM-- ---------- ---------- ---------- ----------  72
SCHMA          TNTQRIAAAI PTIKYALDKG AKSVVLMSHL GRPDGHKV-- ---------- ---------- ---------- ----------  71
DROME          TSNQRIVAAL DSIKLALSKK AKSVVLMSHL GRPDGNKN-- ---------- ---------- ---------- ----------  71
PLAFA          KDTNRITATL PTINHLKKEG ASKIILISHC GRPDGLRN-- ---------- ---------- ---------- ----------  72
TETTH          KDPTRIQGSV PSIKKILEQN PKGLVLMSHL GRPDGNRV-- ---------- ---------- ---------- ----------  73
TRYBR_Gly      TNDYRIRAAI PTIRRVIDQG G-ICILLSHL GRPRGVSMVA GVRDIRRRYH EAQFHDNKGK TAFFSVLPGE EKVKILAKSS 122
TRYCO_Gly      TNDYRIRATI PTIQRIIDQG G-ICILLSHL GRPAGISMTT AVRDVRRRYH EAHFHDNKGK TAFFSVLPPE EKIKILAQSS 122
TRYCO_Cyt      TNDYRIRSAL PTIQKVLNEG G-SCILMSHL GRPKGISISE AA-------- ---------- ---------- ----------  75
TRYBR_Cyt1     TNDYRIRSAL PAVQKVLTEG G-SCVLMSHL GRPKGVSMAE G--------- ---------- ---------- ----------  74
TRYBR_Cyt2     TNDYRIRSAL PTLKKVLTEG G-SCVLMSHL GRPKGIPMAQ A--------- ---------- ---------- ----------  74
CRIFA_Cyt      TNDFRIRSAL PTIQKVLKEG G-SCILMSHL GRPKGAKMSD P--------- ---------- ---------- ----------  74
CRIFA_Gly      DDDFRIRSAI PTIRRIVDQG G-ICILISHL GRPTGVDYDT AHAEQEKRQT PVQLPNSRGK TAFFSGLRGD AKATILAWSS 119
CORGL          TDKGRIIASL PTLKALSEGG AK-VIVMAHL GRPKGEVN-- ---------- ---------- ---------- ----------  72
MYCLE          TDLGRVTASV PTLKALLEAG AK-IVVAAHL GRPKNGPD-- ---------- ---------- ---------- ----------  72
XANFL          TDATRLKAIL PTIRDITGKG GKAVLLA-HL GRPKGRDES- ---------- ---------- ---------- ----------  71
ZYMMO          TDDTRLRAAI PTVNELAEKG AKVLILA-HF GRPKGQPNP- ---------- ---------- ---------- ----------  70
ESCCO          TSDARIRASL PTIELALKQG AK-VMVTSHL GRPT------ ---------- ---------- ---------- ----------  64
HAEIN          TSDARIRATI PTLKLALEKG AK-VMVTSHL GRPT------ ---------- ---------- ---------- ----------  64
CHLRE_Cyt      TDASRITAVL PTIRLLASRG AR-LILASHF GRPE------ ---------- ---------- ---------- ----------  84
VOLCA_Cyt      TDSSRIKAVL PTIKLLLQRG SR-LILASHF GRPE------ ---------- ---------- ---------- ----------  89
METBR          LDDTKIKLHA ETIDEISKKG AKTVVLA-HQ SRP------- ---------- ---------- ---------- ----------  67
METFE          LDDTRMRLHS KTLKELVDEN AKVAILA-HQ SRP------- ---------- ---------- ---------- ----------  66
PICSI_Cyt      LDQYRIDVTL PTLYNITERG GRP-ILMTHV GRP------- ---------- ---------- ---------- ----------  90
MICSP_Cyt      HDTFRVDQSI ATLYNIVERG GRP-ILMTHV NRP------- ---------- ---------- ---------- ----------  65
HALVA          QDNRRFDRHA ETVKELADRG FEVAVLA-HQ GRP------- ---------- ---------- ---------- ----------  63
```

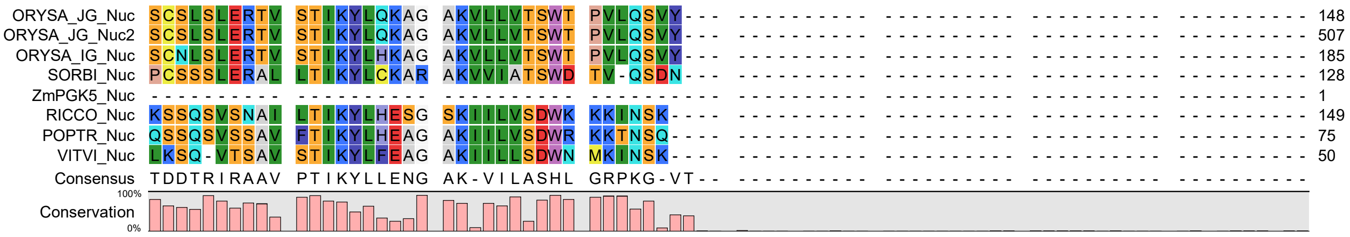
ORYSA_JG_Nuc SCSLSLERTV STIKYLQKAG AKVLLVTSWT PVLQSVY 148
ORYSA_JG_Nuc2 SCSLSLERTV STIKYLQKAG AKVLLVTSWT PVLQSVY 507
ORYSA_IG_Nuc SCNLSLERTV STIKYLHKAG AKVLLVTSWT PVLQSVY 185
SORBI_Nuc PCSSSLERAL LTIKYLCKAR AKVVIATSWD TV-QSDN 128
ZmPGK5_Nuc 1
RICCO_Nuc KSSQSVSNAI LTIKYLHESG SKIILVSDWK KKINSK 149
POPTR_Nuc QSSQSVSSAV FTIKYLHEAG AKIILVSDWR KKTNSQ 75
VITVI_Nuc LKSQ-VTSAV STIKYLFEAG AKIILLSDWN MKINSK 50
Consensus TDDTRIRAAV PTIKYLLENG AK-VILASHL GRPKG-VT
Conservation
100%
0%

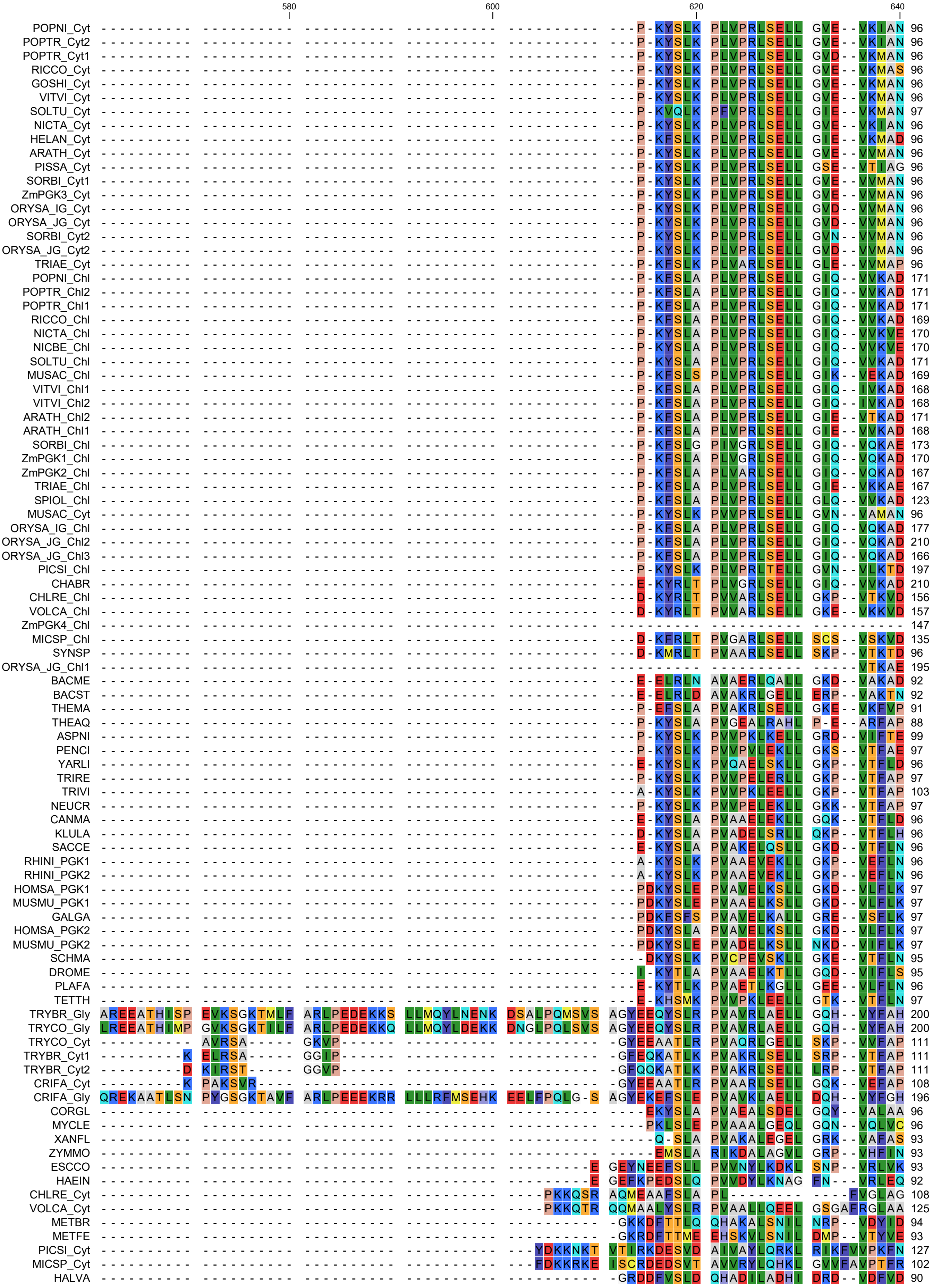

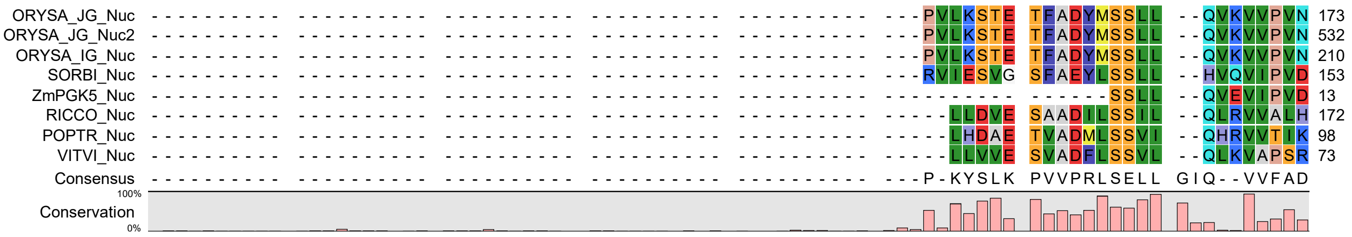
ORYSA_JG_Nuc PVLKSTE TFADYMSSLL QVKVVPVN 173
ORYSA_JG_Nuc2 PVLKSTE TFADYMSSLL QVKVVPVN 532
ORYSA_IG_Nuc PVLKSTE TFADYMSSLL QVKVVPVN 210
SORBI_Nuc RVIESVG SFAEYLSSLL HVQVIPVD 153
ZmPGK5_Nuc SSLL QVEVIPVD 13
RICCO_Nuc LLDVE SAADILSSIL QLRVVALH 172
POPTR_Nuc LHDAE TVADMLSSVI QHRVVTIK 98
VITVI_Nuc LLVVE SVADFLSSVL QLKVAPSR 73
Consensus P-KYSLK PVVPRLSELL GIQ--VVFAD
100%
Conservation
0%

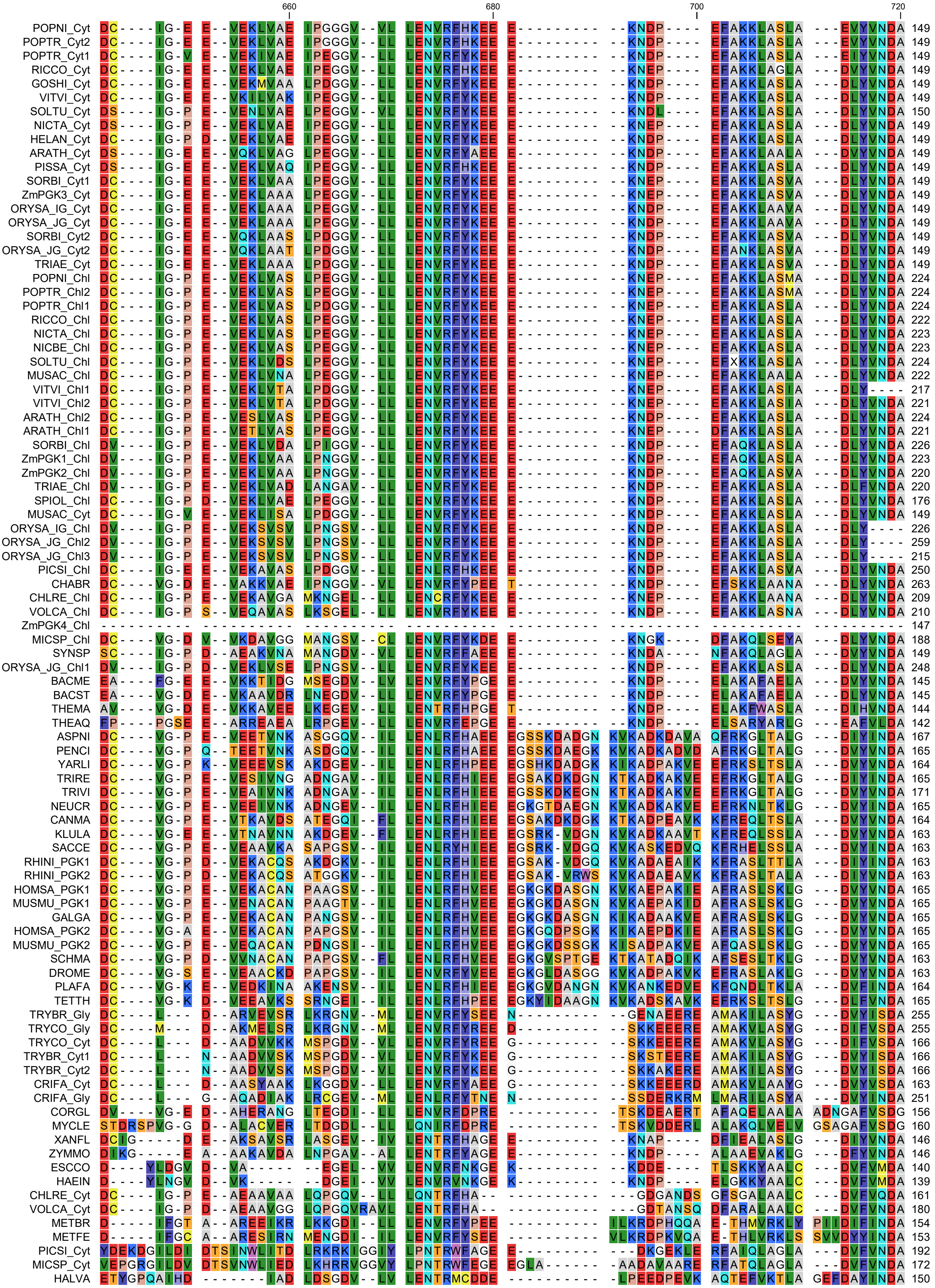
660
680
700
720
POPNI_Cyt
POPTR_Cyt2
POPTR_Cyt1
RICCO_Cyt
GOSHI_Cyt
VITVI_Cyt
SOLTU_Cyt
NICTA_Cyt
HELAN_Cyt
ARATH_Cyt
PISSA_Cyt
SORBI_Cyt1
ZmPGK3_Cyt
ORYSA_IG_Cyt
ORYSA_JG_Cyt
SORBI_Cyt2
ORYSA_JG_Cyt2
TRIAE_Cyt
POPNI_Chl
POPTR_Chl2
POPTR_Chl1
RICCO_Chl
NICTA_Chl
NICBE_Chl
SOLTU_Chl
MUSAC_Chl
VITVI_Chl1
VITVI_Chl2
ARATH_Chl2
ARATH_Chl1
SORBI_Chl
ZmPGK1_Chl
ZmPGK2_Chl
TRIAE_Chl
SPIOL_Chl
MUSAC_Cyt
ORYSA_IG_Chl
ORYSA_JG_Chl2
ORYSA_JG_Chl3
PICSI_Chl
CHABR
CHLRE_Chl
VOLCA_Chl
ZmPGK4_Chl
MICSP_Chl
SYNSP
ORYSA_JG_Chl1
BACME
BACST
THEMA
THEAQ
ASPNI
PENCI
YARLI
TRIRE
TRIVI
NEUCR
CANMA
KLULA
SACCE
RHINI_PGK1
RHINI_PGK2
HOMSA_PGK1
MUSMU_PGK1
GALGA
HOMSA_PGK2
MUSMU_PGK2
SCHMA
DROME
PLAFA
TETTH
TRYBR_Gly
TRYCO_Gly
TRYCO_Cyt
TRYBR_Cyt1
TRYBR_Cyt2
CRIFA_Cyt
CRIFA_Gly
CORGL
MYCLE
XANFL
ZYMMO
ESCCO
HAEIN
CHLRE_Cyt
VOLCA_Cyt
METBR
METFE
PICSI_Cyt
MICSP_Cyt
HALVA

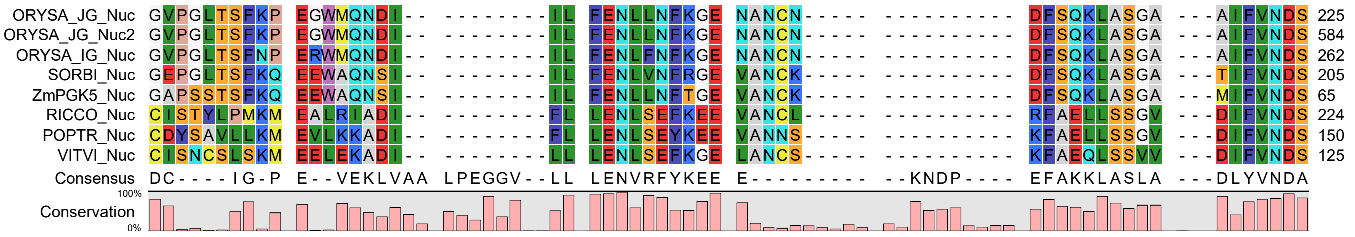

ORYSA_JG_Nuc GVPGLTSFKP EGWMQNDI-- ---------IL FENLLNFKGE NANCN------ ----------- DFSQKLASGA ---AIFVNDS 225
ORYSA_JG_Nuc2 GVPGLTSFKP EGWMQNDI-- ---------IL FENLLNFKGE NANCN------ ----------- DFSQKLASGA ---AIFVNDS 584
ORYSA_IG_Nuc GVPGLTSFNP ERWMQNDI-- ---------IL FENLFNFKGE NANCN------ ----------- DFSQKLASGA ---AIFVNDS 262
SORBI_Nuc GEPGLTSFKQ EEWAQNSI-- ---------IL FENLVNFRGE VANCK------ ----------- DFSQKLASGA ---TIFVNDS 205
ZmPGK5_Nuc GAPSSTSFKQ EEWAQNSI-- ---------IL FENLLNFTGE VANCK------ ----------- DFSQKLASGA ---MIFVNDS 65
RICCO_Nuc CISTYLPMKM EALRIADI-- ---------FL LENLSEFKEE VANCL------ ----------- RFAELLSSGV ---DIFVNDS 224
POPTR_Nuc CDYSAVLLKM EVLKKADI-- ---------FL LENLSEYKEE VANNS------ ----------- KFAELLSSGV ---DIFVNDS 150
VITVI_Nuc CISNCSLSKM EELEKADI-- ---------LL LENLSEFKGE LANCS------ ----------- KFAEQLSSVV ---DIFVNDS 125
Consensus DC----IG-P E--VEKLVAA LPEGGV--LL LENVRFYKEE E---------- --KNDP---- EFAKKLASLA ---DLYVNDA
100%
Conservation
0%

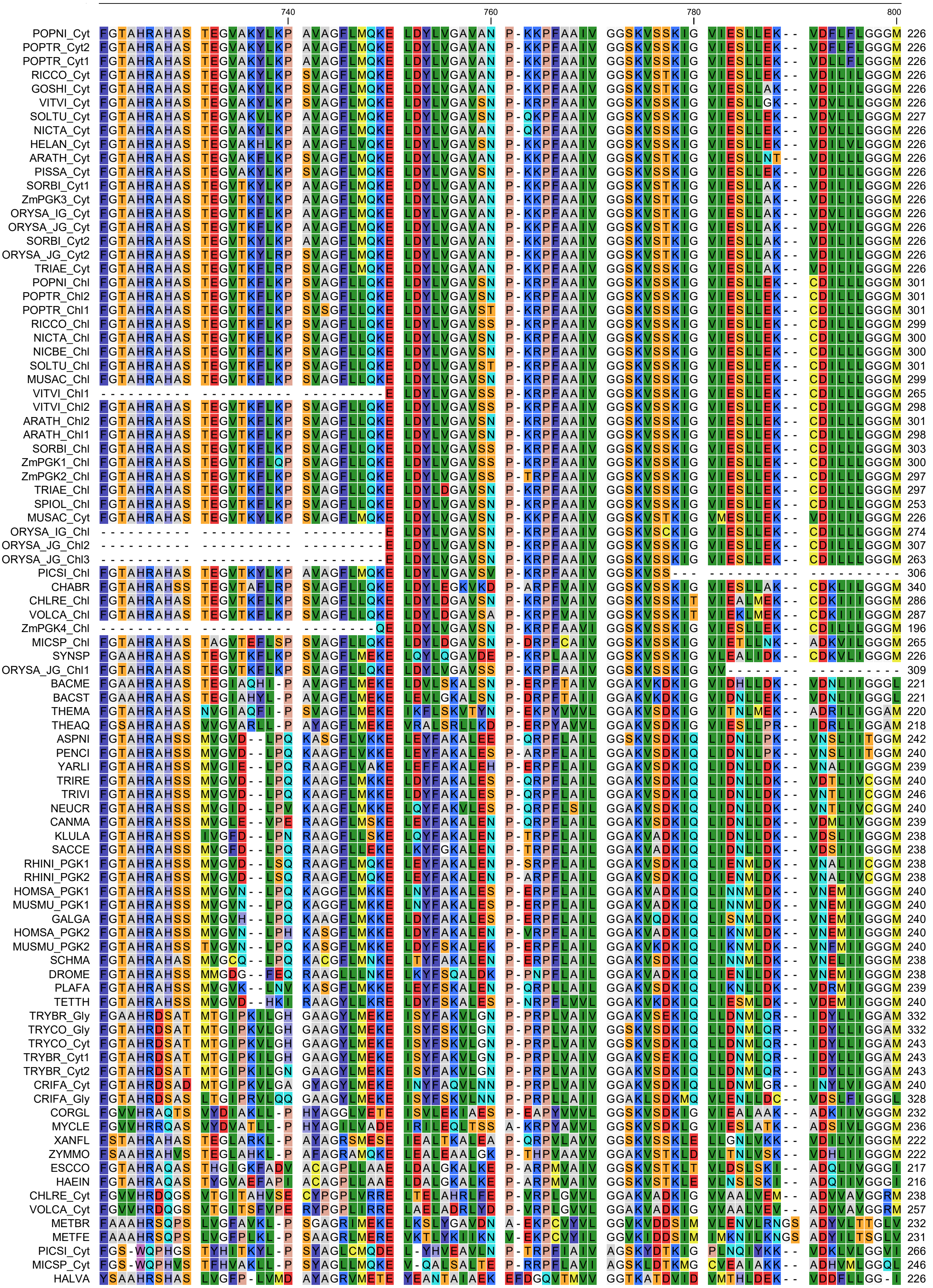
740 760 780 800
POPNI_Cyt FGTAHRAHAS TEGVAKYLKP AVAGFLMQKE LDYLVGAVAN P-KKPFAAIV GGSKVSSKIG VIESLLEK-- VDFLFLGGGM 226
POPTR_Cyt2 FGTAHRAHAS TEGVAKYLKP AVAGFLMQKE LDYLVGAVAN P-KKPFAAIV GGSKVSSKIG VIESLLEK-- VDFLFLGGGM 226
POPTR_Cyt1 FGTAHRAHAS TEGVAKYLKP AVAGFLMQKE LDYLVGAVAN P-KKPFAAIV GGSKVSSKIG VIESLLEK-- VDLLFLGGGM 226
RICCO_Cyt FGTAHRAHAS TEGVAKYLKP SVAGFLMQKE LDYLVGAVAN P-KKPFAAIV GGSKVSTKIG VIESLLEK-- VDILILGGGM 226
GOSHI_Cyt FGTAHRAHAS TEGVAKYLKP SVAGFLMQKE LDYLVGAVAN P-KKPFAAIV GGSKVSTKIG VIESLLAK-- VDILILGGGM 226
VITVI_Cyt FGTAHRAHAS TEGVAKYLKP SVAGFLMQKE LDYLVGAVSN P-KRPFAAIV GGSKVSSKIG VIESLLGK-- VDVLLLGGGM 226
SOLTU_Cyt FGTAHRAHAS TEGVAKVLKP AVAGFLMQKE LDYLVGAVSN P-QKPFAAIV GGSKVSSKIG VIESLLEK-- VDVLLLGGGM 227
NICTA_Cyt FGTAHRAHAS TEGVAKYLKP AVAGFLMQKE LDYLVGAVAN P-QKPFAAIV GGSKVSSKIG VIESLLEK-- VDVLLLGGGM 226
HELAN_Cyt FGTAHRAHAS TEGVAKHLKP AVAGFLVQKE LDYLVGAVSN P-KKPFAAIV GGSKVSSKIG VIESLLEK-- VDILVLGGGM 226
ARATH_Cyt FGTAHRAHAS TEGVAKFLKP SVAGFLMQKE LDYLVGAVAN P-KKPFAAIV GGSKVSTKIG VIESLLNT-- VDILLLGGGM 226
PISSA_Cyt FGTAHRAHAS TEGVAKYLKP SVAGFLMQKE LDYLVGAVSN P-KKPFAAIV GGSKVSSKIG VIESLLEK-- VDILILGGGM 226
SORBI_Cyt1 FGTAHRAHAS TEGVTKYLKP AVAGFLMQKE LDYLVGAVAN P-KKPFAAIV GGSKVSTKIG VIESLLAK-- VDILILGGGM 226
ZmPGK3_Cyt FGTAHRAHAS TEGVTKYLKP AVAGFLMQKE LDYLVGAVAN P-KKPFAAIV GGSKVSTKIG VIESLLAK-- VDILILGGGM 226
ORYSA_IG_Cyt FGTAHRAHAS TEGVTKFLKP AVAGFLMQKE LDYLVGAVAN P-KKPFAAIV GGSKVSTKIG VIESLLAK-- VDVLILGGGM 226
ORYSA_JG_Cyt FGTAHRAHAS TEGVTKFLKP AVAGFLMQKE LDYLVGAVAN P-KKPFAAIV GGSKVSTKIG VIESLLAK-- VDVLILGGGM 226
SORBI_Cyt2 FGTAHRAHAS TEGVTKYLKP AVAGFLMQKE LDYLVGAVAN P-KKPFAAIV GGSKVSTKIG VIESLLAK-- VDILILGGGM 226
ORYSA_JG_Cyt2 FGTAHRAHAS TEGVTKYLRP SVAGFLMQKE LDYLVGAVAN P-KKPFAAIV GGSKVSTKIG VIESLLAK-- VDILILGGGM 226
TRIAE_Cyt FGTAHRAHAS TEGVTKFLRP SVAGFLMQKE LDYLVGAVAN P-KKPFAAIV GGSKVSSKIG VIESLLAK-- VDILILGGGM 226
POPNI_Chl FGTAHRAHAS TEGVTKFLKP SVAGFLLQKE LDYLVGAVSN P-KRPFAAIV GGSKVSSKIG VIESLLEK-- CDILLLGGGM 301
POPTR_Chl2 FGTAHRAHAS TEGVTKFLKP SVAGFLLQKE LDYLVGAVSN P-KRPFAAIV GGSKVSSKIG VIESLLEK-- CDILLLGGGM 301
POPTR_Chl1 FGTAHRAHAS TEGVTKFLKP SVSGFLLQKE LDYLVGAVST P-KRPFAAIV GGSKVSSKIG VIESLLEK-- CDILLLGGGM 301
RICCO_Chl FGTAHRAHAS TEGVTKFLKP SVAGFLLQKE LDYLVGAVSS P-KRPFAAIV GGSKVSSKIG VIESLLEK-- CDILLLGGGM 299
NICTA_Chl FGTAHRAHAS TEGVTKFLKP SVAGFLLQKE LDYLVGAVSN P-KRPFAAIV GGSKVSSKIG VIESLLEK-- CDILLLGGGM 300
NICBE_Chl FGTAHRAHAS TEGVTKFLKP SVAGFLLQKE LDYLVGAVSN P-KRPFAAIV GGSKVSSKIG VIESLLEK-- CDILLLGGGM 300
SOLTU_Chl FGTAHRAHAS TEGVTKFLKP SVAGFLLQKE LDYLVGAVST P-KRPFAAIV GGSKVSSKIG VIESLLEK-- CDILLLGGGM 301
MUSAC_Chl FGTAHRAHAS TEGVTKFLKP SVAGFLLQKE LDYLVGAVSN P-KRPFAAIV GGSKVSSKIG VIESLLEK-- CDILLLGGGM 299
VITVI_Chl1 ---------- ---------- ---------E LDYLVGAVSS P-KRPFAAIV GGSKVSSKIG VIESLLEK-- CDILLLGGGM 265
VITVI_Chl2 FGTAHRAHAS TEGVTKFLKP SVAGFLLQKE LDYLVGAVSS P-KRPFAAIV GGSKVSSKIG VIESLLEK-- CDILLLGGGM 298
ARATH_Chl2 FGTAHRAHAS TEGVTKFLKP SVAGFLLQKE LDYLVGAVSN P-KRPFAAIV GGSKVSSKIG VIESLLEK-- CDILLLGGGM 301
ARATH_Chl1 FGTAHRAHAS TEGVTKFLKP SVAGFLLQKE LDYLVGAVSN P-KRPFAAIV GGSKVSSKIG VIESLLEK-- CDILLLGGGM 298
SORBI_Chl FGTAHRAHAS TEGVTKFLKP SVAGFLLQKE LDYLVGAVSS P-KRPFAAIV GGSKVSSKIG VIESLLEK-- CDILLLGGGM 303
ZmPGK1_Chl FGTAHRAHAS TEGVTKFLQP SVAGFLLQKE LDYLVGAVSS P-KRPFAAIV GGSKVSSKIG VIESLLEK-- CDILLLGGGM 300
ZmPGK2_Chl FGTAHRAHAS TEGVTKFLKP SVAGFLLQKE LDYLVGAVSS P-TRPFAAIV GGSKVSSKIG VIESLLEK-- CDILLLGGGM 297
TRIAE_Chl FGTAHRAHAS TEGVTKFLKP SVAGFLLQKE LDYLDGAVSN P-KRPFAAIV GGSKVSSKIG VIESLLEK-- CDILLLGGGM 297
SPIOL_Chl FGTAHRAHAS TEGVTKFLKP SVAGFLLQKE LDYLVGAVSN P-KRPFAAIV GGSKVSSKIG VIESLLEK-- CDILLLGGGM 253
MUSAC_Cyt FGTAHRAHAS TEGVTKYLKP SVAGFLMQKE LDYLVGAVSN P-KKPFAAIV GGSKVSTKIG VMESLLEK-- VDILLLGGGM 226
ORYSA_IG_Chl ---------- ---------- ---------E LDYLVGAVSN P-KRPFAAIV GGSKVSCKIG VIESLLEK-- CDILLLGGGM 274
ORYSA_JG_Chl2 ---------- ---------- ---------E LDYLVGAVSN P-KRPFAAIV GGSKVSSKIG VIESLLEK-- CDILLLGGGM 307
ORYSA_JG_Chl3 ---------- ---------- ---------E LDYLVGAVSN P-KRPFAAIV GGSKVSSKIG VIESLLEK-- CDILLLGGGM 263
PICSI_Chl FGTAHRAHAS TEGVTKYLKP AVAGFLMQKE LDYLVGAVSV P-KRPFAAIV GGSKVSS--- ---------- ---------- 306
CHABR FGTAHRAHSS TEGVTAFLRP SVAGFLLQKE LDYLEGKVKD P-ARPFVAIV GGSKVSSKIG VIESLLAK-- CDKLILGGGM 340
CHLRE_Chl FGTAHRAHSS TEGVTKFLKP SVAGFLLQKE LDYLDGAVSN P-KRPFVAIV GGSKVSSKIT VIEALMEK-- CDKIIIGGGM 286
VOLCA_Chl FGTAHRAHSS TEGVTKFLKP SVAGFLLQKE LDYLDGAVSA P-KRPFVAIV GGSKVSSKIT VIEKLMEK-- CDKIIIGGGM 287
ZmPGK4_Chl ---------- ---------- --------QE LDYLVGAVSN P-KRPFAAVI GGSKVSSKIG VIESLLEK-- CDILLLGGGM 196
MICSP_Chl FGTAHRAHAS TAGVTEFLSP SVAGFLLQKE LDYLDGAVSN P-DRPFCAIV GGSKVSSKIG VIETLLNK-- ADKVILGGGM 265
SYNSP FGAAHRAHAS TEGVTKFLKP SVAGFLMEKE LQYLQGAVDE P-KRPLAAIV GGSKVSSKIG VLEALIDK-- CDKVLIGGGM 226
ORYSA_JG_Chl1 FGTAHRAHAS TEGVTKFLKP SVAGFLLQKE LDYLVGAVSS P-KRPFAAIV GGSKVSSKIG VV-------- ---------- 309
BACME FGAAHRAHAS TEGIAQHI-P AVAGFLMEKE LDVLSKALSN P-ERPFTAIV GGAKVKDKIG VIDHLLDK-- VDNLIIGGGL 221
BACST FGAAHRAHAS TEGIAHYL-P AVAGFLMEKE LEVLGKALSN P-DRPFTAII GGAKVKDKIG VIDNLLEK-- VDNLIIGGGL 221
THEMA FGTAHRAHAS NVGIAQFI-P SVAGFLMEKE IKFLSKVTYN P-EKPYVVVL GGAKVSDKIG VITNLMEK-- ADRILIGGAM 220
THEAQ FGSAHRAHAS VVGVARLL-P AYAGFLMEKE VRALSRLLKD P-ERPYAVVL GGAKVSDKIG VIESLLPR-- IDRLLIGGAM 218
ASPNI FGTAHRAHSS MVGVD--LPQ KASGFLVKKE LEYFAKALEE P-QRPFLAIL GGSKVSDKIQ LIDNLLPK-- VNSLIITGGM 242
PENCI FGTAHRAHSS MVGVD--LPQ KAAGFLVKKE LEYFAKALES P-ARPFLAIL GGSKVSDKIQ LIDNLLPK-- VNSLIITGGM 240
YARLI FGTAHRAHSS MVGIE--LPQ RAAGFLVAKE LEFFAKALEH P-ERPFLAIL GGAKVSDKIQ LIDNLLDK-- VNALIIGGGM 239
TRIRE FGTAHRAHSS MVGVD--LPQ KAAGFLMKKE LDYFAKALES P-QRPFLAIL GGAKVSDKIQ LIDNLLDK-- VDTLIVCGGM 240
TRIVI FGTAHRAHSS MVGVD--LPQ KAAGFLMKKE LDYFAKALES P-QRPFLAIL GGAKVSDKIQ LIDNLLDK-- VNTLIICGGM 246
NEUCR FGTAHRAHSS MVGID--LPV KAAGFLMKKE LQYFAKVLES P-QRPFLSIL GGAKVSDKIQ LIDNLLDK-- VNTLIVCGGM 240
CANMA FGTAHRAHSS MVGLE--VPE RAAGFLMSKE LEYFAKALEN P-QRPFLAIL GGAKVSDKIQ LIDNLLDK-- VDMLIVGGGM 239
KLULA FGTAHRAHSS IVGFD--LPN RAAGFLLSKE LQYFAKALEN P-TRPFLAIL GGAKVADKIQ LIDNLLDK-- VDSLIIGGGM 238
SACCE FGTAHRAHSS MVGFD--LPQ RAAGFLLEKE LKYFGKALEN P-TRPFLAIL GGAKVADKIQ LIDNLLDK-- VDSIIIGGGM 238
RHINI_PGK1 FGTAHRAHSS MVGVD--LSQ RAAGFLMQKE LEYFAKALEN P-SRPFLAIL GGAKVSDKIQ LIENMLDK-- VNALIVCGGM 238
RHINI_PGK2 FGTAHRAHSS MVGVD--LSQ RAAGFLMQKE LEYFAKALEN P-ARPFLAIL GGAKVSDKIQ LIENMLDK-- VNALIVCGGM 238
HOMSA_PGK1 FGTAHRAHSS MVGVN--LPQ KAGGFLMKKE LNYFAKALES P-ERPFLAIL GGAKVADKIQ LINNMLDK-- VNEMIIGGGM 240
MUSMU_PGK1 FGTAHRAHSS MVGVN--LPQ KAGGFLMKKE LNYFAKALES P-ERPFLAIL GGAKVADKIQ LINNMLDK-- VNEMIIGGGM 240
GALGA FGTAHRAHSS MVGVH--LPQ KAAGFLMKKE LDYFAKALES P-ERPFLAIL GGAKVQDKIQ LISNMLDK-- VNEMIIGGGM 240
HOMSA_PGK2 FGTAHRAHSS MVGVN--LPH KASGFLMKKE LDYFAKALEN P-VRPFLAIL GGAKVADKIQ LIKNMLDK-- VNEMIIGGGM 240
MUSMU_PGK2 FGTAHRAHSS TVGVN--LPQ KASGFLMKKE LDYFSKALEK P-ERPFLAIL GGAKVKDKIQ LIKNMLDK-- VNFMIIGGGM 240
SCHMA FGTAHRAHSS MVGCQ--LPQ KACGFLMNKE LTYFAKALEN P-ERPFLAIL GGAKVSDKIQ LINNMLDK-- VNELIIGGGM 238
DROME FGTAHRAHSS MMGDG--FEQ RAAGLLLNKE LKYFSQALDK P-PNPFLAIL GGAKVADKIQ LIENLLDK-- VNEMIIGGGM 238
PLAFA FGTAHRAHSS MVGVK--LNV KASGFLMKKE LEYFSKALEN P-QRPLLAIL GGAKVSDKIQ LIKNLLDK-- VDRMIIGGGM 239
TETTH FGTAHRAHSS MVGVD--HKI RAAGYLLKRE LDYFSKALES P-NRPFLVVL GGAKVKDKIQ LIESMLDK-- VDEMIIGGGM 240
TRYBR_Gly FGAAHRDSAT MTGIPKILGH GAAGYLMEKE ISYFAKVLGN P-PRPLVAIV GGAKVSEKIQ LLDNMLQR-- IDYLLIGGAM 332
TRYCO_Gly FGTAHRDSAT MTGIPKVLGH GAAGYLMEKE ISYFSKVLGN P-PRPLVAIV GGSKVSDKIQ LLDNMLQR-- IDYLLIGGAM 332
TRYCO_Cyt FGTAHRDSAT MTGIPKVLGH GAAGYLMEKE ISYFSKVLGN P-PRPLVAIV GGSKVSDKIQ LLDNMLQR-- IDYLLIGGAM 243
TRYBR_Cyt1 FGTAHRDSAT MTGIPKILGH GAAGYLMEKE ISYFAKVLGN P-PRPLVAIV GGAKVSEKIQ LLDNMLQR-- IDYLLIGGAM 243
TRYBR_Cyt2 FGTAHRDSAT MTGIPKILGN GAAGYLMEKE ISYFAKVLGN P-PRPLVAIV GGAKVSDKIQ LLDNMLQR-- IDYLLIGGAM 243
CRIFA_Cyt FGTAHRDSAD MTGIPKVLGA GYAGYLMEKE INYFAQVLNN P-PRPLVAIV GGAKVSDKIQ LLDNMLGR-- INYLVIGGAM 240
CRIFA_Gly FGTAHRDSAS LTGIPRVLQQ GAAGYLMEKE ISYFSKVLNN P-PRPLLAII GGAKLSDKMQ VLENLLDC-- VDSLFIGGGL 328
CORGL FGVVHRAQTS VYDIAKLL-P HYAGGLVETE ISVLEKIAES P-EAPYVVVL GGSKVSDKIG VIEALAAK-- ADKIIVGGGM 232
MYCLE FGVVHRRQAS VYDVATLL-P HYAGILVADE IRILEQLTSS A-KRPYAVVL GGSKVSDKLG VIESLATK-- ADSIVLGGGM 236
XANFL FSTAHRAHAS TEGLARKL-P AYAGRSMESE IEALTKALEA P-QRPVLAVV GGSKVSSKLE LLGNLVKK-- VDILVIGGGM 222
ZYMMO FSAAHRAHVS TEGLAHKL-P AFAGRAMQKE LEALEAALGK P-THPVAAVV GGAKVSTKLD VLTNLVSK-- VDHLIIGGGM 222
ESCCO FGTAHRAQAS THGIGKFADV ACAGPLLAAE LDALGKALKE P-ARPMVAIV GGSKVSTKLT VLDSLSKI-- ADQLIVGGGI 217
HAEIN FGTAHRAQAS TYGVAEFAPI ACAGPLLAAE LDALGKALKE P-ARPMVAIV GGSKVSTKLE VLNSLSKI-- ADQIIVGGGI 216
CHLRE_Cyt FGVVHRDQGS VTGITAHVSE CYPGPLVRRE LTELAHRLFE P-VRPLGVVL GGAKVADKIG VVAALVEM-- ADVVAVGGRM 238
VOLCA_Cyt FGVVHRDQGS VTGITSFVPE RYPGPLIRRE LAELADRLYD P-VRPLGVVL GGAKVADKIG VVAALVEV-- ADVVAVGGRM 257
METBR FAAAHRSQPS LVGFAVKL-P SGAGRIMEKE LKSLYGAVDN A-EKPCVYVL GGVKVDDSIM VLENVLRNGS ADYVLTTGLV 232
METFE FAAAHRSQPS LVGFPLKL-P SAAGRLMERE VKTLYKIIKN V-EKPCVYIL GGVKIDDSIM IMKNILKNGS ADYILTSGLV 231
PICSI_Cyt FGS-WQPHGS TYHITKYL-P SYAGLCMQDE L-YHVEAVLN P-TRPFIAIV AGSKYDTKIG PLNQIYKK-- VDKLVLGGVI 266
MICSP_Cyt FGS-WQPHVS TFHVAKLL-P SYAGLLMQKE V-QALSALTE P-VRPLGAVI AGSKLDTKMG CVEAIAKK-- ADHVMLGGQL 246
HALVA YSAAHRSHAS LVGFP-LVMD AYAGRVMETE YEANTAIAEK EFDGQVTMVV GGTKATDVID VMTHLDEK-- VDDFLLGG-I 226

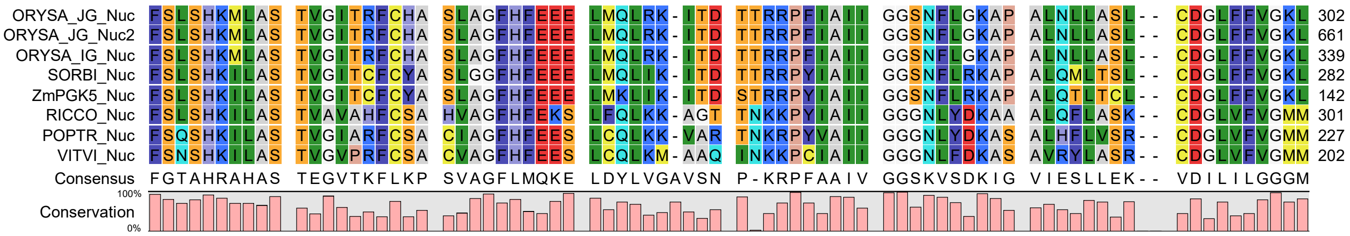
ORYSA_JG_Nuc FSLSHKMLAS TVGITRFCHA SLAGFHFEEE LMQLRK-ITD TTRRPFIAII GGSNFLGKAP ALNLLASL-- CDGLFFVGKL 302
ORYSA_JG_Nuc2 FSLSHKMLAS TVGITRFCHA SLAGFHFEEE LMQLRK-ITD TTRRPFIAII GGSNFLGKAP ALNLLASL-- CDGLFFVGKL 661
ORYSA_IG_Nuc FSLSHKMLAS TVGITRFCHA SLAGFHFEEE LMQLRK-ITD TTRRPFIAII GGSNFLGKAP ALNLLASL-- CDGLFFVGKL 339
SORBI_Nuc FSLSHKILAS TVGITCFCYA SLGGFHFEEE LMQLIK-ITD TTRRPYIAII GGSNFLRKAP ALQMLTSL-- CDGLFFVGKL 282
ZmPGK5_Nuc FSLSHKILAS TVGITCFCYA SLAGFHFEEE LMKLIK-ITD STRRPYIAII GGSNFLRKAP ALQTLTCL-- CDGLFFVGKL 142
RICCO_Nuc FSLSHKILAS TVAVAHFCSA HVAGFHFEKS LFQLKK-AGT TNKKPYIAII GGGNLYDKAA ALQFLASK-- CDGLVFVGMM 301
POPTR_Nuc FSQSHKILAS TVGIARFCSA CIAGFHFEES LCQLKK-VAR TNKRPYVAII GGGNLYDKAS ALHFLVSR-- CDGLVFVGMM 227
VITVI_Nuc FSNSHKILAS TVGVPRFCSA CVAGFHFEES LCQLKM-AAQ INKKPCIAII GGGNLFDKAS AVRYLASR-- CDGLVFVGMM 202
Consensus FGTAHRAHAS TEGVTKFLKP SVAGFLMQKE LDYLVGAVSN P-KRPFAAIV GGSKVSDKIG VIESLLEK-- VDILILGGGM
100%
Conservation
0%

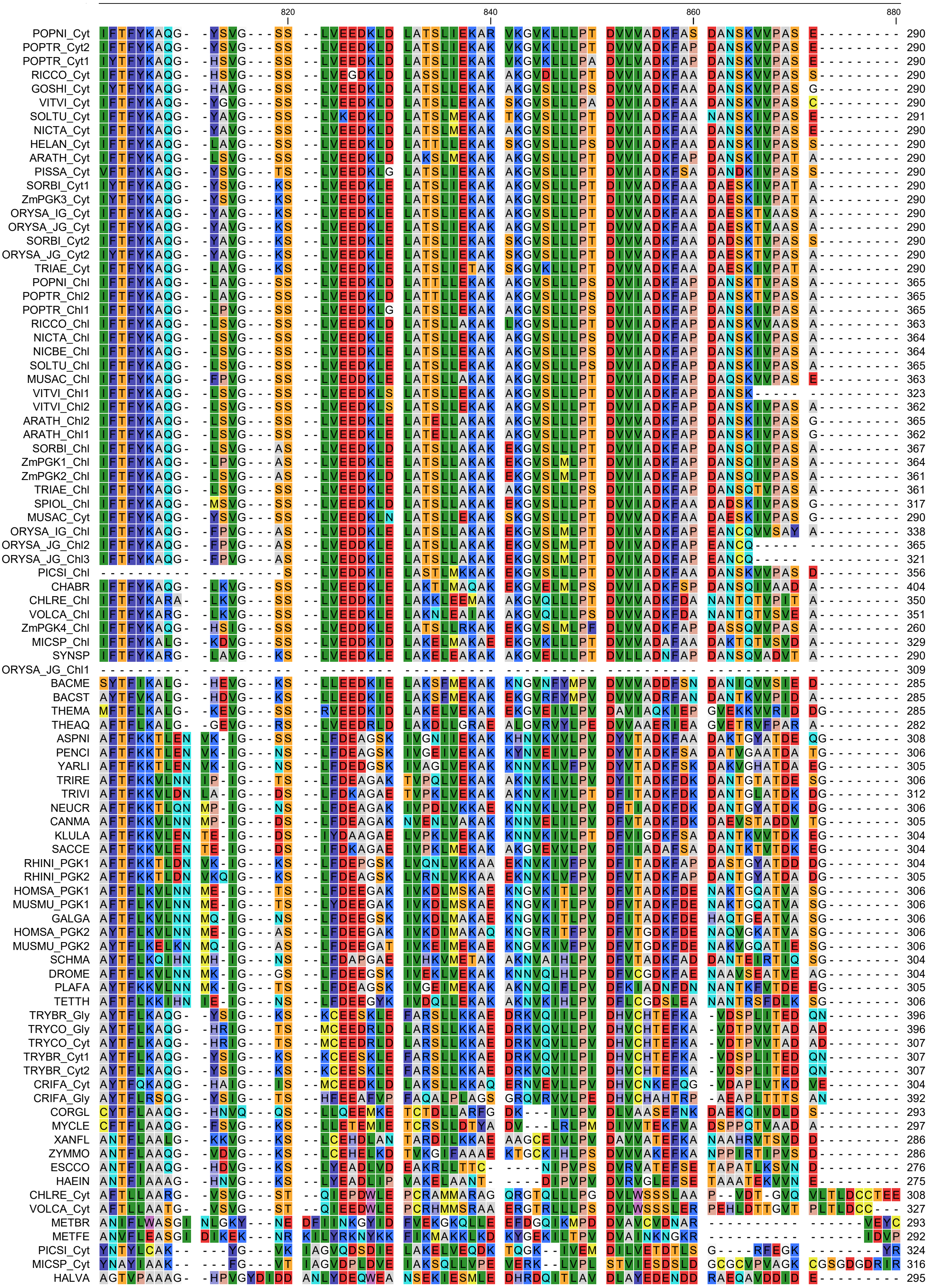
820
840
860
880
POPNI_Cyt
POPTR_Cyt2
POPTR_Cyt1
RICCO_Cyt
GOSHI_Cyt
VITVI_Cyt
SOLTU_Cyt
NICTA_Cyt
HELAN_Cyt
ARATH_Cyt
PISSA_Cyt
SORBI_Cyt1
ZmPGK3_Cyt
ORYSA_IG_Cyt
ORYSA_JG_Cyt
SORBI_Cyt2
ORYSA_JG_Cyt2
TRIAE_Cyt
POPNI_Chl
POPTR_Chl2
POPTR_Chl1
RICCO_Chl
NICTA_Chl
NICBE_Chl
SOLTU_Chl
MUSAC_Chl
VITVI_Chl1
VITVI_Chl2
ARATH_Chl2
ARATH_Chl1
SORBI_Chl
ZmPGK1_Chl
ZmPGK2_Chl
TRIAE_Chl
SPIOL_Chl
MUSAC_Cyt
ORYSA_IG_Chl
ORYSA_JG_Chl2
ORYSA_JG_Chl3
PICSI_Chl
CHABR
CHLRE_Chl
VOLCA_Chl
ZmPGK4_Chl
MICSP_Chl
SYNSP
ORYSA_JG_Chl1
BACME
BACST
THEMA
THEAQ
ASPNI
PENCI
YARLI
TRIRE
TRIVI
NEUCR
CANMA
KLULA
SACCE
RHINI_PGK1
RHINI_PGK2
HOMSA_PGK1
MUSMU_PGK1
GALGA
HOMSA_PGK2
MUSMU_PGK2
SCHMA
DROME
PLAFA
TETTH
TRYBR_Gly
TRYCO_Gly
TRYCO_Cyt
TRYBR_Cyt1
TRYBR_Cyt2
CRIFA_Cyt
CRIFA_Gly
CORGL
MYCLE
XANFL
ZYMMO
ESCCO
HAEIN
CHLRE_Cyt
VOLCA_Cyt
METBR
METFE
PICSI_Cyt
MICSP_Cyt
HALVA

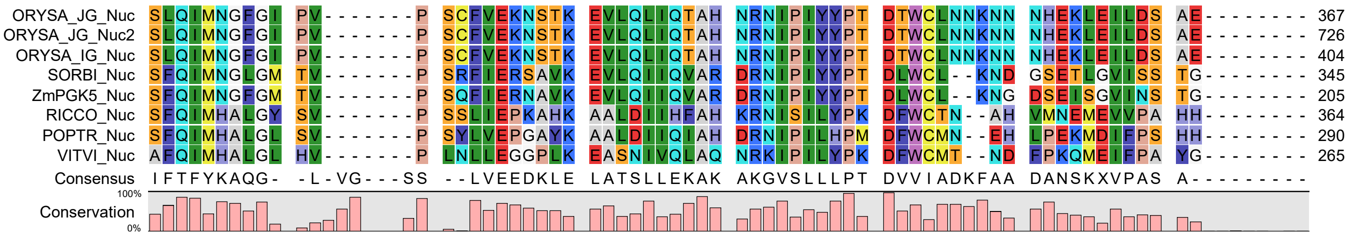
ORYSA_JG_Nuc SLQIMNGFGI PV-------P SCFVEKNSTK EVLQLIQTAH NRNIPIYYPT DTWCLNNKNN NHEKLEILDS AE-------- 367
ORYSA_JG_Nuc2 SLQIMNGFGI PV-------P SCFVEKNSTK EVLQLIQTAH NRNIPIYYPT DTWCLNNKNN NHEKLEILDS AE-------- 726
ORYSA_IG_Nuc SLQIMNGFGI PV-------P SCFVEKNSTK EVLQLIQTAH NRNIPIYYPT DTWCLNNKNN NHEKLEILDS AE-------- 404
SORBI_Nuc SFQIMNGLGM TV-------P SRFIERSAVK EVLQIIQVAR DRNIPIYYPT DLWCL--KND GSETLGVISS TG-------- 345
ZmPGK5_Nuc SFQIMNGFGM TV-------P SQFIERNAVK EVLQIIQVAR DRNIPIYYPT DLWCL--KNG DSEISGVINS TG-------- 205
RICCO_Nuc SFQIMHALGY SV-------P SSLIEPKAHK AALDIIHFAH KRNISILYPK DFWCTN--AH VMNEMEVVPA HH-------- 364
POPTR_Nuc SFQIMHALGL SV-------P SYLVEPGAYK AALDIIQIAH DRNIPILHPM DFWCMN--EH LPEKMDIFPS HH-------- 290
VITVI_Nuc AFQIMHALGL HV-------P LNLLEGGPLK EASNIVQLAQ NRKIPILYPK DFWCMT--ND FPKQMEIFPA YG-------- 265
Consensus IFTFYKAQG- -L-VG---SS --LVEEDKLE LATSLLEKAK AKGVSLLLPT DVVIADKFAA DANSKXVPAS A---------
100%
Conservation
0%

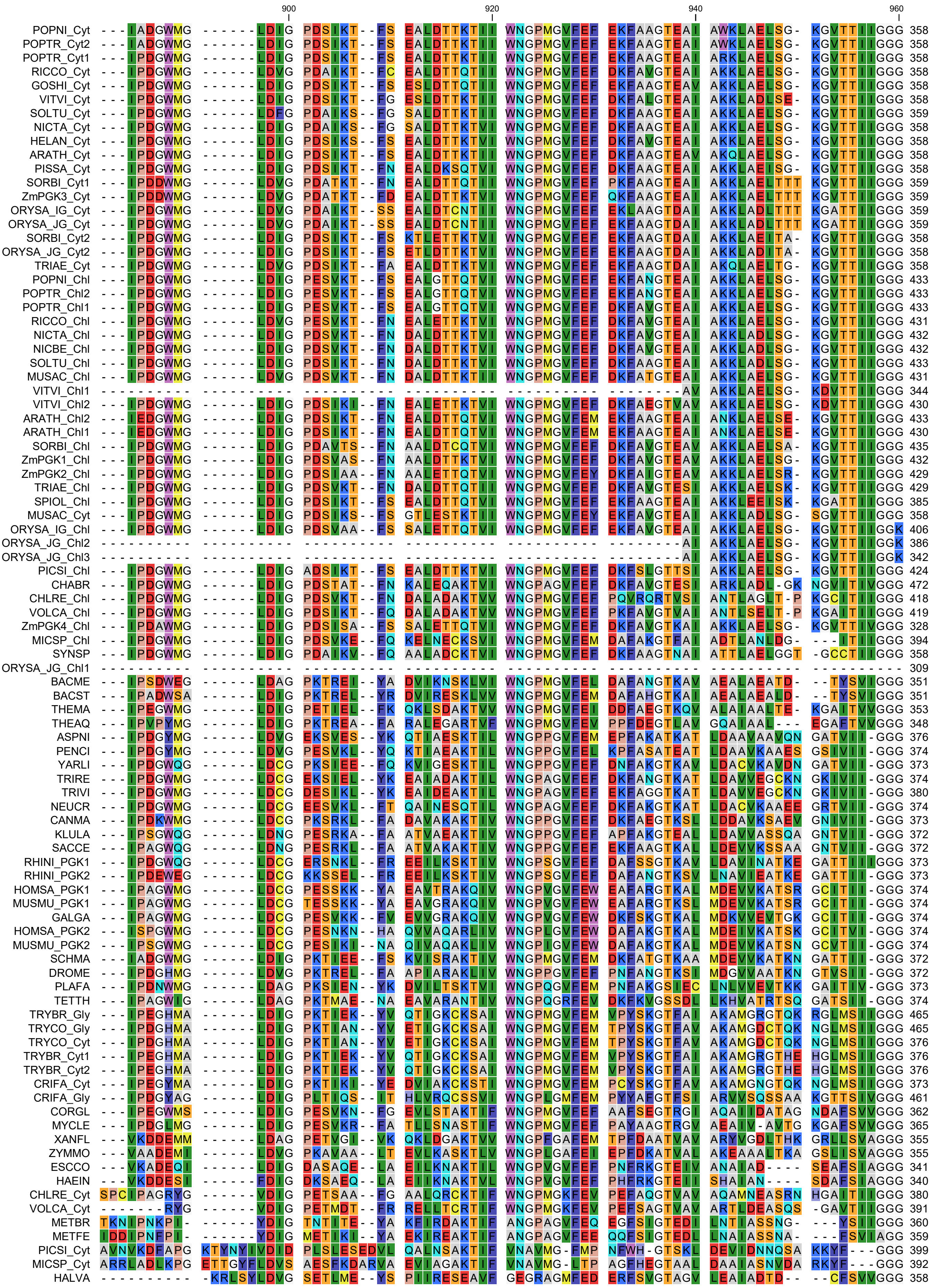
900
920
940
960
POPNI_Cyt
POPTR_Cyt2
POPTR_Cyt1
RICCO_Cyt
GOSHI_Cyt
VITVI_Cyt
SOLTU_Cyt
NICTA_Cyt
HELAN_Cyt
ARATH_Cyt
PISSA_Cyt
SORBI_Cyt1
ZmPGK3_Cyt
ORYSA_IG_Cyt
ORYSA_JG_Cyt
SORBI_Cyt2
ORYSA_JG_Cyt2
TRIAE_Cyt
POPNI_Chl
POPTR_Chl2
POPTR_Chl1
RICCO_Chl
NICTA_Chl
NICBE_Chl
SOLTU_Chl
MUSAC_Chl
VITVI_Chl1
VITVI_Chl2
ARATH_Chl2
ARATH_Chl1
SORBI_Chl
ZmPGK1_Chl
ZmPGK2_Chl
TRIAE_Chl
SPIOL_Chl
MUSAC_Cyt
ORYSA_IG_Chl
ORYSA_JG_Chl2
ORYSA_JG_Chl3
PICSI_Chl
CHABR
CHLRE_Chl
VOLCA_Chl
ZmPGK4_Chl
MICSP_Chl
SYNSP
ORYSA_JG_Chl1
BACME
BACST
THEMA
THEAQ
ASPNI
PENCI
YARLI
TRIRE
TRIVI
NEUCR
CANMA
KLULA
SACCE
RHINI_PGK1
RHINI_PGK2
HOMSA_PGK1
MUSMU_PGK1
GALGA
HOMSA_PGK2
MUSMU_PGK2
SCHMA
DROME
PLAFA
TETTH
TRYBR_Gly
TRYCO_Gly
TRYCO_Cyt
TRYBR_Cyt1
TRYBR_Cyt2
CRIFA_Cyt
CRIFA_Gly
CORGL
MYCLE
XANFL
ZYMMO
ESCCO
HAEIN
CHLRE_Cyt
VOLCA_Cyt
METBR
METFE
PICSI_Cyt
MICSP_Cyt
HALVA

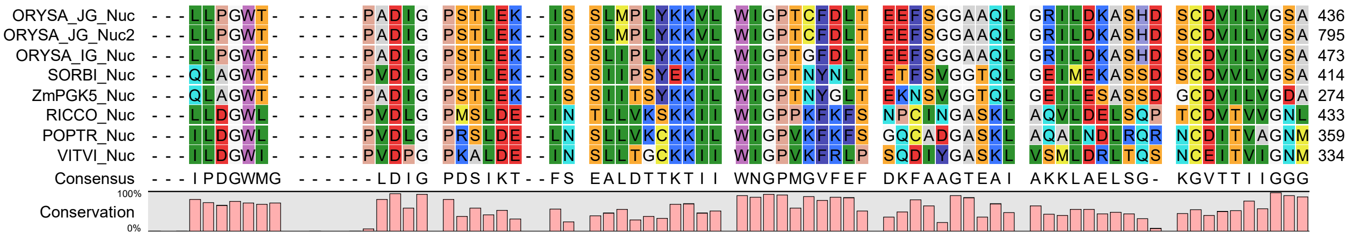

ORYSA_JG_Nuc ---LLPGWT- -----PADIG PSTLEK--IS SLMPLYKKVL WIGPTCFDLT EEFSGGAAQL GRILDKASHD SCDVILVGSA 436
ORYSA_JG_Nuc2 ---LLPGWT- -----PADIG PSTLEK--IS SLMPLYKKVL WIGPTCFDLT EEFSGGAAQL GRILDKASHD SCDVILVGSA 795
ORYSA_IG_Nuc ---LLPGWT- -----PADIG PSTLEK--IS SLIPLYKKVL WIGPTGFDLT EEFSGGAAQL GRILDKASHD SCDVILVGSA 473
SORBI_Nuc ---QLAGWT- -----PVDIG PSTLEK--IS SIIPSYEKIL WIGPTNYNLT ETFSVGGTQL GEIMEKASSD SCDVVLVGSA 414
ZmPGK5_Nuc ---QLAGWT- -----PADIG PSTLEK--IS SIITSYKKIL WIGPTNYGLT EKNSVGGTQL GEILESASSD GCDVILVGDA 274
RICCO_Nuc ---LLDGWL- -----PVDLG PMSLDE--IN TLLVKSKKII WIGPPKFKFS NPCINGASKL AQVLDELSQP TCDVTVVGNL 433
POPTR_Nuc ---ILDGWL- -----PVDLG PRSLDE--LN SLLVKCKKIL WIGPVKFKFS GQCADGASKL AQALNDLRQR NCDITVAGNM 359
VITVI_Nuc ---ILDGWI- -----PVDPG PKALDE--IN SLLTGCKKII WIGPVKFRLP SQDIYGASKL VSMLDRLTQS NCEITVIGNM 334
Consensus ---IPDGWMG ------LDIG PDSIKT--FS EALDTTKTII WNGPMGVFEF DKFAAGTEAI AKKLAELSG- KGVTTIIGGG
100%
Conservation
0%

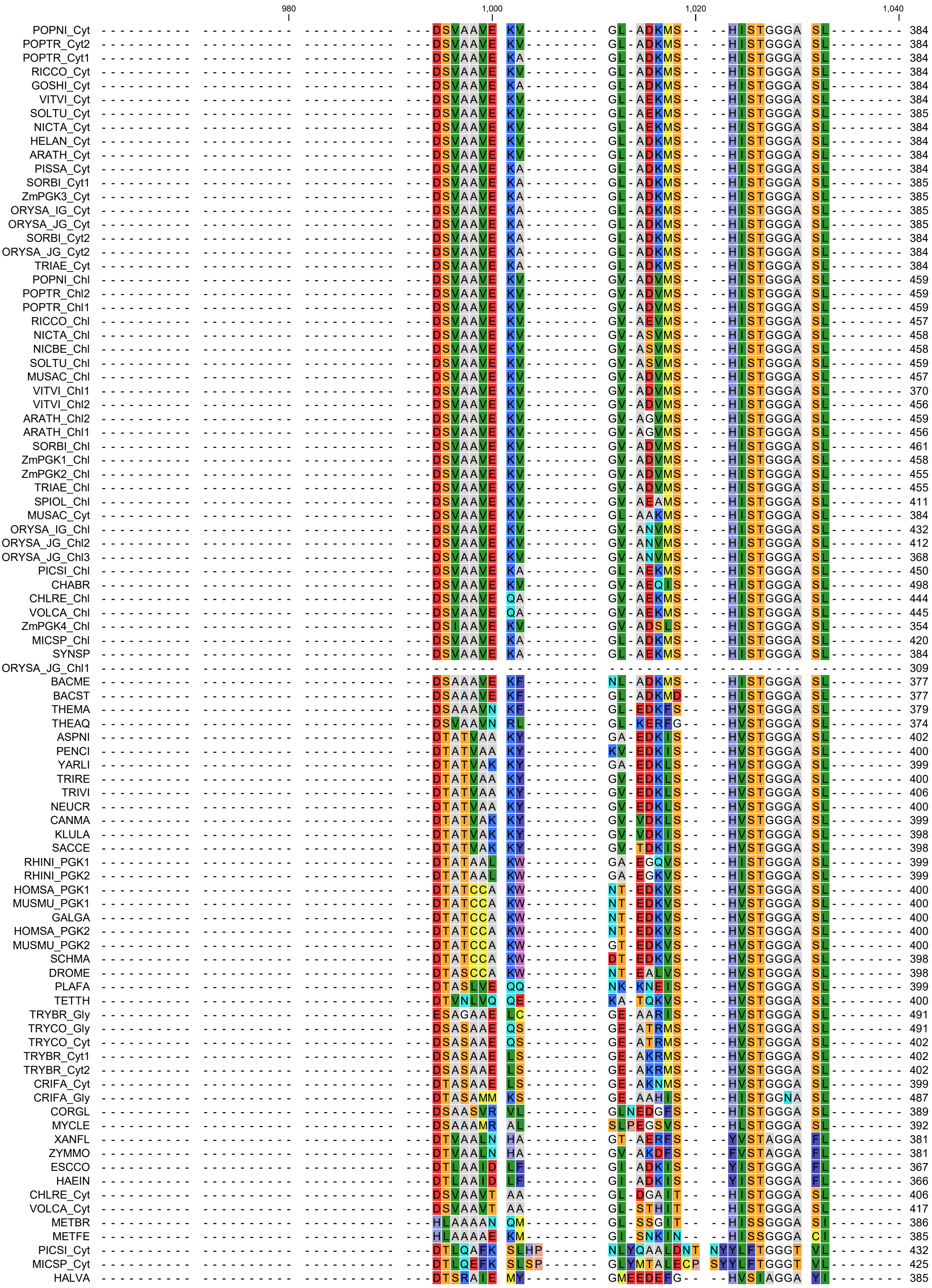
980
1,000
1,020
1,040

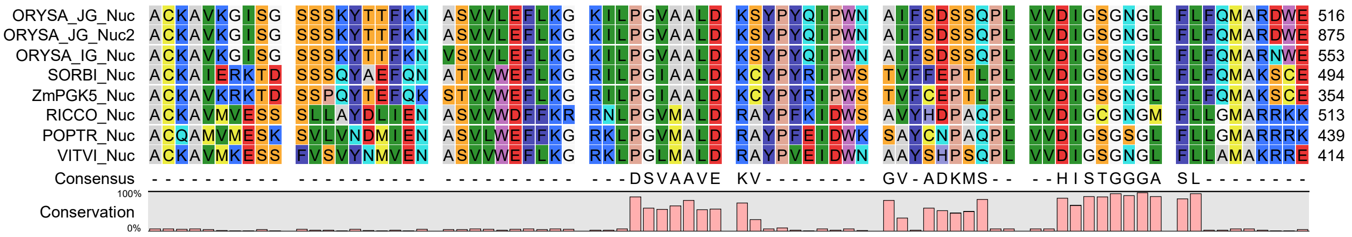
ORYSA_JG_Nuc ACKAVKGISG SSSKYTTFKN ASVVLEFLKG KILPGVAALD KSYPYQIPWN AIFSDSSQPL VVDIGSGNGL FLFQMARDWE 516
ORYSA_JG_Nuc2 ACKAVKGISG SSSKYTTFKN ASVVLEFLKG KILPGVAALD KSYPYQIPWN AIFSDSSQPL VVDIGSGNGL FLFQMARDWE 875
ORYSA_IG_Nuc ACKAVKGISG SSSKYTTFKN VSVVLEFLKG KILPGVAALD KSYPYQIPWN AIFSDSSQPL VVDIGSGNGL FLFQMARNWE 553
SORBI_Nuc ACKAIERKTD SSSQYAEFQN ATVVWEFLKG RILPGIAALD KCYPYRIPWS TVFFEPTLPL VVDIGSGNGL FLFQMAKSCE 494
ZmPGK5_Nuc ACKAVKRKTD SSPQYTEFQK STVVWEFLKG RILPGIAALD KCYPYRIPWS TVFCEPTLPL VVDIGSGNGL FLFQMAKSCE 354
RICCO_Nuc ACKAVMVESS SLLAYDLIEN ASVVWDFFKR RNLPGVMALD RAYPFKIDWS AVYHDPAQPL VVDIGCGNGM FLLGMARRKK 513
POPTR_Nuc ACQAMVMESK SVLVNDMIEN ASVLWEFFKG RKLPGVMALD RAYPFEIDWK SAYCNPAQPL VVDIGSGSGL FLLGMARRRK 439
VITVI_Nuc ACKAVMKESS FVSVYNMVEN ASVVWEFLKG RKLPGLMALD RAYPVEIDWN AAYSHPSQPL VVDIGSGNGL FLLAMAKRRE 414
Consensus ---------- ---------- ---------- ---DSVAAVE KV-------- GV-ADKMS-- --HISTGGGA SL--------
100%
Conservation
0%

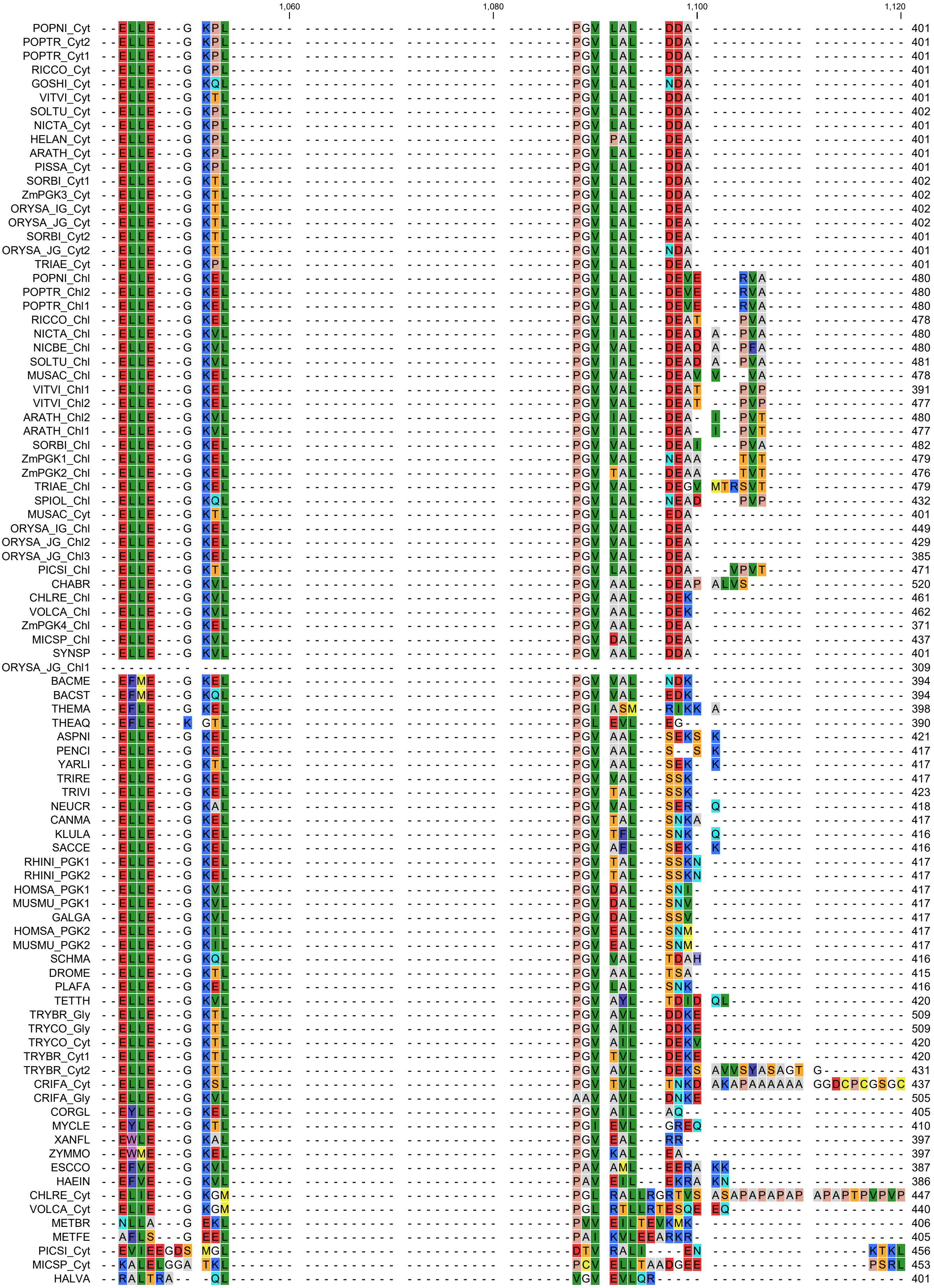
1,060
1,080
1,100
1,120
POPNI_Cyt 401
POPTR_Cyt2 401
POPTR_Cyt1 401
RICCO_Cyt 401
GOSHI_Cyt 401
VITVI_Cyt 401
SOLTU_Cyt 402
NICTA_Cyt 401
HELAN_Cyt 401
ARATH_Cyt 401
PISSA_Cyt 401
SORBI_Cyt1 402
ZmPGK3_Cyt 402
ORYSA_IG_Cyt 402
ORYSA_JG_Cyt 402
SORBI_Cyt2 401
ORYSA_JG_Cyt2 401
TRIAE_Cyt 401
POPNI_Chl 480
POPTR_Chl2 480
POPTR_Chl1 480
RICCO_Chl 478
NICTA_Chl 480
NICBE_Chl 480
SOLTU_Chl 481
MUSAC_Chl 478
VITVI_Chl1 391
VITVI_Chl2 477
ARATH_Chl2 480
ARATH_Chl1 477
SORBI_Chl 482
ZmPGK1_Chl 479
ZmPGK2_Chl 476
TRIAE_Chl 479
SPIOL_Chl 432
MUSAC_Cyt 401
ORYSA_IG_Chl 449
ORYSA_JG_Chl2 429
ORYSA_JG_Chl3 385
PICSI_Chl 471
CHABR 520
CHLRE_Chl 461
VOLCA_Chl 462
ZmPGK4_Chl 371
MICSP_Chl 437
SYNSP 401
ORYSA_JG_Chl1 309
BACME 394
BACST 394
THEMA 398
THEAQ 390
ASPNI 421
PENCI 417
YARLI 417
TRIRE 417
TRIVI 423
NEUCR 418
CANMA 417
KLULA 416
SACCE 416
RHINI_PGK1 417
RHINI_PGK2 417
HOMSA_PGK1 417
MUSMU_PGK1 417
GALGA 417
HOMSA_PGK2 417
MUSMU_PGK2 417
SCHMA 416
DROME 415
PLAFA 416
TETTH 420
TRYBR_Gly 509
TRYCO_Gly 509
TRYCO_Cyt 420
TRYBR_Cyt1 420
TRYBR_Cyt2 431
CRIFA_Cyt 437
CRIFA_Gly 505
CORGL 405
MYCLE 410
XANFL 397
ZYMMO 397
ESCCO 387
HAEIN 386
CHLRE_Cyt 447
VOLCA_Cyt 440
METBR 406
METFE 405
PICSI_Cyt 456
MICSP_Cyt 453
HALVA 401

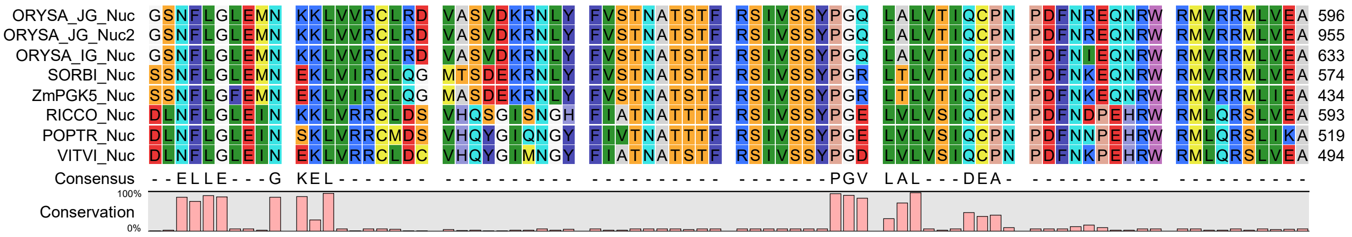
ORYSA_JG_Nuc GSNFLGLEMN KKLVVRCLRD VASVDKRNLY FVSTNATSTF RSIVSSYPGQ LALVTIQCPN PDFNREQNRW RMVRRMLVEA 596
ORYSA_JG_Nuc2 GSNFLGLEMN KKLVVRCLRD VASVDKRNLY FVSTNATSTF RSIVSSYPGQ LALVTIQCPN PDFNREQNRW RMVRRMLVEA 955
ORYSA_IG_Nuc GSNFLGLEMN KKLVVRCLRD VASVDKRNLY FVSTNATSTF RSIVSSYPGQ LALVTIQCPN PDFNIEQNRW RMVRRMLVEA 633
SORBI_Nuc SSNFLGLEMN EKLVIRCLQG MTSDEKRNLY FVSTNATSTF RSIVSSYPGR LTLVTIQCPN PDFNKEQNRW RMVRRMLVEA 574
ZmPGK5_Nuc SSNFLGFEMN EKLVIRCLQG MASDEKRNLY FVSTNATSTF RSIVSSYPGR LTLVTIQCPN PDFNKEQNRW RMVRRMLIEA 434
RICCO_Nuc DLNFLGLEIN KKLVRRCLDS VHQSGISNGH FIATNATTTF RSIVSSYPGE LVLVSIQCPN PDFNDPEHRW RMLQRSLVEA 593
POPTR_Nuc DLNFLGLEIN SKLVRRCMDS VHQYGIQNGY FIVTNATTTF RSIVSSYPGE LVLVSIQCPN PDFNNPEHRW RMLQRSLIKA 519
VITVI_Nuc DLNFLGLEIN EKLVRRCLDC VHQYGIMNGY FIATNATSTF RSIVSSYPGD LVLVSIQCPN PDFNKPEHRW RMLQRSLVEA 494
Consensus --ELLE---G KEL------- ---------- ---------- -------PGV LAL---DEA- ---------- ----------
100%
Conservation
0%

```
                                1,140                 1,160                 1,180                 1,200
                                    |                     |                     |                     |
POPNI_Cyt     ---------- ---------- ---------- ---------- ---------- ---------- ---------- ---------- 401
POPTR_Cyt2    ---------- ---------- ---------- ---------- ---------- ---------- ---------- ---------- 401
POPTR_Cyt1    ---------- ---------- ---------- ---------- ---------- ---------- ---------- ---------- 401
RICCO_Cyt     ---------- ---------- ---------- ---------- ---------- ---------- ---------- ---------- 401
GOSHI_Cyt     ---------- ---------- ---------- ---------- ---------- ---------- ---------- ---------- 401
VITVI_Cyt     ---------- ---------- ---------- ---------- ---------- ---------- ---------- ---------- 401
SOLTU_Cyt     ---------- ---------- ---------- ---------- ---------- ---------- ---------- ---------- 402
NICTA_Cyt     ---------- ---------- ---------- ---------- ---------- ---------- ---------- ---------- 401
HELAN_Cyt     ---------- ---------- ---------- ---------- ---------- ---------- ---------- ---------- 401
ARATH_Cyt     ---------- ---------- ---------- ---------- ---------- ---------- ---------- ---------- 401
PISSA_Cyt     ---------- ---------- ---------- ---------- ---------- ---------- ---------- ---------- 401
SORBI_Cyt1    ---------- ---------- ---------- ---------- ---------- ---------- ---------- ---------- 402
ZmPGK3_Cyt    ---------- ---------- ---------- ---------- ---------- ---------- ---------- ---------- 402
ORYSA_IG_Cyt  ---------- ---------- ---------- ---------- ---------- ---------- ---------- ---------- 402
ORYSA_JG_Cyt  ---------- ---------- ---------- ---------- ---------- ---------- ---------- ---------- 402
SORBI_Cyt2    ---------- ---------- ---------- ---------- ---------- ---------- ---------- ---------- 401
ORYSA_JG_Cyt2 ---------- ---------- ---------- ---------- ---------- ---------- ---------- ---------- 401
TRIAE_Cyt     ---------- ---------- ---------- ---------- ---------- ---------- ---------- ---------- 401
POPNI_Chl     ---------- -------V-- ---------- ---------- ---------- ---------- ---------- ---------- 481
POPTR_Chl2    ---------- -------V-- ---------- ---------- ---------- ---------- ---------- ---------- 481
POPTR_Chl1    ---------- -------V-- ---------- ---------- ---------- ---------- ---------- ---------- 481
RICCO_Chl     ---------- -------V-- ---------- ---------- ---------- ---------- ---------- ---------- 479
NICTA_Chl     ---------- -------V-- ---------- ---------- ---------- ---------- ---------- ---------- 481
NICBE_Chl     ---------- -------V-- ---------- ---------- ---------- ---------- ---------- ---------- 481
SOLTU_Chl     ---------- -------V-- ---------- ---------- ---------- ---------- ---------- ---------- 482
MUSAC_Chl     ---------- -------V-- ---------- ---------- ---------- ---------- ---------- ---------- 479
VITVI_Chl1    ---------- -------V-- ---------- ---------- ---------- ---------- ---------- ---------- 392
VITVI_Chl2    ---------- -------V-- ---------- ---------- ---------- ---------- ---------- ---------- 478
ARATH_Chl2    ---------- ---------- ---------- ---------- ---------- ---------- ---------- ---------- 480
ARATH_Chl1    ---------- -------V-- ---------- ---------- ---------- ---------- ---------- ---------- 478
SORBI_Chl     ---------- -------V-- ---------- ---------- ---------- ---------- ---------- ---------- 483
ZmPGK1_Chl    ---------- -------V-- ---------- ---------- ---------- ---------- ---------- ---------- 480
ZmPGK2_Chl    ---------- -------V-- ---------- ---------- ---------- ---------- ---------- ---------- 477
TRIAE_Chl     ---------- -------V-- ---------- ---------- ---------- ---------- ---------- ---------- 480
SPIOL_Chl     ---------- -------V-- ---------- ---------- ---------- ---------- ---------- ---------- 433
MUSAC_Cyt     ---------- ---------- ---------- ---------- ---------- ---------- ---------- ---------- 401
ORYSA_IG_Chl  ---------- ---------- ---------- ---------- ---------- ---------- ---------- ---------- 449
ORYSA_JG_Chl2 ---------- ---------- ---------- ---------- ---------- ---------- ---------- ---------- 429
ORYSA_JG_Chl3 ---------- ---------- ---------- ---------- ---------- ---------- ---------- ---------- 385
PICSI_Chl     ---------- -------V-- ---------- ---------- ---------- ---------- ---------- ---------- 472
CHABR         ---------- ---------- ---------- ---------- ---------- ---------- ---------- ---------- 520
CHLRE_Chl     ---------- ---------- ---------- ---------- ---------- ---------- ---------- ---------- 461
VOLCA_Chl     ---------- ---------- ---------- ---------- ---------- ---------- ---------- ---------- 462
ZmPGK4_Chl    ---------- ---------- ---------- ---------- ---------- ---------- ---------- ---------- 371
MICSP_Chl     ---------- ---------- ---------- ---------- ---------- ---------- ---------- ---------- 437
SYNSP         ---------- -------A-- ---------- ---------- ---------- ---------- ---------- ---------- 402
ORYSA_JG_Chl1 ---------- -------V-- ---------- ---------- ---------- ---------- ---------- ---------- 310
BACME         ---------- ---------- ---------- ---------- ---------- ---------- ---------- ---------- 394
BACST         ---------- ---------- ---------- ---------- ---------- ---------- ---------- ---------- 394
THEMA         ---------- ---------- ---------- ---------- ---------- ---------- ---------- ---------- 398
THEAQ         ---------- ---------- ---------- ---------- ---------- ---------- ---------- ---------- 390
ASPNI         ---------- ---------- ---------- ---------- ---------- ---------- ---------- ---------- 421
PENCI         ---------- ---------- ---------- ---------- ---------- ---------- ---------- ---------- 417
YARLI         ---------- ---------- ---------- ---------- ---------- ---------- ---------- ---------- 417
TRIRE         ---------- ---------- ---------- ---------- ---------- ---------- ---------- ---------- 417
TRIVI         ---------- ---------- ---------- ---------- ---------- ---------- ---------- ---------- 423
NEUCR         ---------- ---------- ---------- ---------- ---------- ---------- ---------- ---------- 418
CANMA         ---------- ---------- ---------- ---------- ---------- ---------- ---------- ---------- 417
KLULA         ---------- ---------- ---------- ---------- ---------- ---------- ---------- ---------- 416
SACCE         ---------- ---------- ---------- ---------- ---------- ---------- ---------- ---------- 416
RHINI_PGK1    ---------- ---------- ---------- ---------- ---------- ---------- ---------- ---------- 417
RHINI_PGK2    ---------- ---------- ---------- ---------- ---------- ---------- ---------- ---------- 417
HOMSA_PGK1    ---------- ---------- ---------- ---------- ---------- ---------- ---------- ---------- 417
MUSMU_PGK1    ---------- ---------- ---------- ---------- ---------- ---------- ---------- ---------- 417
GALGA         ---------- ---------- ---------- ---------- ---------- ---------- ---------- ---------- 417
HOMSA_PGK2    ---------- ---------- ---------- ---------- ---------- ---------- ---------- ---------- 417
MUSMU_PGK2    ---------- ---------- ---------- ---------- ---------- ---------- ---------- ---------- 417
SCHMA         ---------- ---------- ---------- ---------- ---------- ---------- ---------- ---------- 416
DROME         ---------- ---------- ---------- ---------- ---------- ---------- ---------- ---------- 415
PLAFA         ---------- ---------- ---------- ---------- ---------- ---------- ---------- ---------- 416
TETTH         ---------- ---------- ---------- ---------- ---------- ---------- ---------- ---------- 420
TRYBR_Gly     ---------- ---------- ---------- ---------- ---------- ---------- ---------- ---------- 509
TRYCO_Gly     ---------- ---------- ---------- ---------- ---------- ---------- ---------- ---------- 509
TRYCO_Cyt     ---------- ---------- ---------- ---------- ---------- ---------- ---------- ---------- 420
TRYBR_Cyt1    ---------- ---------- ---------- ---------- ---------- ---------- ---------- ---------- 420
TRYBR_Cyt2    ---------T LSNRWSSL-- ---------- ---------- ---------- ---------- ---------- ---------- 440
CRIFA_Cyt     AAVPAAATAT VSMVLASP-- ---------- ---------- ---------- ---------- ---------- ---------- 455
CRIFA_Gly     ---------- ---------- ---------- ---------- ---------- ---------- ---------- ---------- 505
CORGL         ---------- ---------- ---------- ---------- ---------- ---------- ---------- ---------- 405
MYCLE         ---------- --PRGGDS-- ---------- ---------- ---------- ---------- ---------- ---------- 416
XANFL         ---------- ---------- ---------- ---------- ---------- ---------- ---------- ---------- 397
ZYMMO         ---------- ---------- ---------- ---------- ---------- ---------- ---------- ---------- 397
ESCCO         ---------- ---------- ---------- ---------- ---------- ---------- ---------- ---------- 387
HAEIN         ---------- ---------- ---------- ---------- ---------- ---------- ---------- ---------- 386
CHLRE_Cyt     QQTAAAAPAC CAAKKCCT-- ---------- ---------- ---------- ---------- ---------- ---------- 465
VOLCA_Cyt     ---------- ---------- ---------- ---------- ---------- ---------- ---------- ---------- 440
METBR         ---------- ---GRK---- ---------- ---------- ---------- ---------- ---------- ---------- 409
METFE         ---------- ---SDKYI-- ---------- ---------- ---------- ---------- ---------- ---------- 410
PICSI_Cyt     QSEN------ ------PK-- ---------- ---------- ---------- ---------- ---------- ---------- 462
MICSP_Cyt     EKPKCMALES CDCGPTPA-- ---------- ---------- ---------- ---------- ---------- ---------- 471
HALVA         ---------- ---------- ---------- ---------- ---------- ---------- ---------- ---------- 401
```

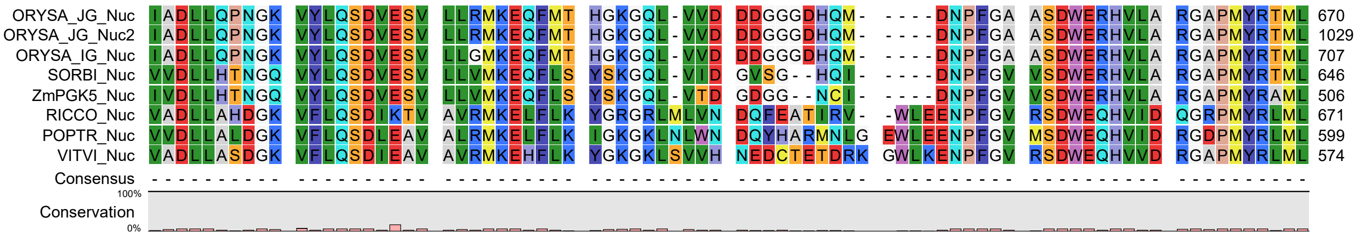
ORYSA_JG_Nuc IADLLQPNGK VYLQSDVESV LLRMKEQFMT HGKGQL-VVD DDGGGDHQM- ----DNPFGA ASDWERHVLA RGAPMYRTML 670
ORYSA_JG_Nuc2 IADLLQPNGK VYLQSDVESV LLRMKEQFMT HGKGQL-VVD DDGGGDHQM- ----DNPFGA ASDWERHVLA RGAPMYRTML 1029
ORYSA_IG_Nuc IADLLQPNGK VYLQSDVESV LLGMKEQFMT HGKGQL-VVD DDGGGDHQM- ----DNPFGA ASDWERHVLA RGAPMYRTML 707
SORBI_Nuc VVDLLHTNGQ VYLQSDVESV LLVMKEQFLS YSKGQL-VID GVSG--HQI- ----DNPFGV VSDWERHVLA RGAPMYRTML 646
ZmPGK5_Nuc IVDLLHTNGQ VYLQSDVESV LLVMKEQFLS YSKGQL-VTD GDGG--NCI- ----DNPFGV VSDWERHVLA RGAPMYRAML 506
RICCO_Nuc VADLLAHDGK VFLQSDIKTV AVRMKELFLK YGRGRLMLVN DQFEATIRV- -WLEENPFGV RSDWEQHVID QGRPMYRLML 671
POPTR_Nuc VVDLLALDGK VFLQSDLEAV ALRMKELFLK IGKGKLNLWN DQYHARMNLG EWLEENPFGV MSDWEQHVID RGDPMYRLML 599
VITVI_Nuc VADLLASDGK VFLQSDIEAV AVRMKEHFLK YGKGKLSVVH NEDCTETDRK GWLKENPFGV RSDWEQHVVD RGAPMYRLML 574
Consensus
100%
Conservation
0%

POPNI_Cyt	- - - - - - -	401
POPTR_Cyt2	- - - - - - -	401
POPTR_Cyt1	- - - - - - -	401
RICCO_Cyt	- - - - - - -	401
GOSHI_Cyt	- - - - - - -	401
VITVI_Cyt	- - - - - - -	401
SOLTU_Cyt	- - - - - - -	402
NICTA_Cyt	- - - - - - -	401
HELAN_Cyt	- - - - - - -	401
ARATH_Cyt	- - - - - - -	401
PISSA_Cyt	- - - - - - -	401
SORBI_Cyt1	- - - - - - -	402
ZmPGK3_Cyt	- - - - - - -	402
ORYSA_IG_Cyt	- - - - - - -	402
ORYSA_JG_Cyt	- - - - - - -	402
SORBI_Cyt2	- - - - - - -	401
ORYSA_JG_Cyt2	- - - - - - -	401
TRIAE_Cyt	- - - - - - -	401
POPNI_Chl	- - - - - - -	481
POPTR_Chl2	- - - - - - -	481
POPTR_Chl1	- - - - - - -	481
RICCO_Chl	- - - - - - -	479
NICTA_Chl	- - - - - - -	481
NICBE_Chl	- - - - - - -	481
SOLTU_Chl	- - - - - - -	482
MUSAC_Chl	- - - - - - -	479
VITVI_Chl1	- - - - - - -	392
VITVI_Chl2	- - - - - - -	478
ARATH_Chl2	- - - - - - -	480
ARATH_Chl1	- - - - - - -	478
SORBI_Chl	- - - - - - -	483
ZmPGK1_Chl	- - - - - - -	480
ZmPGK2_Chl	- - - - - - -	477
TRIAE_Chl	- - - - - - -	480
SPIOL_Chl	- - - - - - -	433
MUSAC_Cyt	- - - - - - -	401
ORYSA_IG_Chl	- - - - - - -	449
ORYSA_JG_Chl2	- - - - - - -	429
ORYSA_JG_Chl3	- - - - - - -	385
PICSI_Chl	- - - - - - -	472
CHABR	- - - - - - -	520
CHLRE_Chl	- - - - - - -	461
VOLCA_Chl	- - - - - - -	462
ZmPGK4_Chl	- - - - - - -	371
MICSP_Chl	- - - - - - -	437
SYNSP	- - - - - - -	402
ORYSA_JG_Chl1	- - - - - - -	310
BACME	- - - - - - -	394
BACST	- - - - - - -	394
THEMA	- - - - - - -	398
THEAQ	- - - - - - -	390
ASPNI	- - - - - - -	421
PENCI	- - - - - - -	417
YARLI	- - - - - - -	417
TRIRE	- - - - - - -	417
TRIVI	- - - - - - -	423
NEUCR	- - - - - - -	418
CANMA	- - - - - - -	417
KLULA	- - - - - - -	416
SACCE	- - - - - - -	416
RHINI_PGK1	- - - - - - -	417
RHINI_PGK2	- - - - - - -	417
HOMSA_PGK1	- - - - - - -	417
MUSMU_PGK1	- - - - - - -	417
GALGA	- - - - - - -	417
HOMSA_PGK2	- - - - - - -	417
MUSMU_PGK2	- - - - - - -	417
SCHMA	- - - - - - -	416
DROME	- - - - - - -	415
PLAFA	- - - - - - -	416
TETTH	- - - - - - -	420
TRYBR_Gly	- - - - - - -	509
TRYCO_Gly	- - - - - - -	509
TRYCO_Cyt	- - - - - - -	420
TRYBR_Cyt1	- - - - - - -	420
TRYBR_Cyt2	- - - - - - -	440
CRIFA_Cyt	- - - - - - -	455
CRIFA_Gly	- - - - - - -	505
CORGL	- - - - - - -	405
MYCLE	- - - - - - -	416
XANFL	- - - - - - -	397
ZYMMO	- - - - - - -	397
ESCCO	- - - - - - -	387
HAEIN	- - - - - - -	386
CHLRE_Cyt	- - - - - - -	465
VOLCA_Cyt	- - - - - - -	440
METBR	- - - - - - -	409
METFE	- - - - - - -	410
PICSI_Cyt	- - - - - - -	462
MICSP_Cyt	- - - - - - -	471
HALVA	- - - - - - -	401

ORYSA_JG_Nuc	RKV----	673
ORYSA_JG_Nuc2	RKV----	1032
ORYSA_IG_Nuc	RKV----	710
SORBI_Nuc	KKV----	649
ZmPGK5_Nuc	KKM----	509
RICCO_Nuc	SKSTNGE	678
POPTR_Nuc	SKSSGIE	606
VITVI_Nuc	SKSTRPN	581
Consensus	-------	

Conservation

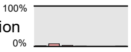

Supplement: Supplementary file 1 [file plants-09-01639-s001.zip › Supplementary Materials/Figure S1.pdf]
