# Supplementary material for: The Phosphoglycerate Kinase (PGK) Gene Family of Maize (Zea mays var. B73)
Source: Plants (Basel). 2020 Nov 24;9(12):1639. doi: 10.3390/plants9121639 (PMC7761438; doi:10.3390/plants9121639)
Supplement: Supplementary file 1 [file plants-09-01639-s001.zip › Supplementary Materials/Figure S7.pdf]

Figure S7 Comparison (A) and sequence analysis (B) of nuclear PGKs from plants with an additional methyltransferase domain.

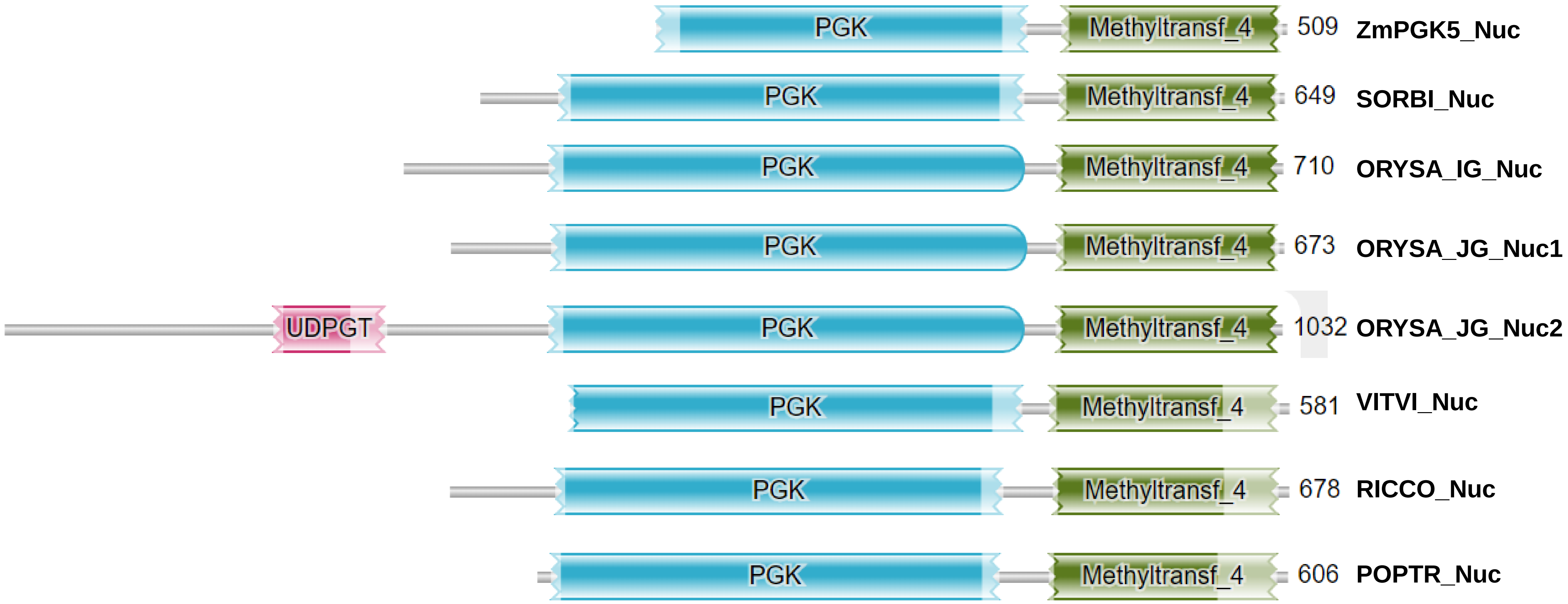

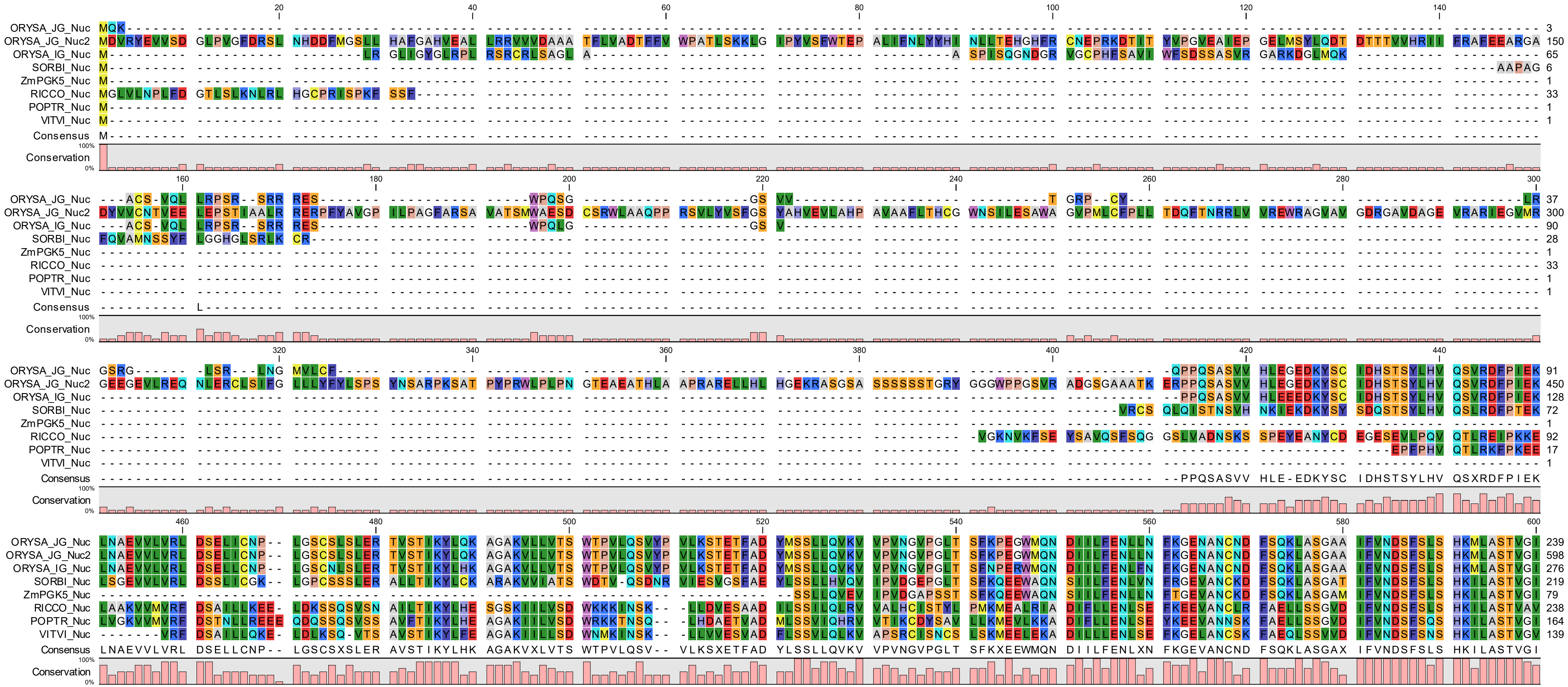
ORYSA_JG_Nuc
ORYSA_JG_Nuc2
ORYSA_IG_Nuc
SORBI_Nuc
ZmPGK5_Nuc
RICCO_Nuc
POPTR_Nuc
VITVI_Nuc
Consensus
Conservation
100%
0%

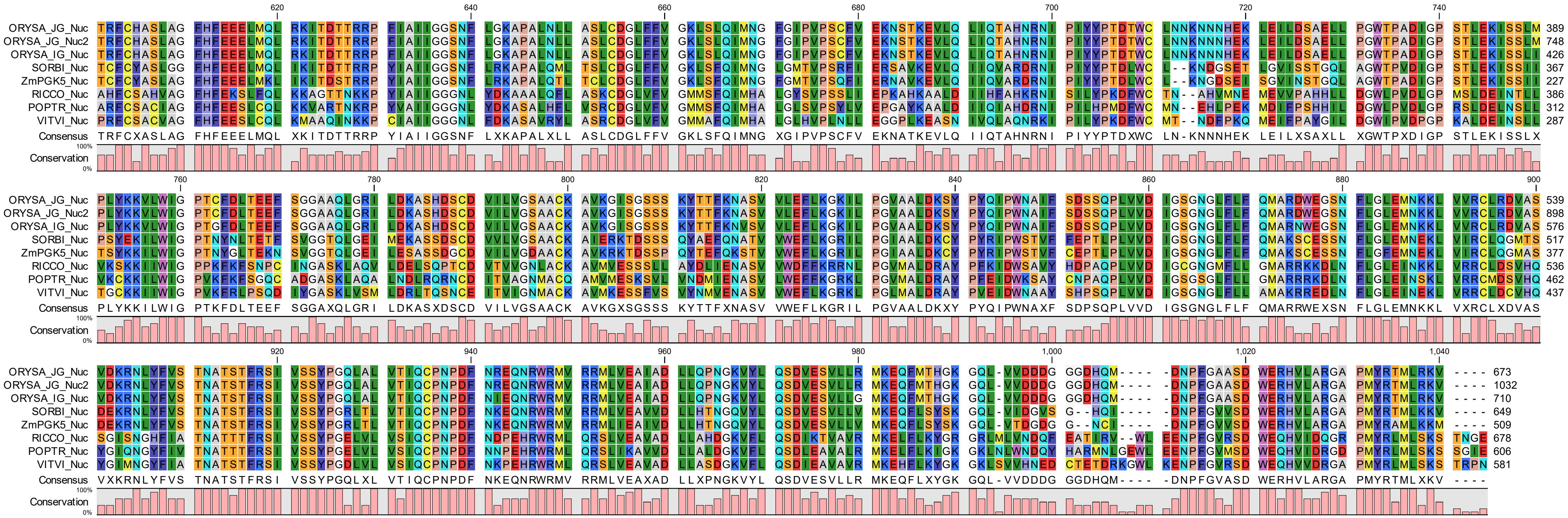
620 640 660 680 700 720 740
ORYSA_JG_Nuc TRFCHASLAG FHFEEELMQL RKITDTTRRP FIAIIGGSNF LGKAPALNLL ASLCDGLFFV GKLSLQIMNG FGIPVPSCFV EKNSTKEVLQ LIQTAHNRNI PIYYPTDTWC LNNKNNNHEK LEILDSAELL PGWTPADIGP STLEKISSLM 389
ORYSA_JG_Nuc2 TRFCHASLAG FHFEEELMQL RKITDTTRRP FIAIIGGSNF LGKAPALNLL ASLCDGLFFV GKLSLQIMNG FGIPVPSCFV EKNSTKEVLQ LIQTAHNRNI PIYYPTDTWC LNNKNNNHEK LEILDSAELL PGWTPADIGP STLEKISSLM 748
ORYSA_IG_Nuc TRFCHASLAG FHFEEELMQL RKITDTTRRP FIAIIGGSNF LGKAPALNLL ASLCDGLFFV GKLSLQIMNG FGIPVPSCFV EKNSTKEVLQ LIQTAHNRNI PIYYPTDTWC LNNKNNNHEK LEILDSAELL PGWTPADIGP STLEKISSLI 426
SORBI_Nuc TCFCYASLGG FHFEEELMQL IKITDTTRRP YIAIIGGSNF LRKAPALQML TSLCDGLFFV GKLSFQIMNG LGMTVPSRFI ERSAVKEVLQ IIQVARDRNI PIYYPTDLWC L--KNDGSET LGVISSTGQL AGWTPVDIGP STLEKISSII 367
ZmPGK5_Nuc TCFCYASLAG FHFEEELMKL IKITDSTRRP YIAIIGGSNF LRKAPALQTL TCLCDGLFFV GKLSFQIMNG FGMTVPSQFI ERNAVKEVLQ IIQVARDRNI PIYYPTDLWC L--KNGDSEI SGVINSTGQL AGWTPADIGP STLEKISSII 227
RICCO_Nuc AHFCSAHVAG FHFEKSLFQL KKAGTTNKKP YIAIIGGGNL YDKAAALQFL ASKCDGLVFV GMMSFQIMHA LGYSVPSSLI EPKAHKAALD IIHFAHKRNI SILYPKDFWC TN--AHVMNE MEVVPAHHLL DGWLPVDLGP MSLDEINTLL 386
POPTR_Nuc ARFCSACIAG FHFEESLCQL KKVARTNKRP YVAIIGGGNL YDKASALHFL VSRCDGLVFV GMMSFQIMHA LGLSVPSYLV EPGAYKAALD IIQIAHDRNI PILHPMDFWC MN--EHLPEK MDIFPSHHIL DGWLPVDLGP RSLDELNSLL 312
VITVI_Nuc PRFCSACVAG FHFEESLCQL KMAAQINKKP CIAIIGGGNL FDKASAVRYL ASRCDGLVFV GMMAFQIMHA LGLHVPLNLL EGGPLKEASN IVQLAQNRKI PILYPKDFWC MT--NDFPKQ MEIFPAYGIL DGWIPVDPGP KALDEINSLL 287
Consensus TRFCXASLAG FHFEEELMQL XKITDTTRRP YIAIIGGSNF LXKAPALXLL ASLCDGLFFV GKLSFQIMNG XGIPVPSCFV EKNATKEVLQ IIQTAHNRNI PIYYPTDXWC LN-KNNNHEK LEILXSAXLL XGWTPXDIGP STLEKISSLX
100%
Conservation
0%
760 780 800 820 840 860 880 900
ORYSA_JG_Nuc PLYKKVLWIG PTCFDLTEEF SGGAAQLGRI LDKASHDSCD VILVGSAACK AVKGISGSSS KYTTFKNASV VLEFLKGKIL PGVAALDKSY PYQIPWNAIF SDSSQPLVVD IGSGNGLFLF QMARDWEGSN FLGLEMNKKL VVRCLRDVAS 539
ORYSA_JG_Nuc2 PLYKKVLWIG PTCFDLTEEF SGGAAQLGRI LDKASHDSCD VILVGSAACK AVKGISGSSS KYTTFKNASV VLEFLKGKIL PGVAALDKSY PYQIPWNAIF SDSSQPLVVD IGSGNGLFLF QMARDWEGSN FLGLEMNKKL VVRCLRDVAS 898
ORYSA_IG_Nuc PLYKKVLWIG PTGFDLTEEF SGGAAQLGRI LDKASHDSCD VILVGSAACK AVKGISGSSS KYTTFKNVSV VLEFLKGKIL PGVAALDKSY PYQIPWNAIF SDSSQPLVVD IGSGNGLFLF QMARNWEGSN FLGLEMNKKL VVRCLRDVAS 576
SORBI_Nuc PSYEKILWIG PTNYNLTETF SVGGTQLGEI MEKASSDSCD VVLVGSAACK AIERKTDSSS QYAEFQNATV VWEFLKGRIL PGIAALDKCY PYRIPWSTVF FEPTLPLVVD IGSGNGLFLF QMAKSCESSN FLGLEMNEKL VIRCLQGMTS 517
ZmPGK5_Nuc TSYKKILWIG PTNYGLTEKN SVGGTQLGEI LESASSDGCD VILVGDAACK AVKRKTDSSP QYTEFQKSTV VWEFLKGRIL PGIAALDKCY PYRIPWSTVF CEPTLPLVVD IGSGNGLFLF QMAKSCESSN FLGFEMNEKL VIRCLQGMAS 377
RICCO_Nuc VKSKKIIWIG PPKFKFSNPC INGASKLAQV LDELSQPTCD VTVVGNLACK AVMVESSSLL AYDLIENASV VWDFFKRRNL PGVMALDRAY PFKIDWSAVY HDPAQPLVVD IGCGNGMFLL GMARRKKDLN FLGLEINKKL VRRCLDSVHQ 536
POPTR_Nuc VKCKKILWIG PVKFKFSGQC ADGASKLAQA LNDLRQRNCD ITVAGNMACQ AMVMESKSVL VNDMIENASV LWEFFKGRKL PGVMALDRAY PFEIDWKSAY CNPAQPLVVD IGSGSGLFLL GMARRRKDLN FLGLEINSKL VRRCMDSVHQ 462
VITVI_Nuc TGCKKIIWIG PVKFRLPSQD IYGASKLVSM LDRLTQSNCE ITVIGNMACK AVMKESSFVS VYNMVENASV VWEFLKGRKL PGLMALDRAY PVEIDWNAAY SHPSQPLVVD IGSGNGLFLL AMAKRREDLN FLGLEINEKL VRRCLDCVHQ 437
Consensus PLYKKILWIG PTKFDLTEEF SGGAXQLGRI LDKASXDSCD VILVGSAACK AVKGXSGSSS KYTTFXNASV VWEFLKGRIL PGVAALDKXY PYQIPWNAXF SDPSQPLVVD IGSGNGLFLF QMARRWEXSN FLGLEMNKKL VXRCLXDVAS
100%
Conservation
0%
920 940 960 980 1,000 1,020 1,040
ORYSA_JG_Nuc VDKRNLYFVS TNATSTFRSI VSSYPGQLAL VTIQCPNPDF NREQNRWRMV RRMLVEAIAD LLQPNGKVYL QSDVESVLLR MKEQFMTHGK GQL-VVDDDG GGDHQM---- -DNPFGAASD WERHVLARGA PMYRTMLRKV ---- 673
ORYSA_JG_Nuc2 VDKRNLYFVS TNATSTFRSI VSSYPGQLAL VTIQCPNPDF NREQNRWRMV RRMLVEAIAD LLQPNGKVYL QSDVESVLLR MKEQFMTHGK GQL-VVDDDG GGDHQM---- -DNPFGAASD WERHVLARGA PMYRTMLRKV ---- 1032
ORYSA_IG_Nuc VDKRNLYFVS TNATSTFRSI VSSYPGQLAL VTIQCPNPDF NIEQNRWRMV RRMLVEAIAD LLQPNGKVYL QSDVESVLLG MKEQFMTHGK GQL-VVDDDG GGDHQM---- -DNPFGAASD WERHVLARGA PMYRTMLRKV ---- 710
SORBI_Nuc DEKRNLYFVS TNATSTFRSI VSSYPGRLTL VTIQCPNPDF NKEQNRWRMV RRMLVEAVVD LLHTNGQVYL QSDVESVLLV MKEQFLSYSK GQL-VIDGVS G--HQI---- -DNPFGVVSD WERHVLARGA PMYRTMLKKV ---- 649
ZmPGK5_Nuc DEKRNLYFVS TNATSTFRSI VSSYPGRLTL VTIQCPNPDF NKEQNRWRMV RRMLIEAIVD LLHTNGQVYL QSDVESVLLV MKEQFLSYSK GQL-VTDGDG G--NCI---- -DNPFGVVSD WERHVLARGA PMYRAMLKKM ---- 509
RICCO_Nuc SGISNGHFIA TNATTTFRSI VSSYPGELVL VSIQCPNPDF NDPEHRWRML QRSLVEAVAD LLAHDGKVFL QSDIKTVAVR MKELFLKYGR GRLMLVNDQF EATIRV--WL EENPFGVRSD WEQHVIDQGR PMYRLMLSKS TNGE 678
POPTR_Nuc YGIQNGYFIV TNATTTFRSI VSSYPGELVL VSIQCPNPDF NNPEHRWRML QRSLIKAVVD LLALDGKVFL QSDLEAVALR MKELFLKIGK GKLNLWNDQY HARMNLGEWL EENPFGVMSD WEQHVIDRGD PMYRLMLSKS SGIE 606
VITVI_Nuc YGIMNGYFIA TNATSTFRSI VSSYPGDLVL VSIQCPNPDF NKPEHRWRML QRSLVEAVAD LLASDGKVFL QSDIEAVAVR MKEHFLKYGK GKLSVVHNED CTETDRKGWL KENPFGVRSD WEQHVVDRGA PMYRLMLSKS TRPN 581
Consensus VXKRNLYFVS TNATSTFRSI VSSYPGQLXL VTIQCPNPDF NKEQNRWRMV RRMLVEAXAD LLXPNGKVYL QSDVESVLLR MKEQFLXYGK GQL-VVDDDG GGDHQM---- -DNPFGVASD WERHVLARGA PMYRTMLXKV ----
100%
Conservation
0%
