# Supplementary figures and images for: The Phosphoglycerate Kinase (PGK) Gene Family of Maize (Zea mays var. B73)
Source: Plants (Basel). 2020 Nov 24;9(12):1639. doi: 10.3390/plants9121639 (PMC7761438; doi:10.3390/plants9121639)

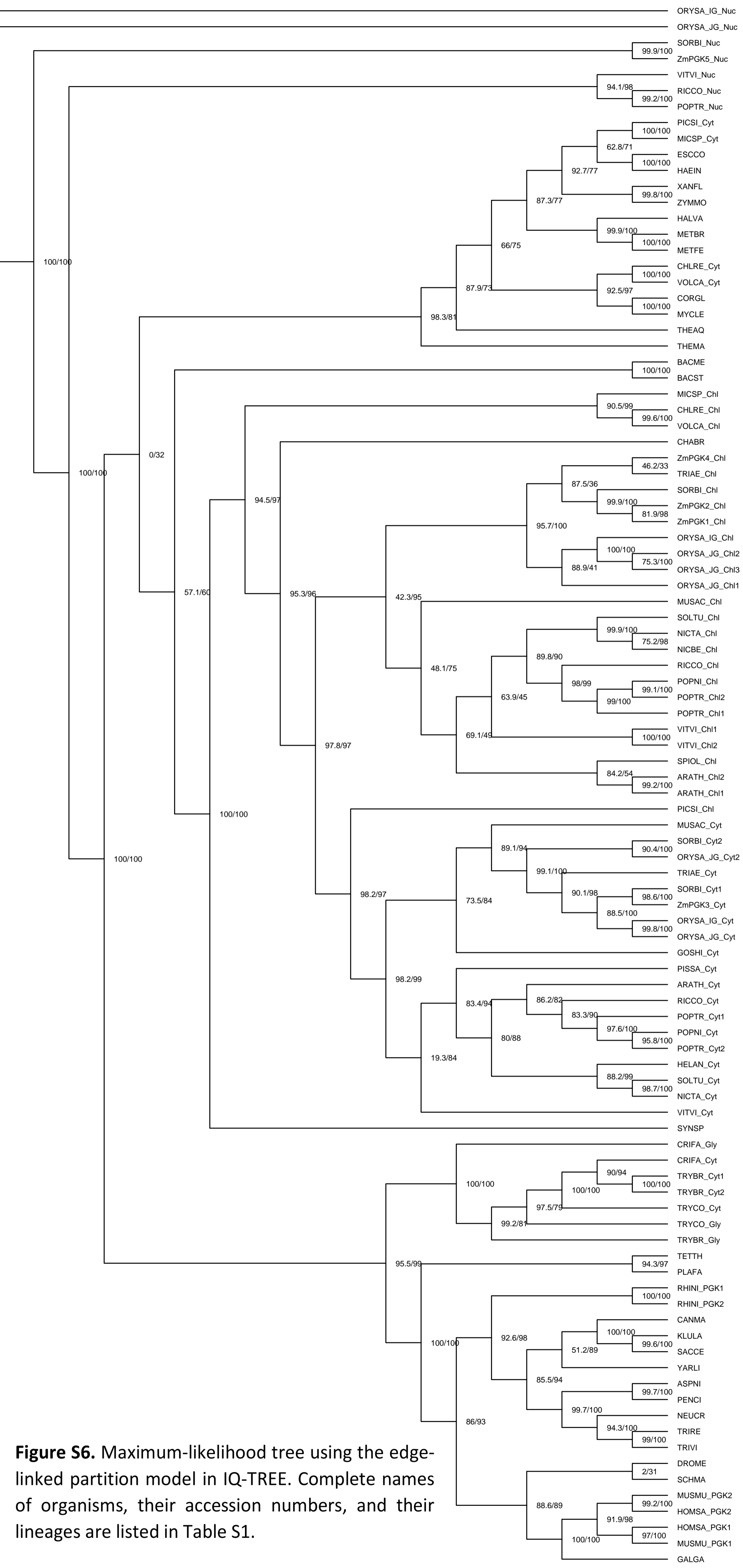

Supplement: Supplementary file 1 [file plants-09-01639-s001.zip › Supplementary Materials/Figure S6.pdf]
