# Supplementary material for: The Phosphoglycerate Kinase (PGK) Gene Family of Maize (Zea mays var. B73)
Source: Plants (Basel). 2020 Nov 24;9(12):1639. doi: 10.3390/plants9121639 (PMC7761438; doi:10.3390/plants9121639)
Supplement: Supplementary file 1 [file plants-09-01639-s001.zip › Supplementary Materials/Figure S2.doc]

**Figure S2** Overview of the *ZmPgk* gene family of *Z. mays* var B73. The chromosomes reflect segmental duplications (shown in gray) that are characteristic of an evolutionary process that involved diploidisation of an ancestor genome with five chromosomes, followed by extensive genomic rearrangements that led to the 10 heterochromosomes of modern maize (Schnable et al., 2009). Plastidial *ZmPgk1* and *ZmPgk2* most likely originated from endoduplication at synthetic positions on Chr 6 and 8. Cytosolic *ZmPgk3* was located on Chr 5, whereas nuclear *ZmPgk5* was located on Chr 1 (nonsynthenic positions).
