# Supplementary material for: The Phosphoglycerate Kinase (PGK) Gene Family of Maize (Zea mays var. B73)
Source: Plants (Basel). 2020 Nov 24;9(12):1639. doi: 10.3390/plants9121639 (PMC7761438; doi:10.3390/plants9121639)
Supplement: Supplementary file 1 [file plants-09-01639-s001.zip › Supplementary Materials/Figure S3.doc]

**Figure S3** Comparison of absolute gene expression between *ZmPgk genes* and EF1α reference gene. Absolute quantification and comparison of expression levels between *ZmPgks* and EF1α (reference gene) in R1 and R3 stages are shown in A and B, respectively. The tissues analysed for each gene were leaf blade (LB: 1), leaf sheath (LS: 2), node (ND: 3), and internode (IN: 4), husk leaf (HL: 5), peduncle (PD: 6), corncob (CC: 7), grain (GR: 8) and root (RT: 9). Bar represents the mean ± standard error (SE) with n=3
