# Supplementary material for: The Phosphoglycerate Kinase (PGK) Gene Family of Maize (Zea mays var. B73)
Source: Plants (Basel). 2020 Nov 24;9(12):1639. doi: 10.3390/plants9121639 (PMC7761438; doi:10.3390/plants9121639)
Supplement: Supplementary file 1 [file plants-09-01639-s001.zip › Supplementary Materials/Figure S4.doc]

**Figure S4** Michaelis Menten kinetic to estimate substrate affinity to 3PGA and/or ATP for maize tissue. Substrate affinity was measured in five maize tissues at R1 stage; Leaf blade= LB, Husk leaf= HL, Stem= ST (internode + node), Corncob= CC and Root= RT. Values = the average of three replicates (±SD) in units of nmol/seg.
