# Supplementary material for: The Phosphoglycerate Kinase (PGK) Gene Family of Maize (Zea mays var. B73)
Source: Plants (Basel). 2020 Nov 24;9(12):1639. doi: 10.3390/plants9121639 (PMC7761438; doi:10.3390/plants9121639)
Supplement: Supplementary file 1 [file plants-09-01639-s001.zip › Supplementary Materials/Figure S8.doc]

**Figure S8.** Maximum likelihood tree of 99 PGK protein sequences, including nuclear PGKs without methyltransferase domain. Phylogram was constructed from a WAG model that used a gamma distribution for the variability of substitution rate across position. Numbers in nodes represent the bootstrap proportion for 1,000 replicates of the same tree construction method. PGKs are coloured according to the taxa as follow: Plants (green), Algae (yellow green), Fungi (purple), Protozoa (Navy blue), Animal (red), Bacteria (aquamarine) and Archaea (Black). Complete names of organisms, their accession numbers, and their lineages are listed in Table S1.
