# Supplementary material for: The Phosphoglycerate Kinase (PGK) Gene Family of Maize (Zea mays var. B73)
Source: Plants (Basel). 2020 Nov 24;9(12):1639. doi: 10.3390/plants9121639 (PMC7761438; doi:10.3390/plants9121639)
Supplement: Supplementary file 1 [file plants-09-01639-s001.zip › Supplementary Materials/Table S2.doc]

**Table S2 Oligonucleotides for qRT-PCR**

| **Target Gene** | **Primer Name** | **Sequence 5´→ 3´** | **Expected amplicon size (bp)** | **Tm ºC** |
| --- | --- | --- | --- | --- |
| EF1 α | EF1α_For | GGGCGTGTTGAGACTGGTAT | 150 | 63.7 |
|  | EF1α_Rev | AGGCCACTAGGACCAAAGGT |  | 63.4 |
| ZmPGK5 | 3724_For | CCTGGACGGTTGACTCTTGT | 138 | 64.12 |
|  | 3724_Rev | CAGATAAACCTGTCCATTGGTATG |  | 63.01 |
| ZmPGK4 | 7028_For | CAGTTCTGCCCTGGAAACTAC | 116 | 62.54 |
|  | 7028_Rev | TAAGCTCGGCTAACTTCTTTGC |  | 63.58 |
| ZmPGK3 | 2914_For | TGTTATTTGGAACGGCCCC | 109 | 66.59 |
|  | 2914_Rev | AGTGACACCCTTCGTGGTTGTAA |  | 66.47 |
| ZmPGK2 | 3016New_For | CACCGGAGGTGGTGCC | 186 | 66.8 |
|  | 3016New_Rev | GCTGCTTCATCCAATGCAGT |  | 65.28 |
| ZmPGK1 | 9136_For | CAGAAGTTCGCTGTACCAACC | 216 | 64.83 |
|  | 9136_Rev | GGCTTCATTCAACGCAACA |  | 64.83 |
