# Supplementary material for: The Phosphoglycerate Kinase (PGK) Gene Family of Maize (Zea mays var. B73)
Source: Plants (Basel). 2020 Nov 24;9(12):1639. doi: 10.3390/plants9121639 (PMC7761438; doi:10.3390/plants9121639)
Supplement: Supplementary file 1 [file plants-09-01639-s001.zip › Supplementary Materials/Table S3.doc]

**Table S3** Number of motifs in the upstream 1000-bp of the maize and Arabidopsis promoter sequences. *Cis*-elements were categorized in chloroplast, light-regulated, endosperm and stress response gene regulations

|  | | **ZmPGK1** | **ZmPGK2** | **AtPGK1** | **AtPGK2** | **ZmPGK4** | **ZmPGK3** | **AtPGK3** | **ZmPGK5** |
| --- | --- | --- | --- | --- | --- | --- | --- | --- | --- |
|  |  |  |  |  |  |  |  |  |  |
| **Cis-elements** | **Motif sequence** | **Plastdial** | | | | | **Cytoplasmic** | | **Nuclear** |
| Chloroplast | | | | | | | | | |
| CACTFTPPCA1 | YACT | 14 | 7 | 15 | 14 | 8 | 9 | 17 | 11 |
| GATABOX | GATA | 4 | 3 | 8 | 10 | 5 | 6 | 4 | 7 |
| -10PEHVPSBD | TATTCT | 1 | 3 | 0 | 0 | 2 | 0 | 0 | 2 |
| PRECONSCRHSP70A | SCGAYNRN14HD | 2 | 5 | 0 | 1 | 1 | 4 | 2 | 2 |
| BOXIINTPATPB | ATAGAA | 2 | 1 | 0 | 0 | 3 | 0 | 0 | 1 |
| Light-regulated | | | | | | | | | |
| ASF1MOTIFCAMV | TGACG | 1 | 6 | 0 | 0 | 3 | 0 | 2 | 0 |
| IBOXCORE | GATAA | 2 | 0 | 5 | 0 | 2 | 2 | 3 | 3 |
| TAAAGSTKST1 | TAAAG | 2 | 5 | 2 | 6 | 1 | 1 | 1 | 0 |
| SORLIP1AT | GCCAC | 3 | 0 | 0 | 0 | 6 | 0 | 0 | 0 |
| CIACADIANLELHC | CAAN4ATC | 0 | 1 | 2 | 0 | 0 | 2 | 2 | 0 |
| GT1CONSENSUS | GRWAAW | 5 | 3 | 5 | 6 | 9 | 0 | 0 | 1 |
| SORLIP2AT | GGGCC | 0 | 4 | 0 | 3 | 0 | 9 | 0 | 3 |
| INRNTPSADB | YTCANTYY | 2 | 2 | 3 | 4 | 1 | 0 | 3 | 0 |
| Endosperm | | | | | | | | | |
| DOFCOREZM | AAAG | 10 | 8 | 15 | 15 | 8 | 8 | 13 | 5 |
| EBOXBNNAPA | CANNTG | 4 | 6 | 8 | 12 | 4 | 6 | 6 | 10 |
| SEF4MOTIFGM7S | RTTTTTR | 2 | 1 | 1 | 0 | 5 | 0 | 1 | 1 |
| GCN4OSGLUB1 | TGAGTCA | 0 | 0 | 0 | 1 | 0 | 0 | 9 | 0 |
| ACGTCBOX | GACGTC | 0 | 4 | 0 | 0 | 4 | 0 | 2 | 0 |
| Stress Reponses | | | | | | | | | |
| ANAERO2CONSENSUS | AGCAGC | 3 | 1 | 0 | 3 | 0 | 5 | 1 | 1 |
| CURECORECR | GTAC | 6 | 4 | 8 | 6 | 2 | 0 | 2 | 4 |
| LTRECOREATCOR15 | CCGAC | 0 | 4 | 0 | 0 | 0 | 1 | 0 | 2 |
| MYCCONSENSUSAT | CANNTG | 4 | 6 | 8 | 12 | 4 | 6 | 6 | 12 |
| ACGTATERD1 | ACGT | 2 | 6 | 2 | 0 | 6 | 0 | 2 | 0 |
| CCAATBOX1 | CCAAT | 1 | 3 | 6 | 2 | 1 | 1 | 5 | 2 |
| GT1GMSCAM4 | GAAAAA | 1 | 0 | 1 | 1 | 5 | 6 | 4 | 0 |
| GCCCORE | GCCGCC | 3 | 2 | 0 | 0 | 1 | 3 | 0 | 1 |
| ARR1AT | NGATT | 11 | 6 | 11 | 11 | 7 | 6 | 17 | 6 |
| SURECOREATSULTR11 | GAGAC | 2 | 1 | 3 | 3 | 2 | 4 | 1 | 0 |
| WBOXNTERF3 | TGACY | 4 | 7 | 3 | 7 | 0 | 5 | 4 | 1 |
| CAATBOX1 | CAAT | 7 | 11 | 10 | 12 | 8 | 4 | 10 | 6 |
| Others (Phytomhormones, Development, Flowering, Polen…) | | | | | | | | | |
| CGACGOSAMY3 | CGACG | 4 | 7 | 2 | 0 | 2 | 5 | 1 | 4 |
| CGCGBOXAT | VCGCGB | 2 | 3 | 2 | 0 | 5 | 8 | 0 | 6 |
| HEXAMERATH4 | CCGTCG | 3 | 1 | 1 | 0 | 1 | 3 | 1 | 0 |
| BIHD1OS | TGTCA | 0 | 1 | 0 | 0 | 2 | 1 | 0 | 4 |
| NODCON2GM | CTCTT | 2 | 1 | 6 | 8 | 4 | 4 | 8 | 2 |
| OSE2ROOTNODULE | CTCTT | 2 | 1 | 6 | 8 | 2 | 4 | 8 | 2 |
| TATABOXOSPAL | TATTTAA | 0 | 0 | 3 | 4 | 0 | 0 | 1 | 3 |
| EECCRCAH1 | GANTTNC | 5 | 2 | 3 | 4 | 1 | 0 | 1 | 1 |
| POLASIG | AATAAA | 1 | 4 | 2 | 5 | 2 | 2 | 2 | 3 |
| PREATPRODH | ACTCATACTCAT | 0 | 0 | 2 | 3 | 0 | 1 | 2 | 1 |
| MYBPZM | CCWACC | 1 | 0 | 0 | 0 | 7 | 0 | 0 | 8 |
| ROOTMOTIFTAPOX1 | ATATT | 1 | 2 | 4 | 5 | 4 | 5 | 4 | 5 |
| TATABOX5 | TTATTT | 4 | 5 | 4 | 0 | 1 | 3 | 5 | 0 |
| ABRERATCAL | MACGYGB | 3 | 0 | 0 | 0 | 3 | 1 | 0 | 0 |
| WRKY71OS | TGAC | 3 | 10 | 2 | 3 | 5 | 6 | 3 | 6 |
| CARGCW8GAT | CWWWWWWWWG | 2 | 0 | 2 | 2 | 0 | 2 | 0 | 0 |
| MYBST1 | GGATA | 0 | 11 | 0 | 14 | 0 | 8 | 5 | 0 |
| GTGANTG10 | GTGA | 6 | 5 | 12 | 8 | 13 | 8 | 8 | 5 |
| POLLEN1LELAT52 | AGAAA | 5 | 1 | 6 | 4 | 3 | 2 | 7 | 2 |
| RAV1AAT | CAACA | 0 | 1 | 2 | 2 | 2 | 3 | 2 | 1 |
